# Targeting psychological stress-steroid-MARCH1 signaling pathway promotes the efficacy of specific allergen immunotherapy

**DOI:** 10.7150/thno.78851

**Published:** 2022-11-08

**Authors:** Xiang He, Jie Liu, Xiaojun Xiao, Shuang Zhang, Xinxin Wang, Jiangqi Liu, Zhiqiang Liu, Junyi Wang, Xiaoyu Liu, Guoping Li, Pingchang Yang

**Affiliations:** 1 Laboratory of Allergy and Precision Medicine, Chengdu Institute of Respiratory Health, the Third People's Hospital of Chengdu, Affiliated Hospital of Southwest Jiaotong University, Chengdu, China.; 2 Guangdong Provincial Key Laboratory of Regional Immunity and Diseases, Shenzhen, China.; 3 Institute of Allergy & Immunology, Shenzhen University School of Medicine, State Key Laboratory of Respiratory Disease Allergy Division at Shenzhen University, Shenzhen, China.; 4 Longgang ENT Hospital & Shenzhen ENT Institute, Shenzhen, China.

**Keywords:** allergy, immunotherapy, psychological stress, immune regulation, dendritic cells.

## Abstract

**Background**: The therapeutic efficacy of allergen specific immunotherapy (SIT) is recognized, but needs improved. Psychological stress influences the immune system's function. The objective of this study is to elucidate the effects of psychological stress on compromising the effectiveness of SIT.

**Methods:** A murine model with the airway allergic disorder (AAD) was established. Mice were treated with SIT with or without restraint stress (Rs).

**Results:** Rs was found to significantly hamper the efficacy of SIT in mice with AAD. Induction of IL-10^+^ dendritic cells and type 1 regulatory T cells were reduced by Rs in the airway tissues. Rs-induced cortisol release subverted immune tolerance generation. Expression of MARCH1 was elevated in dendritic cells of the allergic lesion sites. The Rs-induced MARCH1 mediated the immune impairment in AAD mice. Genetic ablation of MARCH1 in dendritic cells efficiently blocked the Rs-compromised the therapeutic efficacy of SIT.

**Conclusion**: Rs can increase the expression of MARCH1 in DCs of the allergic lesion sites. MARCH1 interferes with the immune regulatory properties in DCs, and impairs the immune regulatory capacity. Blocking MARCH1 can counteract the Rs-affected SIT efficacy.

## Introduction

Allergic diseases are abnormal immunological responses to innocent antigens. The primary pathological characteristic of the allergy is the Th2 bias and the strong production of IgE antibodies 1. IgE is a canonical mediator in triggering an allergic reaction. By binding the high affinity IgE receptors, IgE sensitizes mast cells. Re-exposure to a specific antigen activates sensitized mast cells. Allergenic mediators, such as histamine, serotonin, tryptase, are released from mast cells. As a result, allergic attacks are brought about by allergic mediators 2. In general, immune responses are tightly regulated by the immune regulatory system in the body. Regulatory T cells and regulatory B cells are the major immune regulatory cells. By releasing immune regulatory mediators, such as IL-10 and transforming growth factor (TGF)-β, immune regulatory cells suppress the activities of other immune cells 3. The state of Th2 polarization in allergic individuals reflects the regulatory immune system dysfunction 3, 4. The mechanism behind the malfunction of the immune regulation system is yet to be fully understood. Even though allergic diseases have been extensively investigated, therapies remain unsatisfactory.

Allergen-specific immunotherapy (SIT) is the mainstay in the treatment of allergic diseases 5. By introducing small doses of specific antigen into the body, SIT is expected to induce antigen-specific immune regulatory cells, and generate the antigen specific IgG in the body 5. These immune regulatory factors are expected to suppress the aberrant immune response in the body 6. Although the therapeutic effects of SIT have been acknowledged, the prevalence of allergic diseases has continued to increase in recent decades 7. The underlying mechanism is still not completely understood. There is therefore a need to investigate further the factors that compromise the efficacy of SIT.

We and others have found that psychological stress interferes with the functions of the immune system in the body 8, 9. Psychological stress is a mental activity in response to events that may have a negative effect on the body 10. For the purpose of reconciling stress, the nervous system produces several mediators, such as cortisol, norepinephrine, serotonin, etc., to counteract the effects of stress 10. In particular, certain stress-related hormones, such as cortisol, have immune-suppressive effects 11. The question of whether psychological stress interferes with the efficacy of SIT for allergic diseases has not been investigated. Therefore, we established a mouse model of airway allergic disorders (AAD). AAD mice were treated with SIT accompanying with or without restraint stress (Rs). We found that Rs significantly hampered the effectiveness of SIT for AAD. Rs induced the expression of membrane-associated RING-CH-1 (MARCH1), an E3 ligase, in dendritic cells (DC). MARCH1 interfered with the expression of IL-10 in DCs, compromising the effectiveness of SIT for AAD.

## Materials and methods

### Establishment of an AAD mouse model

The animal experimental protocols were approved by the Animal Ethical Committee at Shenzhen University (#SZUAE2020003). As depicted in Figure S1A in supplemental materials, mice were sensitized by subcutaneous injection with ovalbumin [OVA, a model antigen, 0.1 mg/mouse, mixed in 0.1 ml alum (5%)] on day 1 and day 7, respectively. Mice were treated with immune boosting by nasal instillations (20 µl/nostril, containing OVA 5 mg/ml) daily from day 9 to day 22. Nasal challenge by nasal instillation with a large dose of specific antigen (20 µl/nostril, containing OVA 50 mg/ml) was carried out on day 23.

### Allergen-specific immunotherapy (SIT)

One day after the completion of sensitization, AAD mice were treated with a 2-week SIT. Briefly, mice received nasal instillations (20 µl/nostril) daily for two weeks. The OVA vaccine (containing OVA alone) was allocated to 1 mg/ml (day 23, 24), 5 mg/ml (day 25, 26), 10 mg/ml (day 27, 28), 25 mg/ml (day 29, 30), and 50 mg/ml (day 31-35). The control group was treated with PBS nasal instillation.

### Treating mice with restraint stress (Rs)

A mouse was placed in a 50-ml conical tube with 12 holes (0.5 cm-diameter) in the tube to keep the air communication) for 1 h. The Rs session was carried out at 10 am daily during the period of SIT. Control AAD mice were treated with sham Rs, in which mice were placed in a box of the same size of mouse cages.

### Assessment of AAD response

Thirty minutes after the specific antigen challenge, under a general anesthesia, blood samples were collected from mice by the retroorbital bleeding approach. The mice were then sacrificed by the cervical dislocation. The trachea was exposed. A needle was inserted into the trachea, through which 1 ml saline was introduced into the lung. The lavage fluids were recovered immediately. The lavage was repeated two more times. The lavage fluids of three times were pooled and used as BALF (bronchoalveolar lavage fluids) in further experiments. The lungs were then excised, fixed with formalin overnight. Paraffin sections were prepared with the tissues for histology study.

### Preparation of AMCs (airway mononuclear cells)

Upon the sacrifice, the lungs were excised, and cut into small pieces. The tissues were incubated with collagenase IV (0.5 mg/ml, Sigma Aldrich) for 20 min at 37 °C with mild agitation. Single cells were filtered through a cell strainer (70 µm first, then 40 µm), followed by centrifugation (1,000 *g*, 5 min). Cell pellets were resuspended in PBS. AMCs were isolated from single cells with the Percoll gradient density centrifugation.

### Isolation of DCs from AMCs

DCs were isolated from AMCs by magnetic cell sorting (MACS) with a commercial reagent kit (Miltenyi) following the manufacturer's instruction. Isolated DCs were checked by flow cytometry. If the purity did not reach or over 95%, MACS was repeated.

### Preparation of aluminum hydroxide adjuvant (Alum)

Aluminum sulfate (Sigma Aldrich, 250 ml, 5%) was mixed with 100 ml of 5% sodium hydroxide (Sigma Aldrich) under strong stirring. The mixture was centrifuged at 3,000 *g* for 5 min. The precipitates were resuspended in normal saline, and centrifuged at 3,000 *g* for 5 min. The precipitates were resuspended in normal saline to make up to 250 ml.

### Statistics

Each experimental group consisted of 6 mice. Samples for ELISA and RT-qPCR were tested in triplicate. The difference between two groups was determined by Student's *t* test. Multiple comparisons were carried out with ANOVA followed by Dunnett's test or Bonferroni test for those more than two groups. The correlation between two groups was determined by the Pearson correlation coefficient assay. P<0.05 was set as a significant criterion.

Some experimental procedures are presented in the online supplemental materials.

## Results

### Restraint stress (Rs) hampers efficacy of SIT in mice with airway allergic disorders (AAD)

**Figure 1:**
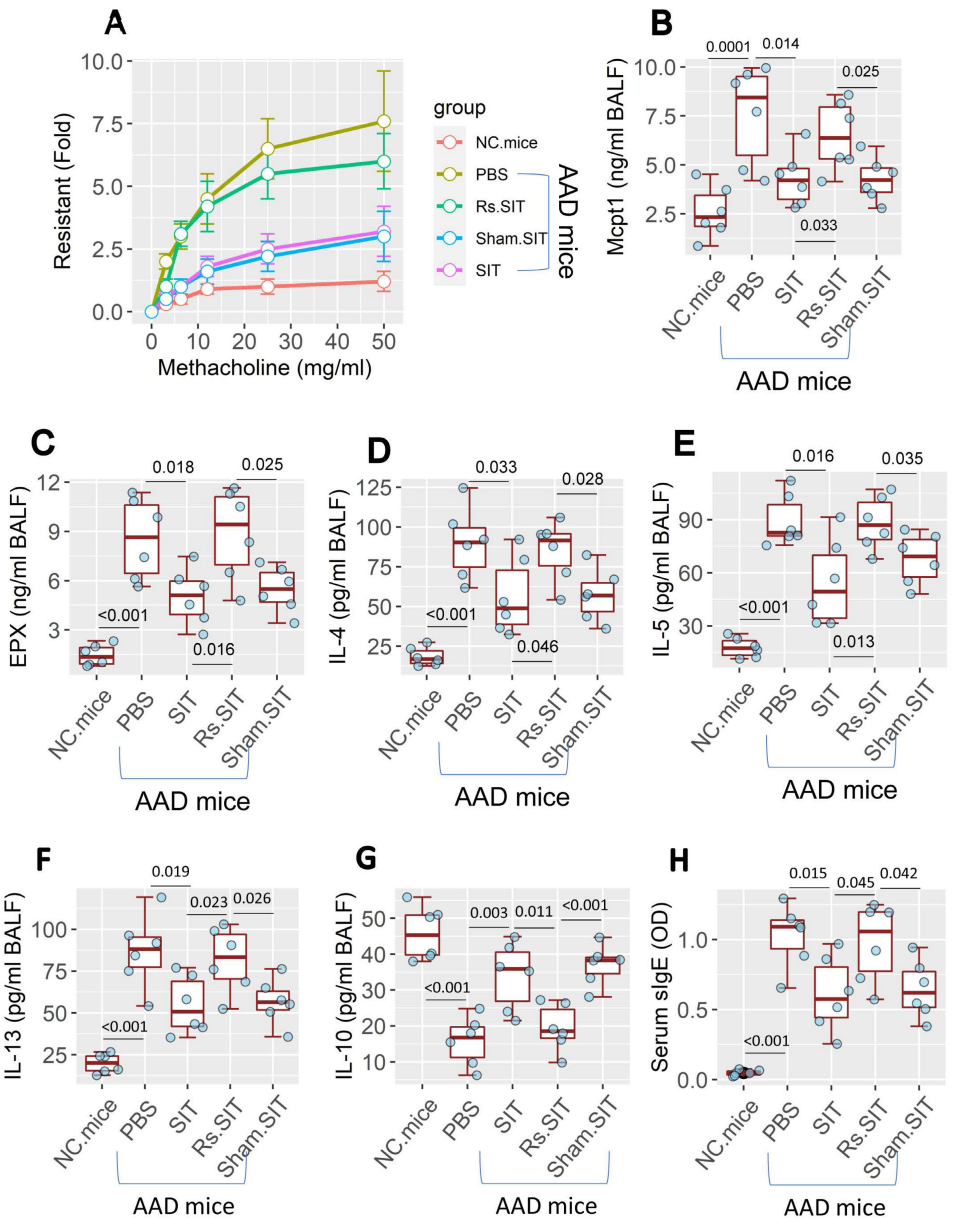
** Rs affects the efficacy of SIT**. An AAD mouse model was developed. AAD mice were treated with the procedures denoted on the X axis. A, mouse airway resistance measurement. B-C, allergic mediator levels in NLF. D-G, Levels of Th2 cytokine and IL-10 in NLF. H, serum sIgE levels. The data of boxplots are median (IQR) from 6 mice per group. Statistics: ANOVA + Bonferroni test. p values are presented in boxplots. **Abbreviations**: Rs: Restraint stress. AAD: Airway allergic disorder. SIT: Specific allergen immunotherapy. Sham: Sham stress. NLF: Nasal lavage fluid. IQR: InterQuartile range. sIgE: Specific IgE. PBS: Phosphate-buffered saline.

An AAD mouse model was established (Figure S1A in supplemental materials). Mice showed the AAD response 12, including airway hyper responsiveness (Figure 1A), higher levels of mast cell protease-1 (Mcpt1), eosinophil peroxidase (EPX) and Th2 cytokines (Figure 1B-F) in bronchoalveolar lavage fluids (BALF). Lower BALF IL-10 levels (Figure 1G) and higher serum specific IgE (sIgE) (Figure 1H) were also observed in AAD mice. Treatment of AAD mice by SIT mitigated the AAD response. However, the treatment of AAD mice with SIT and Rs (Figure S1B) showed a much weaker therapeutic effect on AAD response than those treated with SIT alone (Figure 1). The results demonstrate that Rs undermines the therapeutic efficacy of SIT.

### Rs alters the immune regulation capacity of AAD mice during SIT

**Figure 2:**
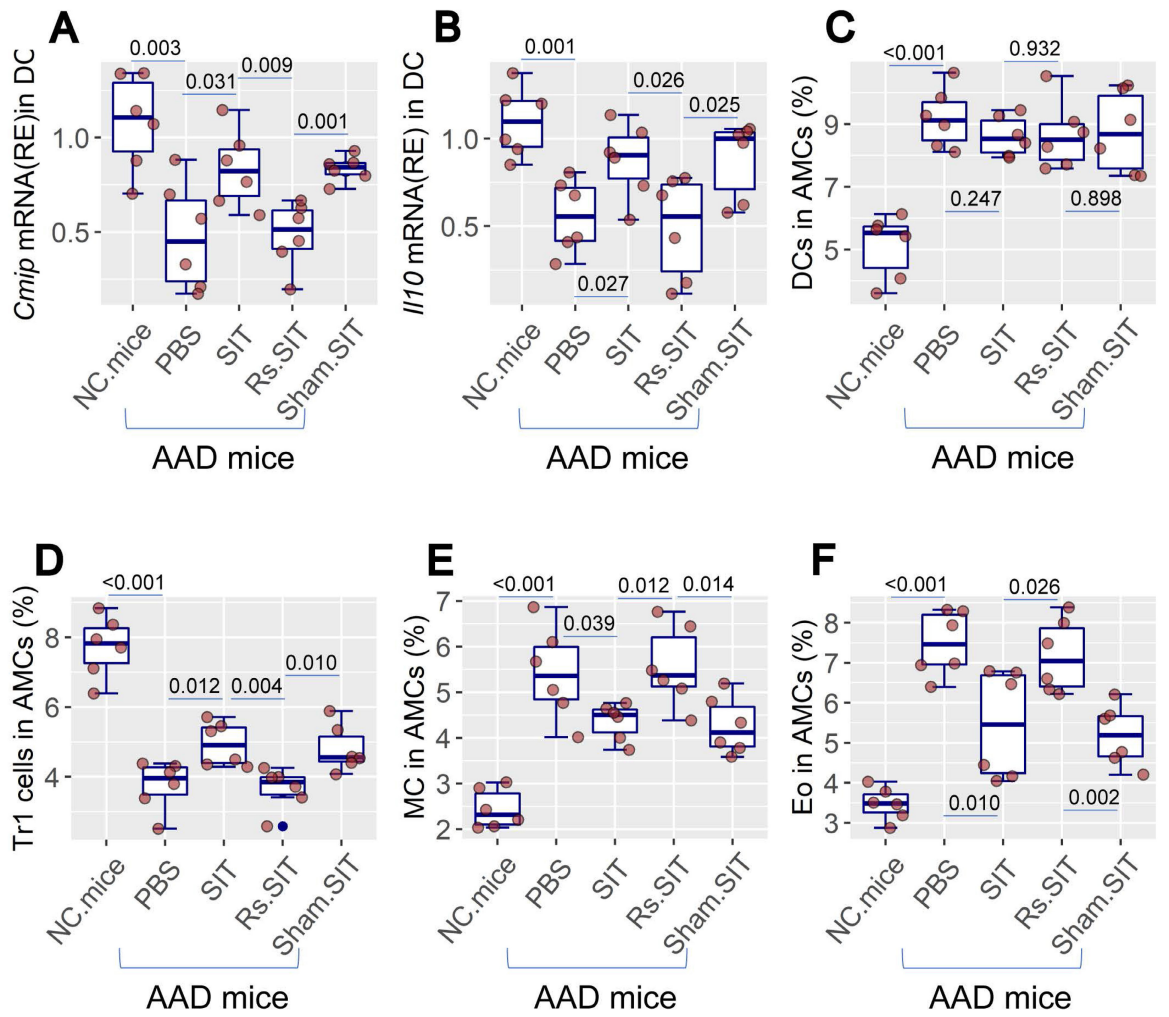
** Rs suppresses immune regulatory activities in the airway tissues**. AMCs were prepared from NC mice and AAD mice after the treatment denoted on the X axis. A-B, CD11c^+^ DCs were purified from AMCs. RNA extracts of AMCs were analyzed by RT-qPCR. Boxplots show the mRNA levels of *Cmip* and *Il10* in DCs. C-F, AMCs were analyzed by FCM. Boxplots show the frequency of DC, Tr1 cells, mast cells and eosinophils in AMCs (the FCM plots are presented in Fig. S2-5 in supplemental materials). The data of boxplots are median (IQR) of 6 samples per group. Statistics: ANOVA + Bonferroni test. p value is presented in boxplots. **Abbreviations**: AMC: Airway mononuclear cells. NC: Naïve control. AAD: Airway allergy disorder. FCM: Flow cytometry. Tr1 cell: Type 1 regulatory T cell. MC: Mast cell. Eo: Eosinophil. IQR: InterQuartile range.

Previous studies indicate that psychological stress affects the activities of the immune system 8, 9. As such, it is important to elucidate the mechanism by which Rs affects the therapeutic efficacy of SIT. We then assessed immune activities in mice treated with SIT or/and Rs. The DC number was not apparently altered by Rs in the AAD mouse airway tissues. However, the c-Maf-inducing protein (CMIP) as well as IL-10 mRNA levels were reduced in DCs by Rs (Figure 2A-C, Figure S2). The frequency of type 1 regulatory T cell (Tr1 cell) was reduced in airway tissues by Rs (Figure 2D, Figure S3). The number of mast cells and eosinophils in the airway tissues of AAD mice was increased by Rs (Figure 2E-F, Figure S4, S5). The results indicate that Rs suppresses the immune regulatory functions in the airway tissues.

### The Rs-induced cortisol release is associated with the AAD response

**Figure 3:**
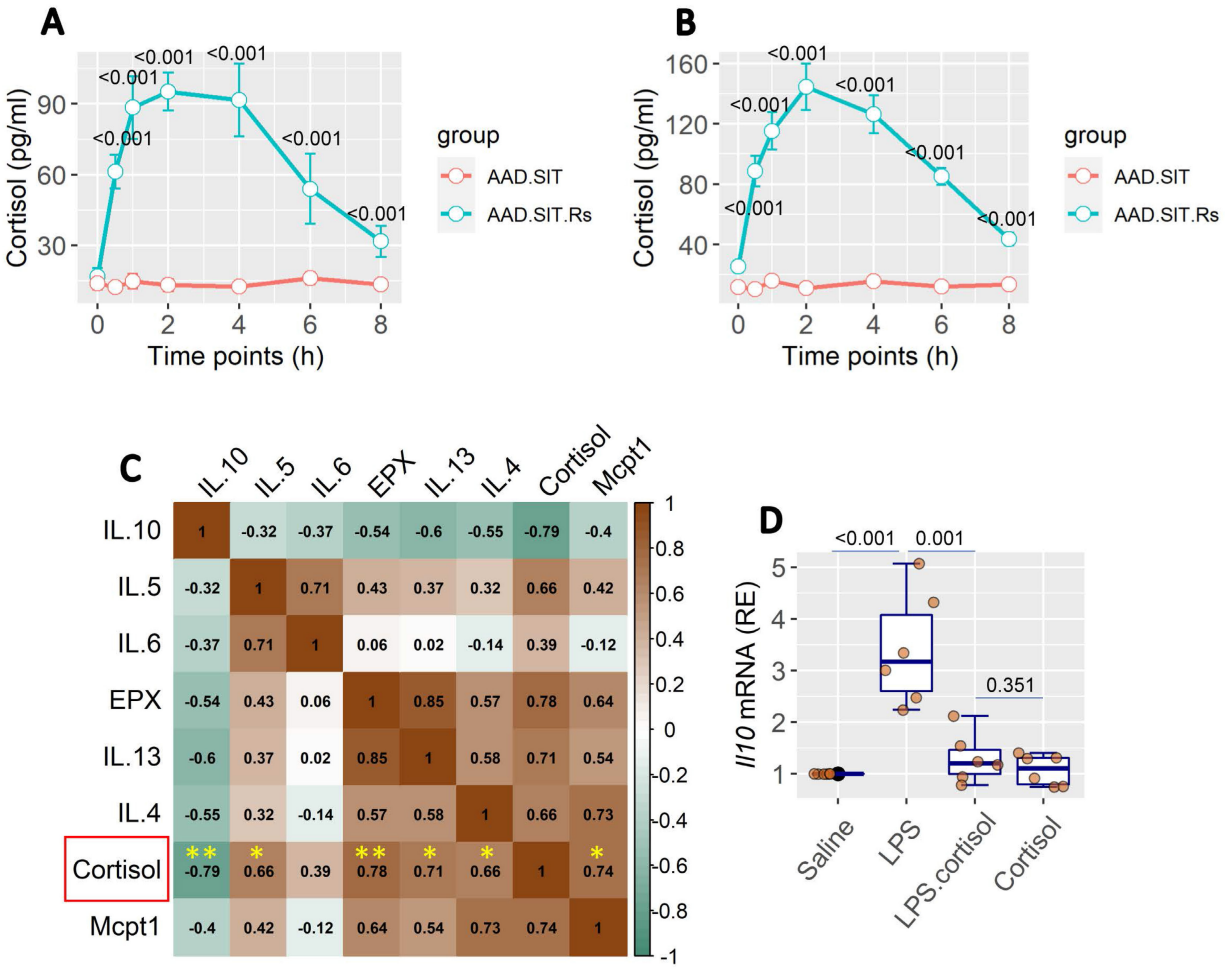
** Assessment of BALF cortisol levels and its relation with the AAD response**. A, BALF and serum was prepared with samples collected from SIT-treated and Rs.SIT-treated AAD mice (6 per group) at indicated time points. The curve shows the levels of cortisol in the serum and BALF. B, BALF samples were collected from Rs.SIT-treated AAD mice after completing the therapy. C, the heatmap shows correlation between the cortisol levels and the levels of AAD response-related cytokines in BALF. The numbers in heatmap show correlation coefficient. D, boxplots show the *Il10* mRNA levels in BMDCs after exposure to LPS (100 ng/ml) in culture overnight. Cortisol: the presence of cortisol in culture (20 ng/ml). **Abbreviations**: BALF: Bronchoalveolar lavage fluid. AAD: Airway allergic disorder. Rs: Restraint stress. SIT: Specific allergen immunotherapy. AAD.SIT: SIT-treated AAD mice. AAD.SIT.Rs: SIT and Rs treated AAD mice. BMDC: Bone marrow derived dendritic cell. LPS: Lipopolysaccharide.

Published data show that cortisol plays an important role in stress-related activities in the body 13, 14. We also found an increase in cortisol levels in the serum and BALF. Cortisol levels remained high up to 6 h after Rs (Figure 3A-B). It indicates that Rs induces the release of cortisol, which occurs in both the serum and lungs. BALF collected from AAD mice after the Rs.SIT therapy was processed and analyzed. The results showed a positive correlation in the data between the cortisol levels and the levels of AAD-related cytokine levels, including Mcpt1, EPX, and Th2 cytokines. A negative correlation was detected between cortisol and IL-10 levels in the BALF (Figure 3C). The results imply that cortisol may influence the gene activity in local immune cells such as DCs. To this end, bone marrow-derived DCs (BMDCs) were stimulated with lipopolysaccharide (LPS) in culture to increase the IL-10 expression. The presence of cortisol in culture was effective in suppressing the IL-10 expression in BMDCs (Figure 3D).

**Figure 4:**
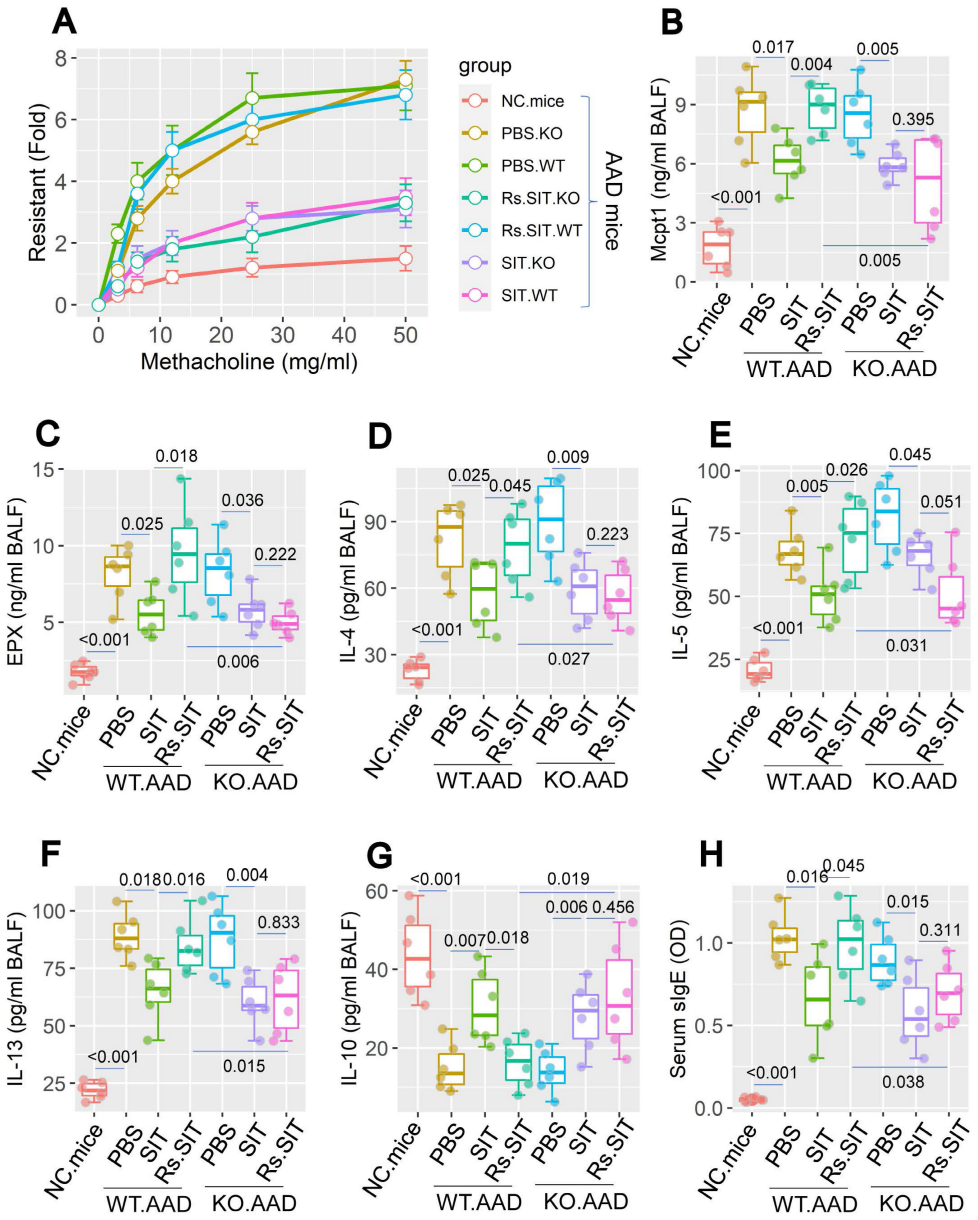
** Assessment of the role of cortisol-GR axis in mediating Rs effect on compromising SIT**. WT mice and *GR*^∆DC^ mice were sensitized by using the OVA/Alum protocol with or without treating with Rs. A, airway resistant records in response to methacholine challenge. B-G, levels of Mcpt1, EPX, Th2 cytokines and IL-10 in BALF. H, serum levels of specific IgE (sIgE). Statistics: ANOVA + Bonferroni test. p values are presented in boxplots. **Abbreviations**: Rs: Mice were treated with restraint stress. NC: Naïve control. WT: Wild type. SIT: Specific allergen immunotherapy. KO: *GR*^∆DC^ mice. GR: Glucocorticoid receptor. OVA: Ovalbumin. EPX: Eosinophil peroxidase.

The data of Figure 3 imply that the Rs-induced cortisol release affects DC's immune regulatory property in the lungs. As a result, it may expand the existing Th2 polarization in the local tissues. To verify this, we constructed the *GR*^∆DC^ mice; a mouse strain carries glucocorticoid receptor (GR) deficient DCs. DCs from *GR*^∆DC^ mice exhibited the normal IL-10 induction in response to LPS in culture (Figure S6). Wild type (WT) mice and *GR*^∆DC^ mice were treated with the OVA/Alum protocol (Figure S1). Both WT mice and *GR*^∆DC^ mice demonstrated the AAD response. Treatment with Rs affected the therapeutic efficacy of SIT in WT AAD mice, but did not affect SIT in *GR*^∆DC^ AAD mice (Figure 4). In addition, AAD mice treated with sham Rs did not affect the SIT efficacy on AAD (not shown). The anti-inflammatory cytokine, IL-10, was detectable in BALF. Lower concentrations of BALF IL-10 were found in AAD mice (including *GR*^∆DC^ AAD mice), which were significantly increased by SIT. AAD mice treated with both SIT and Rs did not show the elevation of IL-10 in BALF. Genetic ablation of GR in DCs abolished the impact of Rs on recovering IL-10 levels in BALF (Figure 4). The results confirmed the role of the cortisol-GR axis in mediating the impact of Rs on SIT.

### Rs modulates expression of MARCH1 and CMIP in DCs

**Figure 5:**
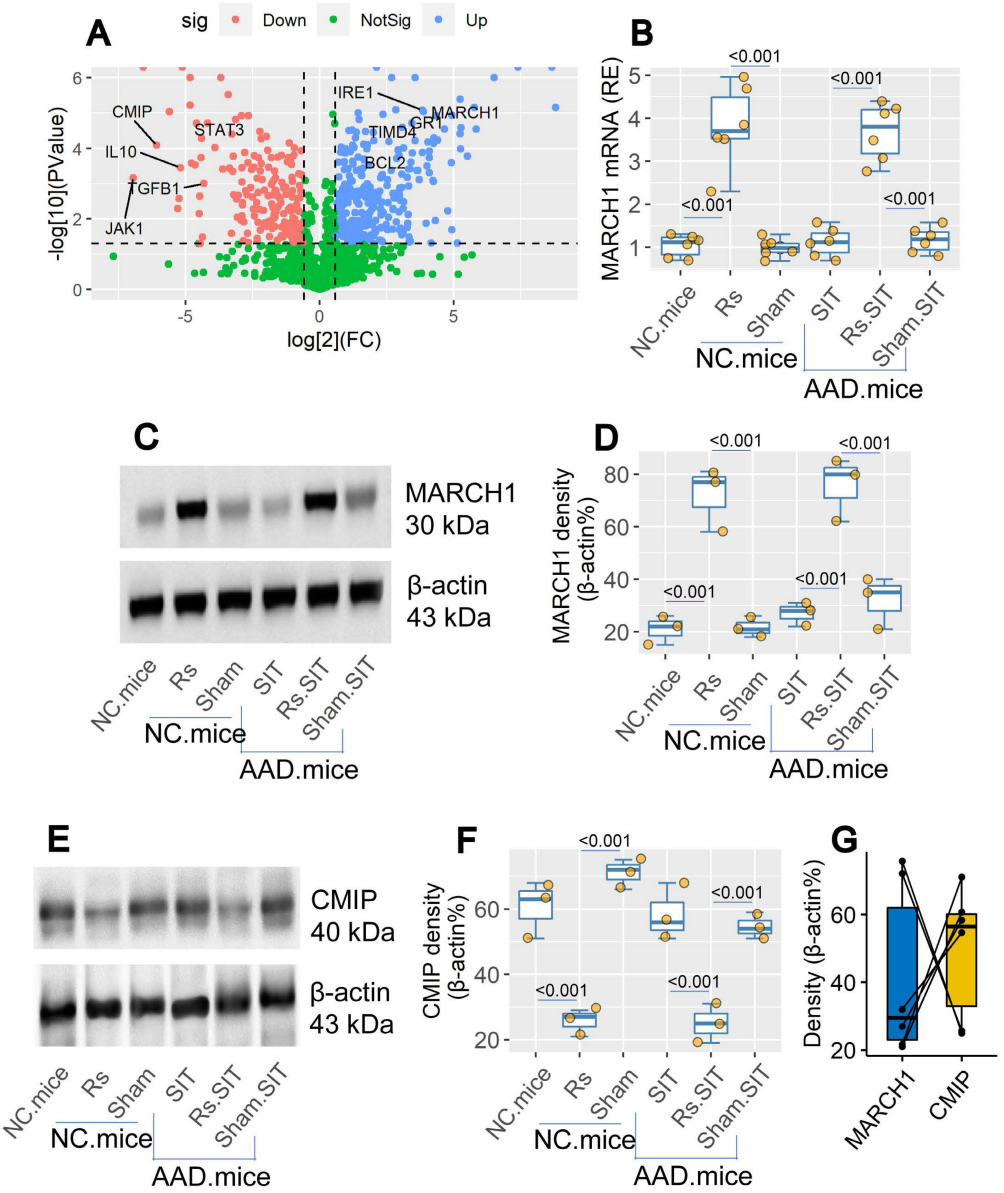
** Rs modulates the levels of MARCH1 and CMIP in DCs**. Naïve mice and AAD mice (n = 6 per group) were treated with Rs or sham Rs (sham, in short) for two weeks. DCs were isolated from the airway tissues. A, volcano plots show relevant DEGs in DCs. B, the levels of MARCH1 mRNA in DCs. C, immunoblots show MARCH1 protein in DCs. D, boxplots show the integrated density of the immunoblots of MARCH1. E, immunoblots show CMIP levels in DCs. F, boxplots show integrated density of CMIP blots in panel E. G, paired point plots show correlation between MARCH1 and CMIP blot density. **Abbreviations**: Rs: Restraint stress. DC: Dendritic cell. Rs: Restraint stress. DEG: Differentially expressed gene. CMIP: c-Maf inducing protein. NC: Naïve control mice. Density: Integrated density of immunoblot.

By bulk RNA sequencing (RNAseq), we found the elevated *March1* gene activity in DCs of the airway tissues after treating mice with Rs (Figure 5A). The data were verified by conventional RT-qPCR and Western blotting (Figure 5B-D). The expression of MARCH1 in DCs was also up regulated in airway DCs of AAD mice treated with Rs (Figure 5B-D). In Western blotting assay, we found that c-Maf (the transcription factor of IL-10) inducing protein (CMIP) levels were down regulated by Rs in DCs of both naïve mice and AAD mice (Figure 5E-F). The levels of MARCH1 and CMIP in the DCs showed a negative correlation trend (Figure 5G). The results indicate that Rs can up regulate the expression of MARCH1 in DCs. MARCH1 may be involved in the regulating CMIP in DCs.

### Targeting MARCH1 prevents Rs-induced SIT incompetence

The expression of MARCH1 in DCs plays an important role in Th2 polarization 15, and can be up regulated by stressful events 16. Thus, based on the results in Figure 5, we examined the role of MARCH1 in compromising the immune therapeutic efficacy of SIT. A mouse strain carrying *MARCH1*-deficient DCs (*MARCH1*^f/f^*Itgax*-Cre mice; *MARCH1*^ΔDC^ mice, in short) was constructed. Ablation of the MARCH1 expression in DCs did not apparently affect the number of DC, T cell, B cell, eosinophil, mast cell, and Tr1 cell in the airway tissues (Figure S7). Rs reduced the IL-10 expression in DCs. MARCH1 is an E3 ligase, which can induce ubiquitination in targeted proteins to induce the protein degradation 17. Thus, immunoprecipitation (IP) was performed with protein extracts of DC using an anti-MARCH1 Ab as a bait. A complex of MARCH1 and CMIP (Figure S8A) was detected in the protein samples extracted from the airway DCs of mice treated with the Rs.SIT protocol (Figure S1). Notably, ubiquitin was colocalized with CMIP in the same PVDF membrane using the stripping-re-staining method (Figure S8B). The data provide evidence that MARCH1 induces CMIP degradation through ubiquitination. This was confirmed by the specific staining for ubiquitination (Figure S8C). The results indicate that Rs impairs the immune regulatory properties of DC by interfering with the expression of IL-10. To substantiate the results, MARCH1 and CMIP plasmids were transfected to HEK293 cells. A complex of MARCH1 and CMIP was generated in HEK293 cells, where ubiquitin was co-localized with CMIP (Figure S8B-C).

**Figure 6:**
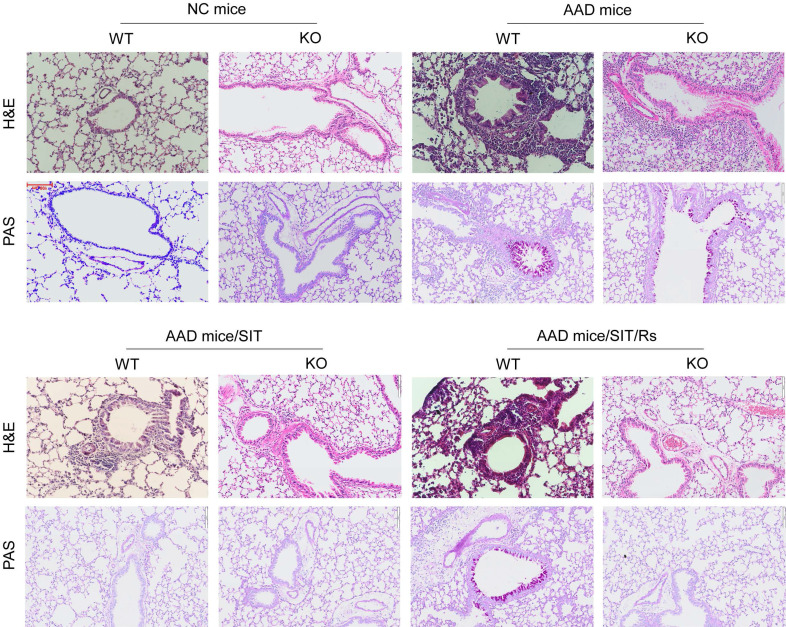
** Blocking MARCH1 in DCs prevents the Rs-compromised SIT effects on lung inflammation**. WT mice and *MARCH1*^∆DC^ mice were treated with the OVA/Alum protocol to develop an AAD mouse model. Mice were treated with procedures denoted above each image. Histology images of the lung show the results of H&E staining and PAS staining (original magnification: ×100). The images are from one mouse/group that represent 6 mice per group. **Abbreviations**: DC: Dendritic cell. Rs: Restraint stress. SIT: Specific allergen immunotherapy. OVA: Ovalbumin. AAD: Airway allergic disorder. H&E: Hematoxylin and eosin staining. PAS: Periodic acid-Schiff staining. WT: Wild type mice. KO: *MARCH1*^∆DC^ mice (mice carrying *MARCH1*-deficient DCs).

**Figure 7:**
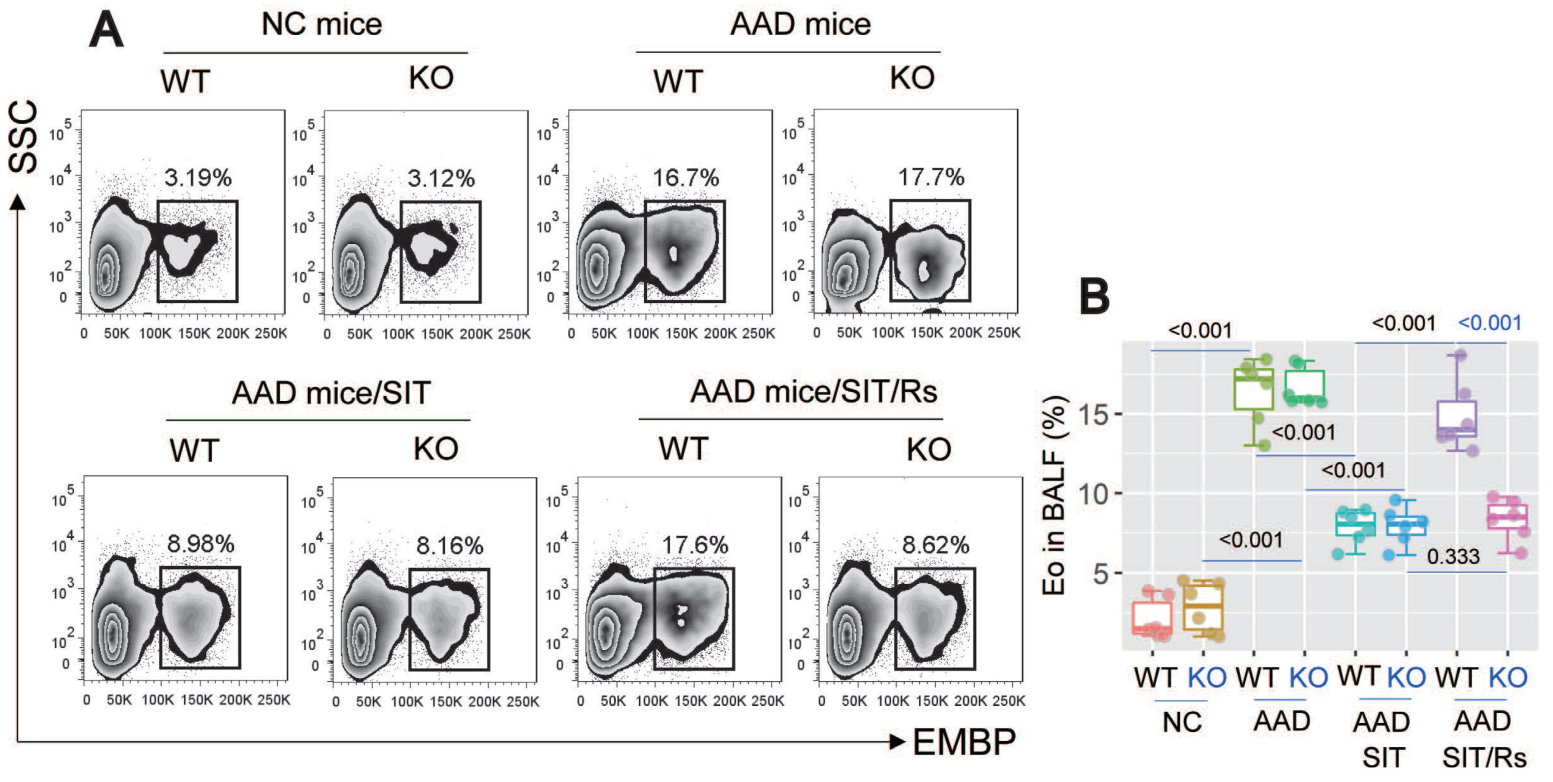
**Blocking MARCH1 in DCs prevents the Rs-compromised SIT effects on regulating eosinophil (Eo) infiltration of the lung**. AMCs were prepared with the lung tissues collected from mice described in Fig. 6. A, gated FCM histograms show eosinophil counts in BALF. B, boxplots show median (IQR) of eosinophil counts of 6 mice per group. Statistics: ANOVA + Bonferroni test. P values are presented in boxplots. **Abbreviations**: DC: Dendritic cell. Rs: Restraint stress. SIT: Specific allergen immunotherapy. OVA: Ovalbumin. AAD: Airway allergic disorder. FCM: Flow cytometry. WT: Wild type mice. KO: *MARCH1*^∆DC^ mice (mice carrying *MARCH1*-deficient DCs). AMC: Airway mononuclear cell. EMBP: Eosinophil major basic protein.

**Figure 8:**
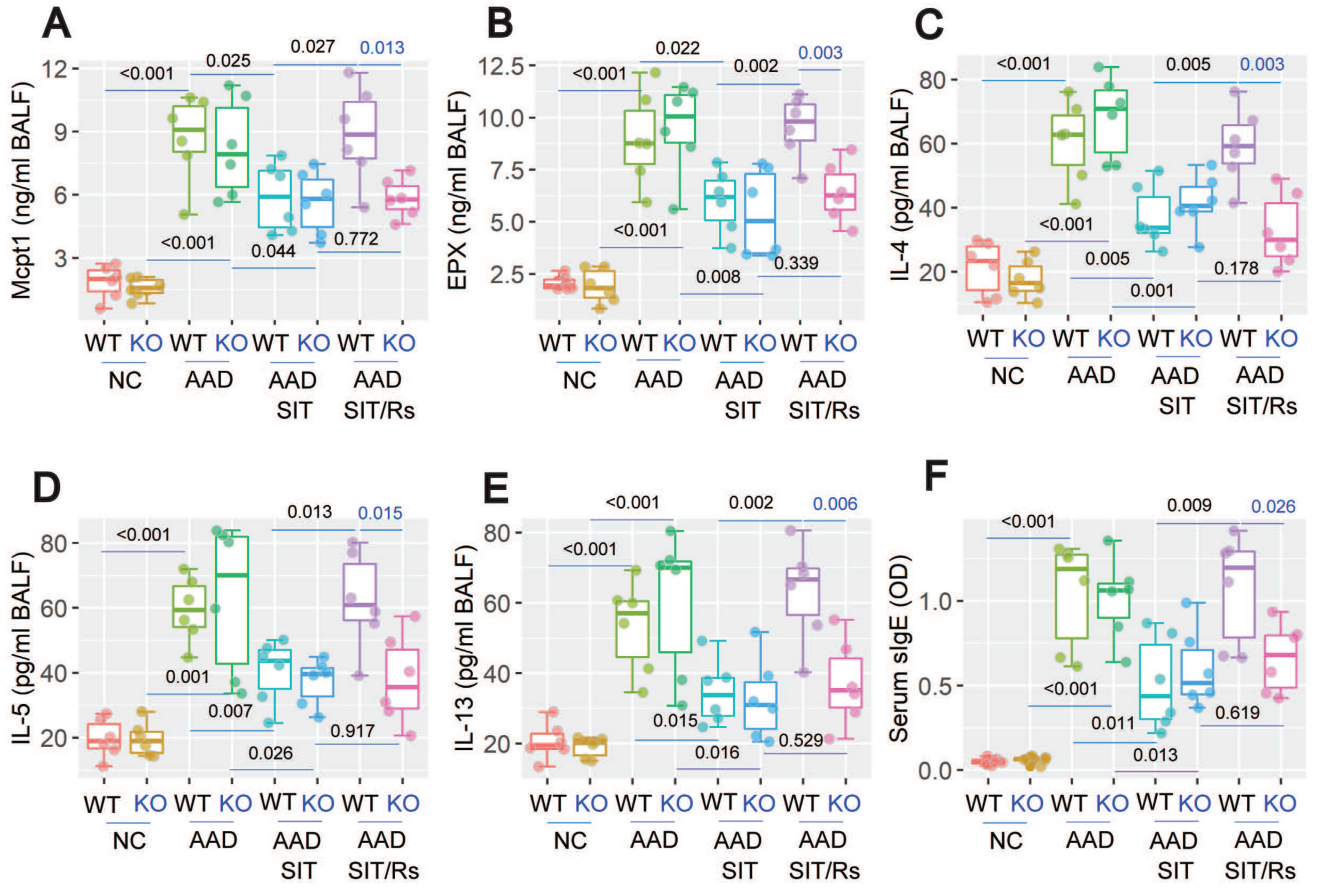
Blocking MARCH1 in DCs prevents the Rs-compromised SIT effects on regulating serum sIgE and proinflammatory cytokine in BALF. Serum and BALF were collected from mice described in Fig. 6. A-E, median (IQR) of the major proinflammatory cytokines in BALF. F, serum sIgE levels. Each group consists of 6 mice. **Abbreviations:** DC: Dendritic cell. Rs: Restraint stress. SIT: Specific allergen immunotherapy. OVA: Ovalbumin. AAD: Airway allergic disorder. WT: Wild type mice. KO: *MARCH1*^∆DC^ mice (mice carrying *MARCH1*-deficient DCs). sIgE: Specific IgE.

The above data suggest that MARCH1 may be a target for reconciling the compromised efficacy of SIT by Rs. For this purpose, an AAD mouse model was established using WT mice and *MARCH1*^∆DC^ mice with the OVA/Alum protocol (Figure S1). The AAD response was induced in both WT mice and *MARCH1*^∆DC^ mice. The lung tissues showed profound inflammatory cell infiltration, increase in thickness of the small airways, and increase in mucus production (Figure 6). BALF was collected from mice, in which the number of eosinophil (Figure 7) and AAD-associated pro-inflammatory cytokines were elevated in AAD mice (Figure 8A-E). The serum sIgE levels were higher in AAD mice than that in control mice (Figure 8F). AAD mice were then treated with SIT or Rs/SIT (Figure S1). We observed that SIT markedly attenuated the AAD response. However, Rs significantly hampered the SIT effects in WT AAD mice, but not in *MARCH1*^∆DC^ AAD mice (Figure 6-8). The results indicate that the Rs-induced MARCH1 in DCs makes a substantial negative impact on SIT efficacy.

## Discussion

This study revealed a new phenomenon whereby Rs affects the effectiveness of SIT for AAD. Rs significantly impaired the therapeutic effects of SIT for experimental AAD. The immunoregulatory activities of DC were hampered by Rs. Rs raised cortisol levels in the serum, which is an important psychological stress hormone 14. Through the effects of cortisol, Rs induced the MARCH1 expression in DCs. MARCH1 mediated the effects of Rs on suppressing the efficacy of SIT. Inhibition of MARCH1 effectively blocked the effects of Rs on compromising SIT efficacy for experimental AAD.

As aforementioned, SIT is the mainstay of the treatment of allergic diseases 18. Based on immunological principles, constant exposure to low doses of specific antigen can induce antigen-specific immune tolerance 5. SIT is such a therapeutic tool for allergic illnesses. The therapeutic efficacy of SIT has been recognized in numerous studies 5. However, the increasing prevalence of allergic diseases around the world 19 means that the therapeutic effectiveness of SIT can be further improved. The current study revealed that psychological stress is an important factor in compromising the effects of SIT on the mitigation of experimental AAD. Psychological stress events happen all the time in people's everyday lives 20. Humans can generally handle most stress events appropriately. However, if not coping well, psychological stress can cause a variety of body injuries 20. One of the impacts of psychological stress on the body is to affect the immune tolerant system. For instance, stress can lead to the production of IL-6 21. IL-6 works synergistically with TGF-β to convert Tregs into Th17 cells 22, and hence, affects immune tolerance. The presence data show that during SIT, Rs induces the MARCH1 expression in DCs to interfere with the expression of IL-10. This further prevents the Tr1 cell induction, indicating that Rs can prevent the immune tolerance induction during SIT.

In the events of psychological stress, the stress hormones are released from the nervous system. These hormones are also known as stress mediators, like cortisol, norepinephrine, serotonin, etc. 23. Current data demonstrate that cortisol is the canonical stress mediator in Rs. Cortisol also acts as an immunosuppressant. We observed that Rs significantly inhibited the signals of the IL-10 expression pathway in DCs. IL-10 is the chief player of the tolerogenic properties in DCs 24. The insufficient expression of IL-10 caused by Rs generates a substantial impact on the SIT-induced tolerogenic feature in DCs. In this way, Rs compromises the therapeutic efficacy of SIT for AAD.

Thus, to elucidate the mechanism by which Rs affects the expression of IL-10 is the point of reconciling the suppressing effects on immune regulation functions. Based on the indication that Rs increases cortisol release, we found that cortisol was associated with the increased MARCH1 expression in DCs of the mice exposed to Rs. MARCH1 is an E3 ligase and can help target protein breakdown 17. Recent studies have also shown that MARCH1 interferes with immune regulatory functions, particularly by favoring Th2 polarization 15. MARCH1 expression can disrupt DC homeostasis and disturb immune regulatory activities 15. The current data are in line with these latest findings by showing that MARCH1 plays a canonical role in the suppression of IL-10 expression in the DCs. The results suggest that, presumably, MARCH1 is the target to reconcile the Rs effects on compromising the efficacy of SIT.

The data show that blocking MARCH1 reconciles the impact of Rs on the therapeutic efficacy of SIT. As noted above, the efficiency of SIT needs to be improved 5, 19. Current findings suggest that Rs is an important factor that interfere with the therapeutic effectiveness of SIT. Rs induces DCs to express MARCH1. MARCH1, in turn, suppresses the expression of IL-10, and prevents the induction of tolerogenic DCs and Tr1 cells. Therefore, the findings suggest that MARCH1 is a new target to improve the efficiency of SIT. There is a need to develop MARCH1 inhibitors to be used in blocking disorders involved in the Rs-related immune dysfunction.

The present data demonstrated the critical role of MARCH1 in mediating the effects of Rs on impairing the regulatory effects of DC during SIT. However, there are several psychological stress-related hormones have been noted. Previous research has shown that corticotrophin releasing hormone plays an important role in mediating the effects of psychological stress on weakening epithelial barrier functions 25, 26. Furthermore, many stress hormones, such as norepinephrine, pro-opiomelanocortin peptides and arginine vasopressin, also affect immune activities 23. The present study does not exclude these hormones from the Rs-related effects on immune cell activities. This needs to be further investigated.

The best conclusion of the current study is that Rs modulates the expression of MARCH1 and IL-10 to DC during the SIT. SIT has noticeable effects on alleviating allergic response, which is mainly attributed to the induction of the IL-10-producing tolerogenic DCs 27. The IL-10-producing DCs are capable of inducing type 1 regulatory T cells to produce the immune regulatory activities 28. Psychological stress can alter immune cell activity in subjects with allergic disorders 29, which may affect the efficacy of SIT. The present data provide evidence that Rs-induced MARCH1 expression compromises the production of IL-10 in DCs. Consequently, Rs hampers the efficacy of SIT.

In summary, current data indicate that Rs is a critical factor affecting the therapeutic efficacy of SIT. By promoting the release of cortisol, Rs induces immune suppression in the airway tissues. The production of immune regulatory cytokine, IL-10, is thus suppressed in DCs. Consequently, Rs prevents the generation of immune tolerance in the tissues of the airways. This undermines the effectiveness of the SIT. Blocking MARCH1 can reconcile the Rs effects to promote immunotherapy against airway allergy.

## Supplementary Material

Supplementary materials and methods, figures and table.
