# Supplementary material for: Targeting psychological stress-steroid-MARCH1 signaling pathway promotes the efficacy of specific allergen immunotherapy
Source: Theranostics. 2022 Nov 8;12(18):7717–28. doi: 10.7150/thno.78851 (PMC9706592; doi:10.7150/thno.78851)
Supplement: Supplementary file 1 — Supplementary materials and methods, figures and table. [file thnov12p7717s1.pdf]

Supplemental materials

Reagents

ELISA kits of Mcpt1, EPX, IL-4, IL-5, IL-13, OVA-specific IgE were purchased from CRK Pharma (Wuhan, China). Cortisol ELISA kit was purchased from Mingjing Biotech (Shanghai, China). CD11c (D-8, AF700), IL-10 (2G101H7, AF488), CD3 (PC3/188A, AF488), CD4 (MT310, AF594), CD19 (B-1, AF790), LAG3 (C9B7W), CD49b (C-9, AF546), EMBP (F-6, AF648), His (H-3) were purchased from Santa Cruz Biotech (Santa Cruz Biotech (Santa Cruz, CA). MARCH1 Ab was purchased from Biocompare (South San Francisco, CA). Ovalbumin (purity ≥ 97%; EndoGrade®, salt free endotoxin level: < 0.1 EU/mg) was purchased from AmyJet Scientific (Wuhan, China). Flag Ab was purchased from Sigma Aldrich (St. Louis., MO). Mcpt1 Ab (APC) was purchased from BD Biosciences (Franklin Lakes, NJ). CMIP Ab, reagents and materials for RT-qPCR and Western blotting were purchased from Invitrogen (Carlsbad, CA).

Mice

BALB/c mice (6-8-week-old) and C57/B6 mice were purchased from the Guangdong Experimental Animal Center (Guangzhou, China). *Itgax*-Cre mice were purchased from Jackson Laboratory (Bar Harbor, ME). Mice were maintained in a specific pathogen-free facility at Shenzhen University. Mice were allowed to access food and water freely. Following the published strategies (*Bettigole SE, et al. The transcription factor XBP1 is selectively required for eosinophil differentiation. Nat Immunol 2015; 16:829-37. Yoshinobu K, et al. Tamoxifen feeding method is suitable for efficient conditional knockout. Exp Anim 2021; 70:91-100*), *MARCH1*$^{f/f}$*Itgax*-Cre mice (*MARCH1*$^{\Delta DC}$ mice, in short), of which the *MARCH1* gene was deleted in DCs, expressing Cre recombinase from the *Itgax* promoter (*Itgax*-Cre mice), were crossed with *MARCH1*$^{f/f}$ mice (mice with loxP-flanked *MARCH1* alleles). In similar strategy, *GR*$^{\Delta DC}$ mice (mice carrying *GR* gene-deficient DCs by crossing *Itgax*-Cre mice with loxp-flanked *GR* allele, *GR*$^{f/f}$, mice), were developed. To start the ablation of *MARCH1* gene or *GR* gene, *MARCH1*$^{\Delta DC}$ mice or *GR*$^{\Delta DC}$ mice were gavage-fed with tamoxifen (Sigma Aldrich, 200 mg/kg in corn oil) daily for 5 consecutive days prior to experiments. DCs in the airway tissues of *MARCH1*$^{\Delta DC}$ mice or *GR*$^{\Delta DC}$ mice did not show detectable *MARCH1* or *GR* expression. The frequency of DC in the airway tissues of *MARCH1*$^{\Delta DC}$ mice or *GR*$^{\Delta DC}$ mice was not significantly different from that of wild type (WT) mice.

Assessment of airway resistance

Airway resistance was assessed in mice on day 23. Mice were anesthetized with chloral hydrate (400 mg/kg, i.p). Tracheal intubation was performed, mice were fixed in a closed body drawing box and mechanically ventilated. Mice were nebulized with methacholine at 0.1 ml containing gradient concentrations of methacholine (0, 6.25, 12.5, 25, and 50 µg/ml). Data from 5 seconds to 1-minute after inhalation were acquired. The lung resistance was calculated and presented as fold change.

ELISA (Enzyme-linked immunosorbent assay)

The levels of specific IgE (sIgE) and cytokines in the serum and BALF were determined by ELISA with commercial reagent kits following the manufacturer's instructions.

Cell culture

Cells were cultured in RPMI1640 medium supplemented with fetal bovine serum (FBS, 10%), L-glutamine (2 mM), streptomycin (0.1 mg/ml), and penicillin (100 U/ml). Cell viability was greater than 99% as assessed by Trypan blue exclusion assay.

Real-time quantitative RT-PCR (RT-qPCR)

RNA was extracted from cells collected from relevant experiments, and converted to cDNA with a reverse transcription kit following the manufacturer's instructions. The cDNA samples were amplified in a qPCR device (Bio-Rad, CFX96) with the SYBR Green Master Mix in the presence of relevant primers, including *Il10* (ataactgcacccacttccca and gggcatcacttctaccaggt), *Cmip* (cctacagcgccattgaagac and ggcttgggttactcaggact), *MARCH1* (gaaagcccactcatcacacc and ctttcgctcgtggtcatctg). The results were presented as relative expression after calculating with the $2^{-\Delta\Delta Ct}$ method.

Western blotting

Proteins were extracted from cells collected from various experiments, separated by SDS-PAGE, and transferred onto a PVDF membrane. After blocking with a blocking buffer (5% skim milk), the membrane was stained with primary Abs of interest (diluted to 200 ng/ml; Ab types are detailed in figures) overnight at 4 °C, washed with TBST (Tris-buffered saline containing 0.05% Tween20), incubated with secondary Abs (diluted to 20 ng/ml; labeled with horseradish peroxidase) for 2 h at room temperature, washed with TBST 3 times. The immunoblots on membrane were developed by the enhanced chemiluminescence, and photographed in an imaging device (UVP, Cambridge, UK). The uncropped images are presented in source data 1 in supplemental materials.

Immunoprecipitation (IP)

Proteins were extracted from cells collected from relevant experiments. Pre-existing immune complexes in the samples were cleared by incubating with protein G agarose beads for 2 h to adsorb the immune complexes. The beads were removed by centrifugation (5,000 *g*). Samples were then incubated with relevant Abs (detailed in figures; diluted at 1 µg/ml) for 2 h with mild agitation, followed by incubating with protein G agarose beads. The beads were collected by centrifugation (5,000 *g*) for 5 min. Proteins on the beads were eluted, and analyzed by Western blotting. On the other hand, the membrane stained with CMIP Ab was treated with a peeling buffer to remove the CMIP Ab. The membrane was then re-stained with a ubiquitin Ab.

Flow cytometry (FCM)

Cells were collected from relevant experiments. In the surface staining, cells were stained with Abs (labeled with fluorescence, detailed in figures) of interest or isotype IgG for 30 min at 4 °C. After washing with PBS, cells were analyzed with a flow cytometer (BD FACSCanto II). In the intracellular staining, cells were fixed in 1% paraformaldehyde (containing 0.05% Triton X-100 to increase the membrane

permeability) for 1 h. Cells were washed with PBS, and processed with the same procedures of the surface staining. The data were analyzed with a software package (Flowjo, TreeStar Inc., Ashland, OR) with the data obtained from isotype IgG staining as gating references.

Assessment of MARCH1 binding CMIP in HEK293 cells

A plasmid of MARCH1-expression and a plasmid of CMIP-expression were constructed by Sangong Biotech (Shanghai, China). The plasmid design is illustrated in Fig. S8 in supplemental materials. HEK293 cells were transfected with the plasmids following the manufacturer's instruction. Forty-eight hours later, the cells were harvested. Proteins were extracted from the cells, and analyzed by IP.

RNA-sequencing (RNAseq)

DCs were isolated from AMCs by MACS. Total RNA was extracted from DCs using the TRIzol reagents following the manufacturer's instruction. RNA samples were analyzed by RNAseq by professional staff in a biotech company (BGI, Shenzhen, China). Briefly, the library was constructed, followed by analyzing with an Illumina platform (HiSeq 2500, Illumina). Gene expression was assessed using the DESeq R package (version 1.18.0). Multiple adjustment tests were performed to calculate the adjusted *P*-value (adjpval). Genes with corrected P values of less than 0.05 and log2 (fold change, FC) of 1 or greater between two groups were considered to have significantly differential expressions. The differentially expressed genes (DEGs) were analyzed by ontology analysis. The raw data are presented in source data 2 in supplemental materials.

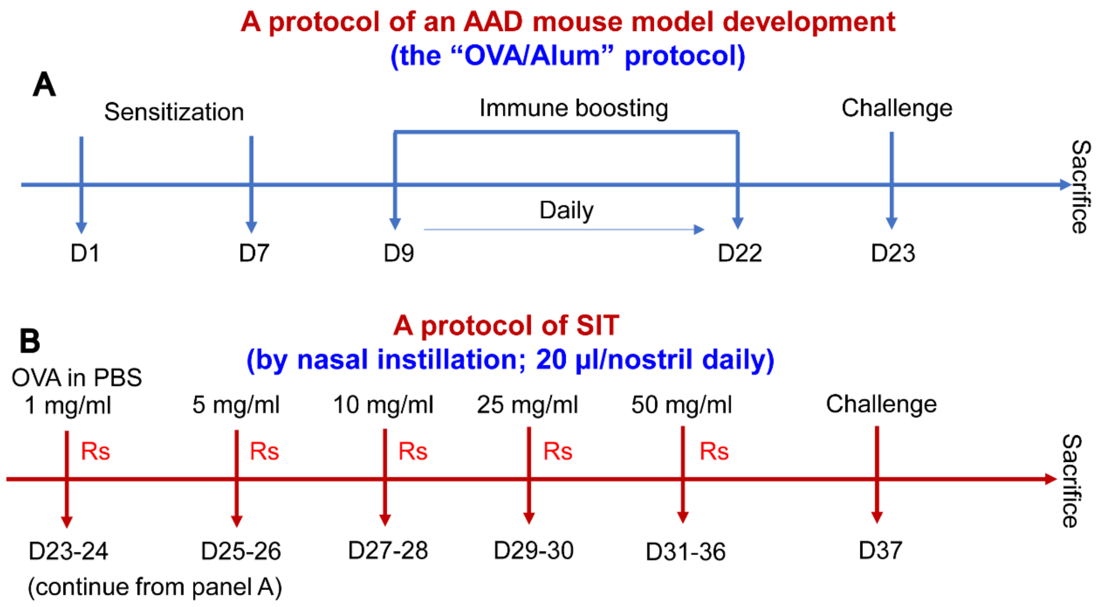

Figure S1. Protocols of AAD mouse model development and treating AAD mice with SIT. A, sensitization: Mice were injected with ovalbumin (100 µg OVA/mouse mixed in 0.1 ml Alum) into the back skin on day 1 and day 7, respectively. Immune boosting: Mice received nasal instillation (20 µl/nostril containing 5 mg OVA/ml)

daily from day 9 to day 22. Challenge: Mice were treated with nasal instillation (20µl/nostril containing 50 mg OVA/ml). B, a SIT protocol. Mice received nasal instillation containing OVA of indicated concentrations (control mice were treated with PBS-nasal instillation) and indicated dates. Rs: Mice were treated with restraint stress.

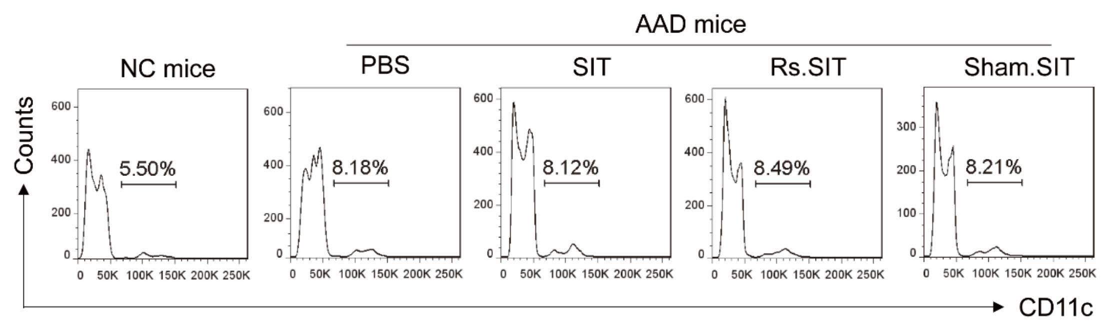

Figure S2. Assessment of DCs in AMCs. Mice were treated with the procedures denoted above each FCM plot. AMCs were isolated from the mouse airway tissues, and analyzed by FCM. Gated histograms show the DC counts. Summarized DC counts are presented in Fig. 2. AAD: Airway allergic disorder.

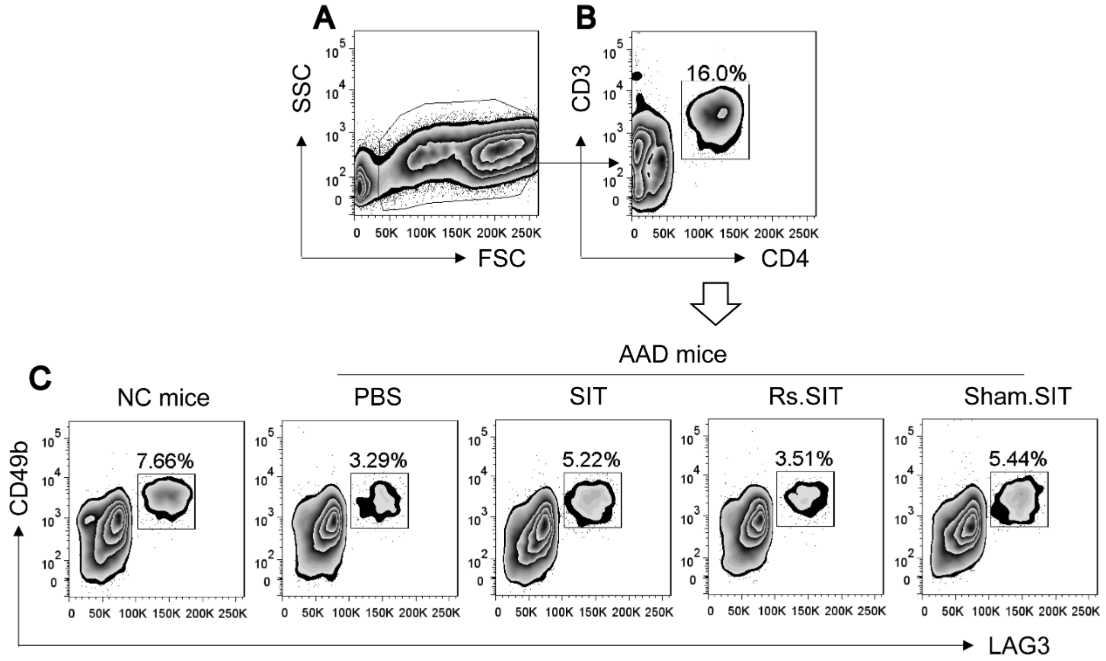

Figure S3. Tr1 cell counts in AMCs. Mice were treated with the procedures denoted above each FCM plot. AMCs were isolated from the mouse airway tissues, and analyzed by FCM. A, the FSC/SSC plots. B, $CD3^+$ $CD4^+$ T cells were gated. C, gated FCM plots show Tr1 cell counts in $CD4^+$ T cells. The summarized Tr1 cell data are presented in Fig. 2. Tr1 cell: Type 1 regulatory T cell.

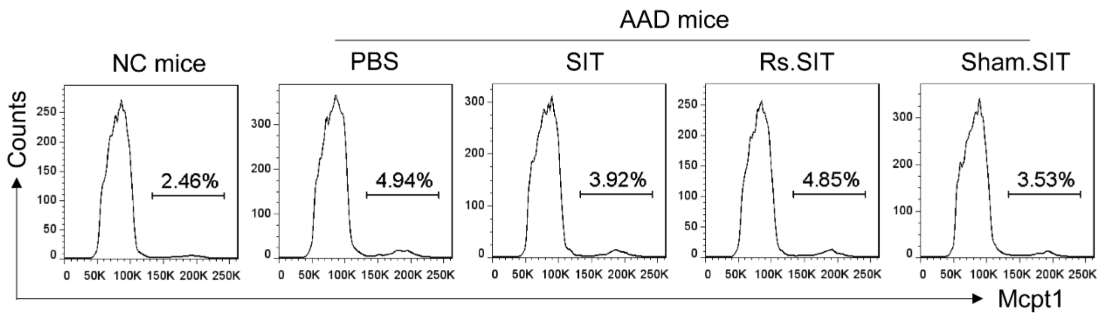

Figure S4. Mast cell counts in AMCs. Mice were treated with the procedures denoted above each FCM plot. AMCs were isolated from the mouse airway tissues, and analyzed by FCM. Gated histograms show the mast cell counts. Summarized mast cell counts are presented in Fig. 2. AAD: Airway allergic disorder.

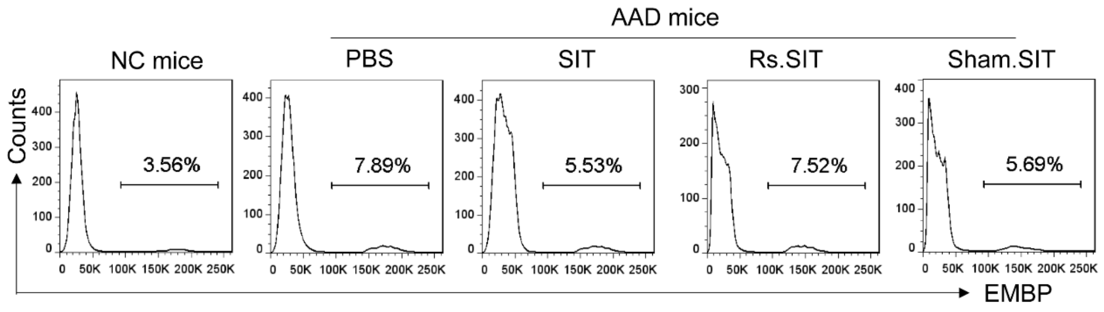

Figure S5. Eosinophil counts in AMCs. Mice were treated with the procedures denoted above each FCM plot. AMCs were isolated from the mouse airway tissues, and analyzed by FCM. Gated histograms show the eosinophil counts. Summarized eosinophil counts are presented in Fig. 2. AAD: Airway allergic disorder. EMBP: Eosinophil major basic protein.

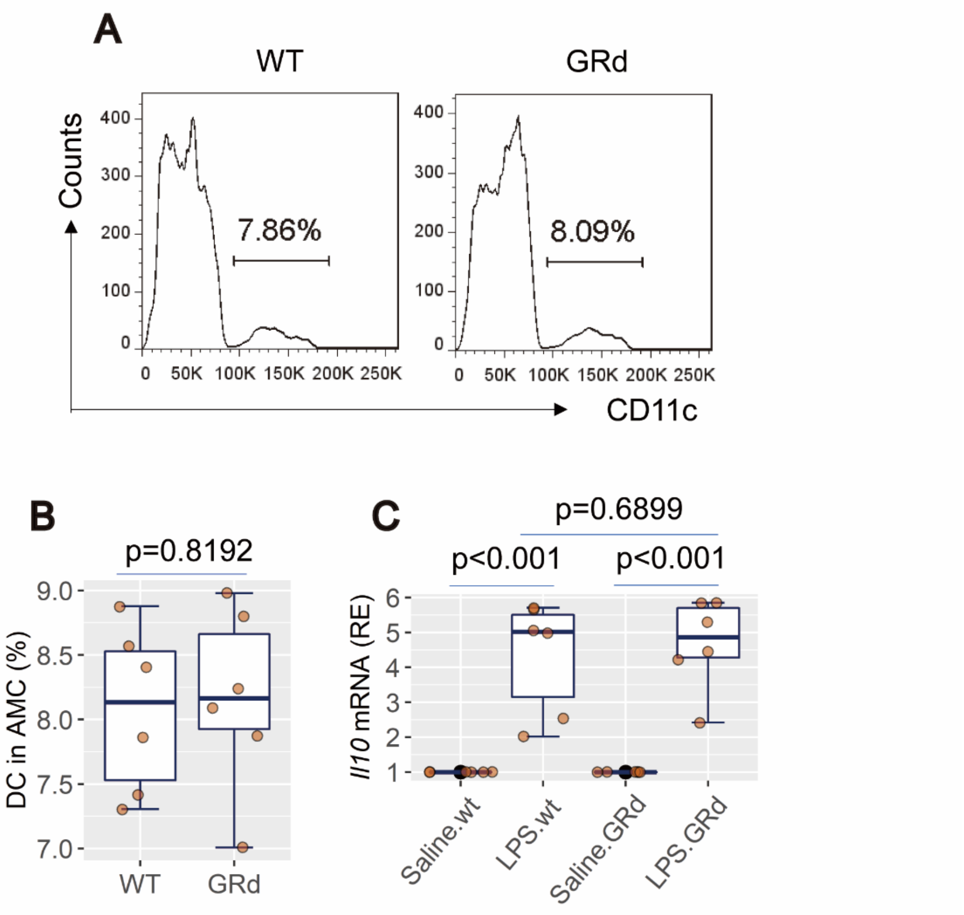

Figure S6. Assessment of DCs in GRd mice. AMCs were prepared with the airway tissues of WT mice and GRd mice (*GR*$^{\Delta DC}$ mice; mice carry the GR-deficient DCs). A, gated histograms show DC counts in AMCs. B, boxplots show median (IQR) of DC counts of 6 mice per group. C, DCs were isolated from AMCs, and cultured in the presence of LPS (100 ng/ml) overnight. DCs were then analyzed by RT-qPCR. Boxplots show median (IQR) of mRNA levels from 6 independent experiments. AMC: Airway mononuclear cells. GR: Glucocorticoid receptor. WT: Wild type.

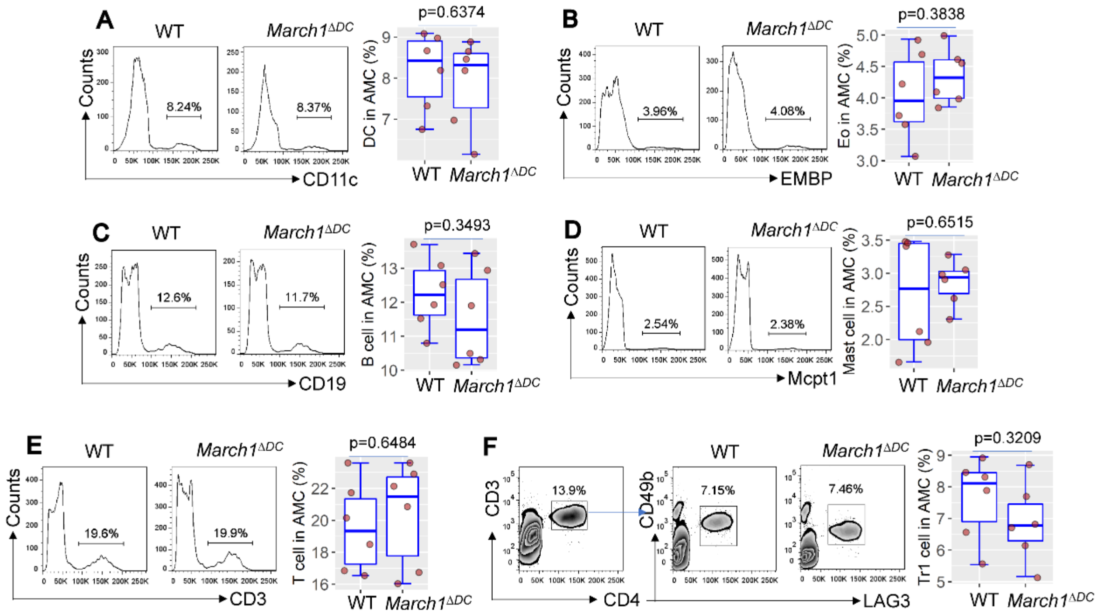

Figure S7. Characterization of immune cells in the airway tissues of *MARCH1*$^{\Delta DC}$ mice. AMCs were prepared from the airway tissues collected from WT mice and *MARCH1*$^{\Delta DC}$ mice, and analyzed by FCM. The gated FCM plots show counts of DC (A), eosinophil (Eo) (B), B cell (C), mast cell (D), T cell (E), and Tr1 cell (F) in AMCs. Boxplots on the right side of FCM plots show median (IQR) of indicated cell counts of 6 mice per group. AMC: Airway mononuclear cell. *MARCH1*$^{\Delta DC}$ mice: Mice carry *MARCH1*-deficient DCs. FCM: Flow cytometry. EMBP: Eosinophil major basic protein.

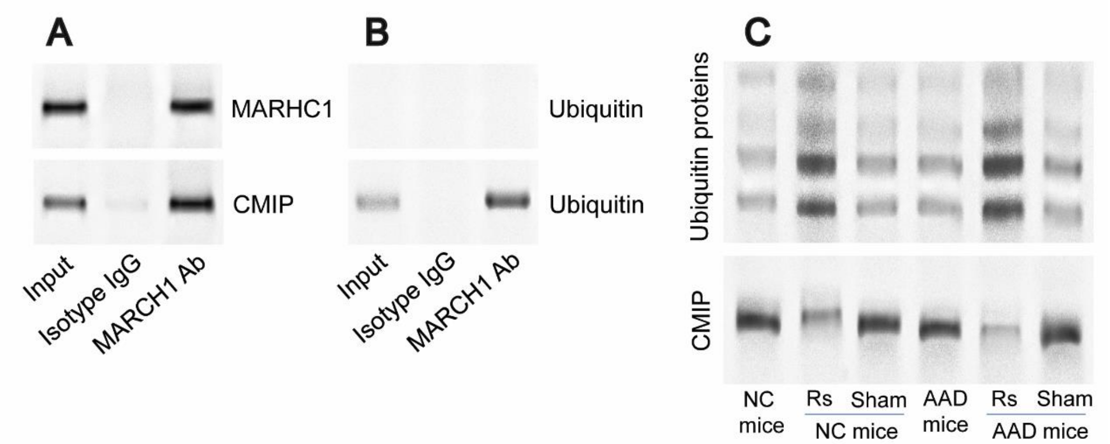

Figure S8. MARCH1 induces CMIP ubiquitination in DCs. A, DCs were isolated from the airway tissues of AAD mice treated with the Rs.SIT protocol. Protein extracts of the DC were analyzed by IP. Immunoblots show a complex of MARCH1 and CMIP. B, ubiquitin staining in the same membrane of CMIP-stained by the stripping-re-staining procedures. C, DCs were isolated from the airway tissues of mice treated with the procedures denoted below the blots. Protein extracts of DCs were processed by IP and stained with Abs of ubiquitin, and then CMIP Ab. Immunoblots show ubiquitin staining (the upper graph) and CMIP staining (the lower graph). The data represent 3 independent experiments.

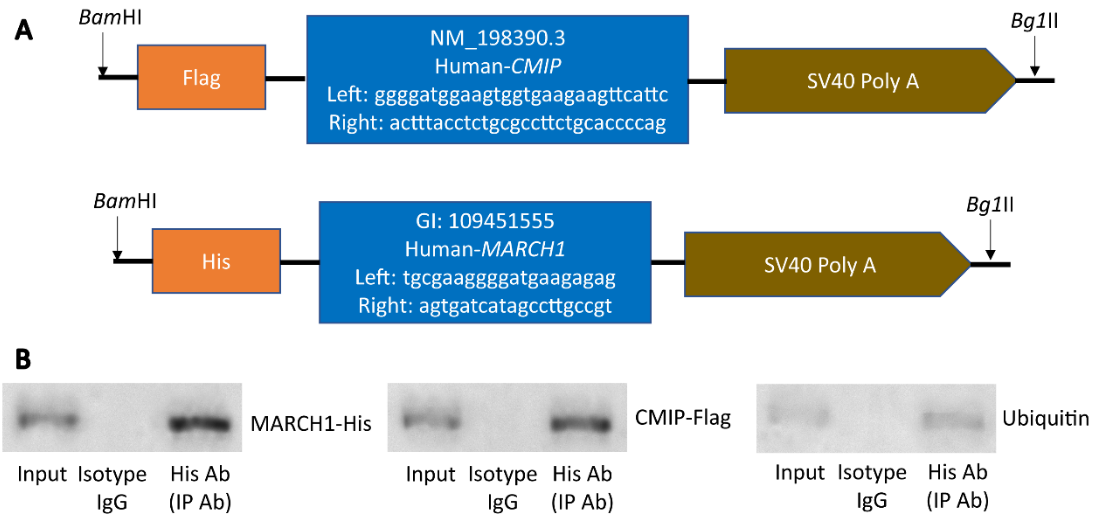

Figure S9. Schematics of a MARCH1-expressing plasmid and a CMIP-expressing plasmid construction

A, schematics of MARCH1-plasmids and CMIP-plasmids (prepared by the Sangaong Biotech, Shanghai, China). The plasmids were transfected to HEK293 cells following the manufacturer’s instruction. B, protein extracts of the HEK293 cells were analyzed by IP. Immunoblots show a complex of MARCH1 and CMIP, and ubiquitin in the same membrane of CMIP-stained. The data represent 3 independent experiments.

Gel graphs

Fig. 5

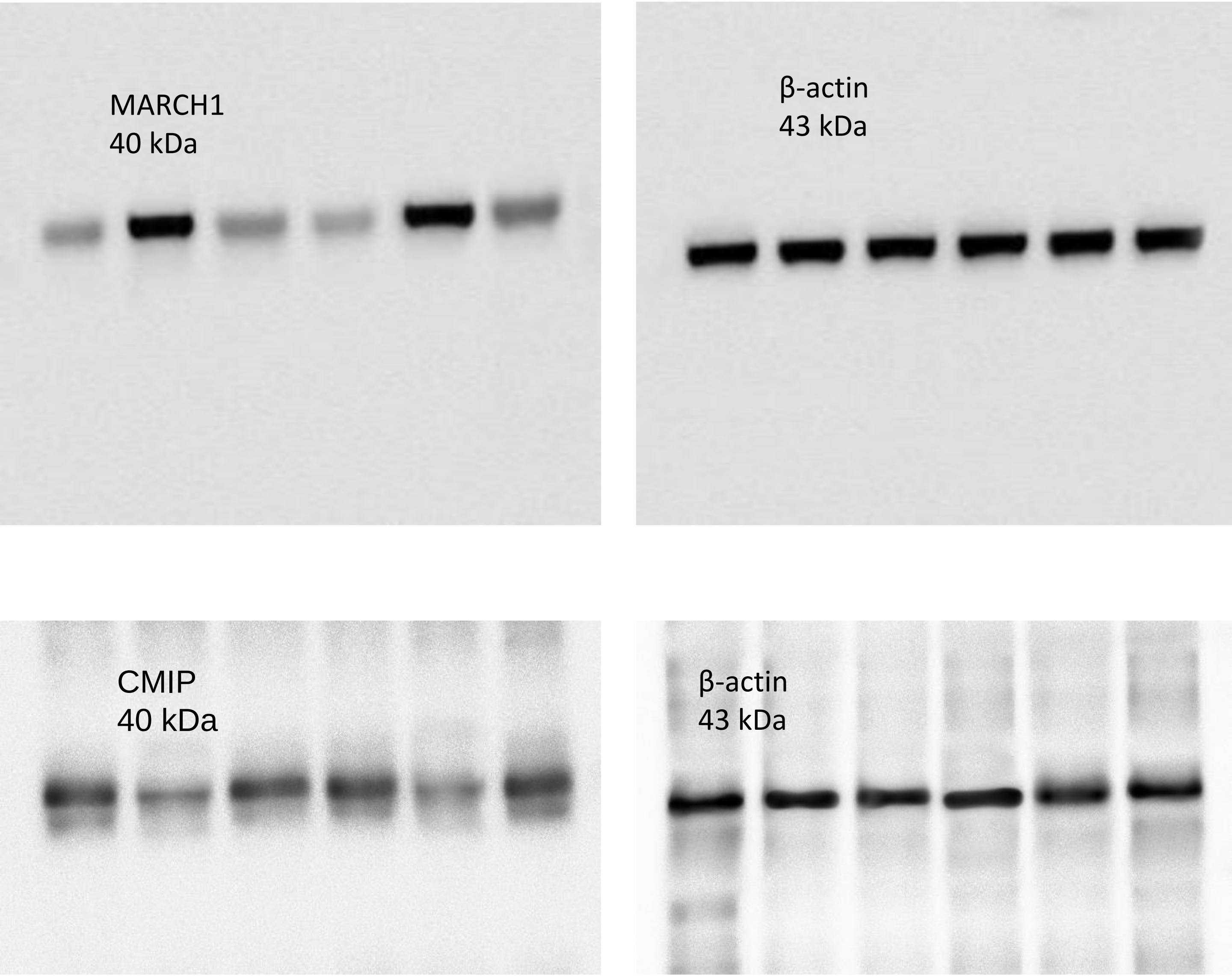

Fig. s8

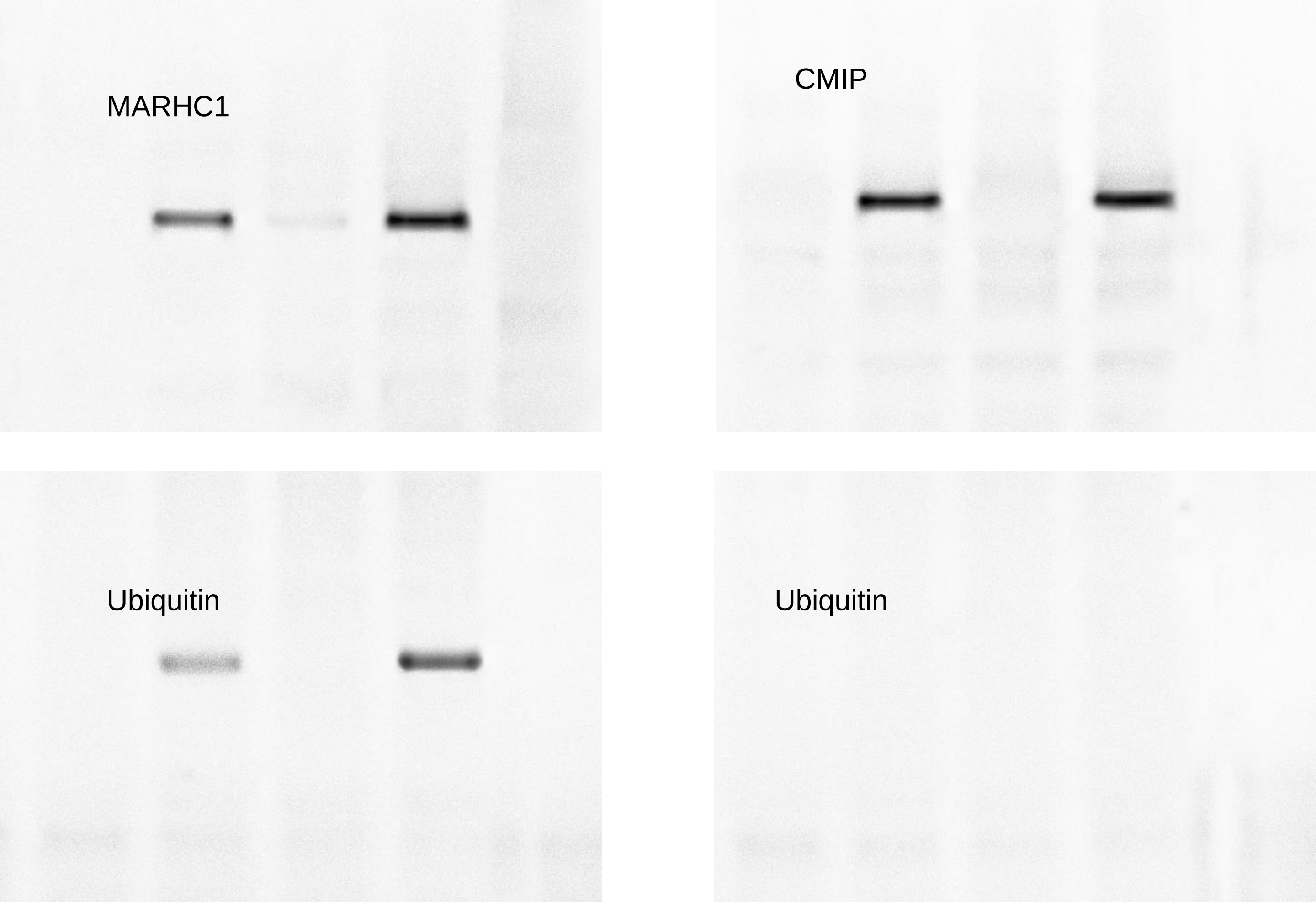

Fig. S8

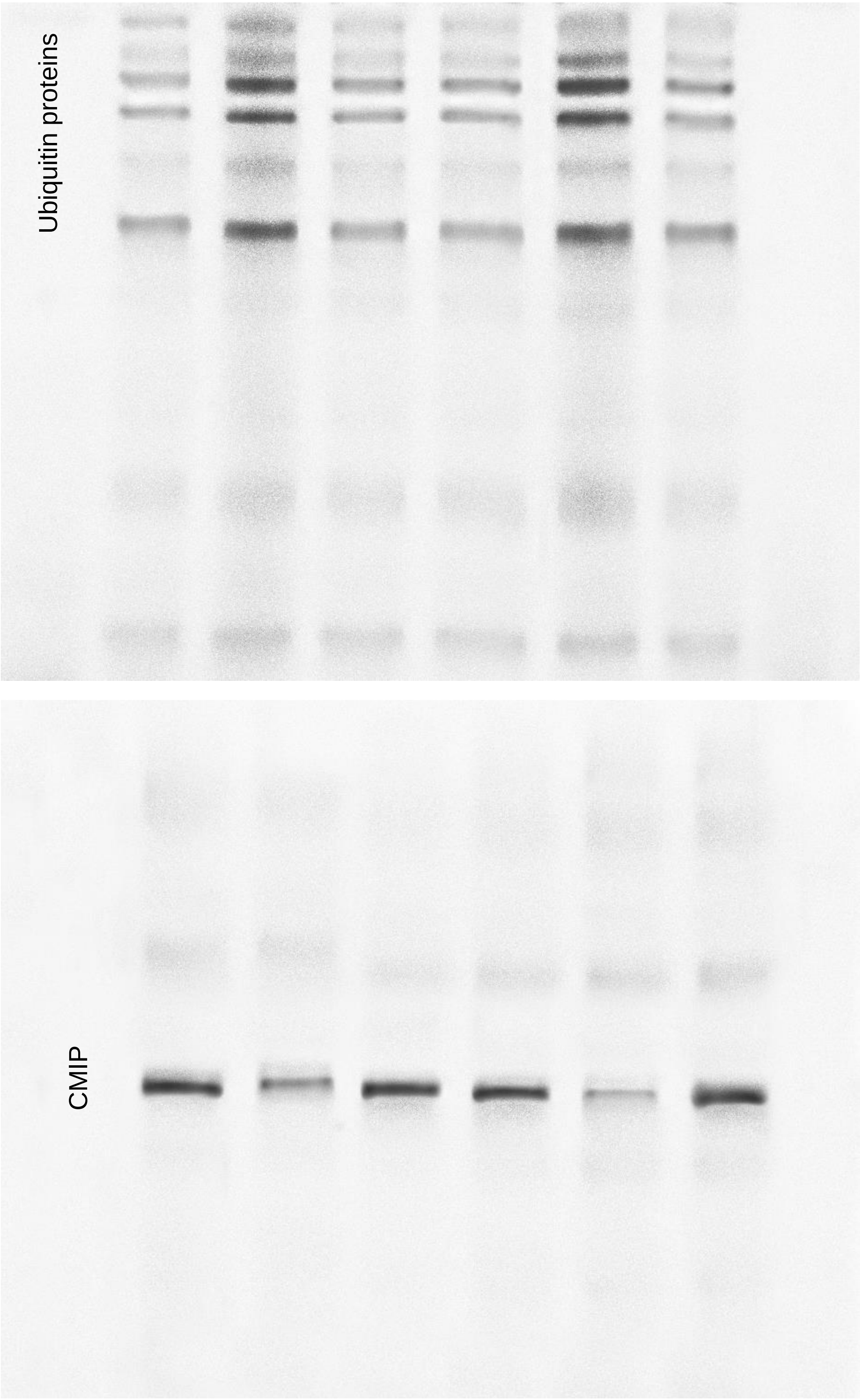

Fig.S9

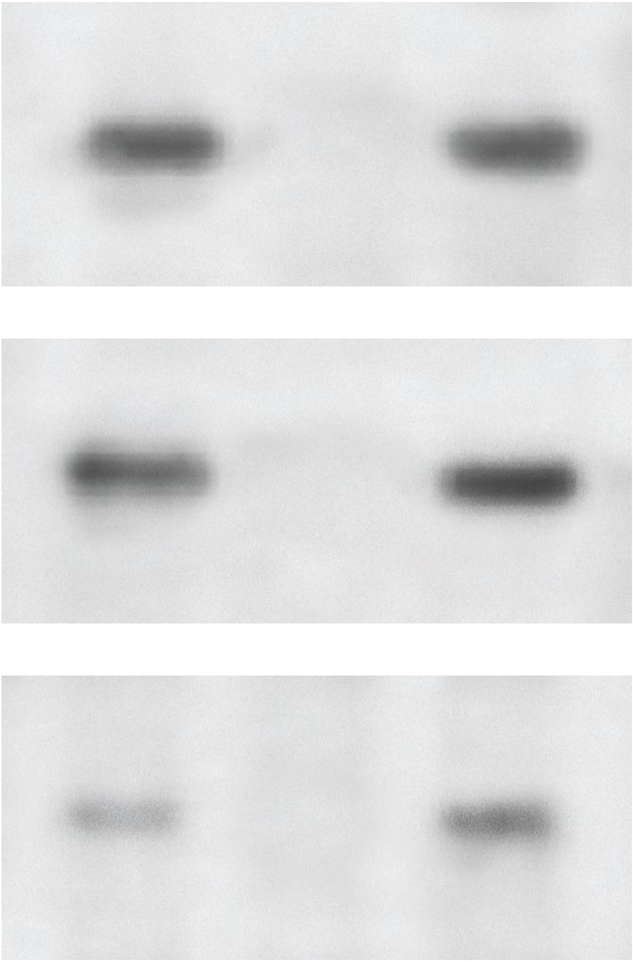

Gene	baseMean	log2FoldCl	stat	pvalue	padj
MT-RNR2	240410	0.065883	0.931092	0.351806	0.997402
FTL	20052.47	0.746343	10.31439	6.07E-25	3.00E-22
GLUL	6047.849	0.593013	7.529535	5.09E-14	1.01E-11
HPGD	7872.19	0.409935	5.19667	2.03E-07	1.91E-05
VIM	6573.853	0.416614	5.226438	1.73E-07	1.70E-05
MT-CO1	9062.181	-0.09697	-1.21379	0.224829	0.997402
PSAP	4898.381	0.069152	0.840846	0.400434	0.997402
BEST1	3986.286	0.301744	3.605658	0.000311	0.012946
EEF1A1	5835.594	0.125916	1.503197	0.132788	0.983401
CPA3	6824.657	-0.53227	-6.32745	2.49E-10	3.58E-08
FTH1	4310.338	0.300893	3.53988	0.0004	0.015996
TPT1	3593.442	0.027199	0.31867	0.749977	0.997402
ACTB	16827.83	0.28453	3.310653	0.000931	0.031809
HSPA8	3359.551	0.247823	2.864286	0.00418	0.108027
ACTG1	4095.46	0.316284	3.653695	0.000258	0.011003
LAPTM5	4576.284	-0.01345	-0.1553	0.876587	0.997402
ALAS1	3489.378	1.673476	19.2882	6.75E-83	2.42E-79
LCP1	3266.661	0.702183	8.056296	7.86E-16	1.98E-13
SH3BGRL3	3349.959	-0.1308	-1.49181	0.135748	0.990539
MT-ND4	4982.29	0.196818	2.210176	0.027093	0.422709
GAPDH	4490.79	0.048851	0.548207	0.58355	0.997402
B2M	5343.368	0.245832	2.753671	0.005893	0.142798
ANXA1	3111.474	-0.21723	-2.42396	0.015353	0.286594
CTSB	5719.749	0.589966	6.486371	8.79E-11	1.34E-08
HDC	3862.96	0.486429	5.323271	1.02E-07	1.04E-05
SRGN	4018.504	0.449895	4.900819	9.54E-07	8.05E-05
RPS6	2484.481	0.302375	3.278932	0.001042	0.034843
AL133415.	2258.459	0.446759	4.811692	1.50E-06	0.000121
ENO1	2374.599	0.105268	1.129244	0.258795	0.997402
TMSB4X	2768.915	0.012364	0.132352	0.894706	0.997402
ARHGDIB	2300.806	0.013118	0.140169	0.888526	0.997402
VWA5A	2097.081	-0.49143	-5.24558	1.56E-07	1.54E-05
CTSD	2098.003	-0.70968	-7.56653	3.83E-14	7.87E-12
HPGDS	2387.774	-0.22395	-2.38339	0.017154	0.310307
RAC2	3040.249	-0.28988	-3.08128	0.002061	0.06068
RPL4	1954.213	0.117038	1.239521	0.215153	0.997402
RPL5	2310.682	0.226154	2.391787	0.016767	0.305722
SQSTM1	2408.896	0.190054	2.007733	0.044672	0.571136
RPS4X	2313.553	0.00376	0.039652	0.968371	0.997402
ALDOA	1964.286	0.246527	2.592625	0.009525	0.206391
TIMP1	2353.935	-0.06499	-0.68137	0.495634	0.997402
CAPG	2742.804	0.006959	0.072813	0.941955	0.997402
HSP90AB1	2246.009	0.221833	2.303114	0.021272	0.358161
RACK1	2241.907	0.185019	1.911054	0.055998	0.655744
CTSG	1826.93	-0.89036	-9.16022	5.18E-20	1.86E-17
MT-CYB	3498.313	0.022898	0.235372	0.81392	0.997402
MT-ND2	1946.349	0.321007	3.282829	0.001028	0.034578
RPS8	1775.254	0.145147	1.478697	0.139221	0.997402
CD63	2753.285	-0.2272	-2.31164	0.020798	0.354625

AL138963.	2027.028	-0.00642	-0.06488	0.948272	0.997402
RPL10	1696.227	0.227551	2.292154	0.021897	0.366095
TPSAB1	3687.063	-0.80986	-8.14587	3.77E-16	1.00E-13
MT-ND1	2857.334	0.249854	2.512342	0.011993	0.242657
GRN	1577.42	-0.24973	-2.49892	0.012457	0.249355
RPL19	1632.594	0.0148	0.147887	0.882432	0.997402
RPLP1	1595.788	0.334472	3.339926	0.000838	0.029487
CCL2	1671.073	0.688122	6.869692	6.43E-12	1.12E-09
YWHAZ	1629.004	0.00014	0.001396	0.998886	0.999695
RPS3	2457.711	0.472984	4.708861	2.49E-06	0.000188
MT-CO2	5664.364	0.229165	2.2792	0.022655	0.372452
MPO	2096.2	0.245311	2.438923	0.014731	0.278778
MT-ND4L	2552.393	0.28556	2.836775	0.004557	0.115909
ARPC2	1593.895	-0.14109	-1.40162	0.161028	0.997402
FCER1G	1500.978	-0.1879	-1.86417	0.062298	0.695693
ACTR3	1687.22	-0.0895	-0.88744	0.37484	0.997402
TUBB	2010.384	-0.03639	-0.35947	0.719247	0.997402
AC006064	2171.585	0.050397	0.497037	0.619163	0.997402
MALAT1	6926.09	0.423884	4.177461	2.95E-05	0.001633
RPL3	3141.949	0.278571	2.723312	0.006463	0.153892
LDHA	1734.421	-0.34135	-3.33548	0.000852	0.029793
TUBA1B	1464.129	-0.22178	-2.16609	0.030304	0.447696
RPS18	1547.694	0.117565	1.147084	0.251347	0.997402
RPS12	1524.675	0.104441	1.012692	0.311208	0.997402
CYP1B1	1777.484	-0.04031	-0.38962	0.696817	0.997402
RPLP0	1560.225	0.386851	3.737256	0.000186	0.008186
PGK1	1355.47	0.127609	1.231274	0.21822	0.997402
RPS5	1410.205	0.196201	1.888245	0.058993	0.67932
RPL13A	1529.348	0.316085	3.017045	0.002553	0.07294
HSPA5	1504.721	0.17633	1.681484	0.092669	0.838079
RHEX	1346.176	-0.35445	-3.37988	0.000725	0.026138
MYL6	1435.231	-0.09412	-0.8927	0.372016	0.997402
S100A6	1293.89	-0.80161	-7.56623	3.84E-14	7.87E-12
EEF2	1254.512	0.331174	3.124174	0.001783	0.05419
MRNIP	1252.521	0.153613	1.440542	0.149714	0.997402
SIGLEC6	1425.545	-0.11701	-1.0972	0.272553	0.997402
RPS14	1316.121	0.15436	1.445639	0.148279	0.997402
AC011603	1273.902	-0.23925	-2.23889	0.025163	0.401978
TMSB10	1219.539	-0.304	-2.84422	0.004452	0.114247
RPL15	1311.104	0.20946	1.947267	0.051503	0.62452
CTSC	1383.175	1.702754	15.80533	2.86E-56	6.84E-53
CFL1	1793.664	0.196054	1.818258	0.069025	0.729765
RPL11	1236.637	0.07005	0.64966	0.515912	0.997402
CALR	1250.5	0.113002	1.045509	0.295788	0.997402
RPL8	1237.295	0.261041	2.411275	0.015897	0.29413
MT-ND5	4090.711	0.194803	1.795287	0.072608	0.748787
TMBIM6	1213.141	0.111911	1.03075	0.302658	0.997402
OAZ1	1280.54	-0.00879	-0.08097	0.935469	0.997402
DDX5	1794.795	0.061895	0.56988	0.568759	0.997402
CLIC1	1710.637	-0.17384	-1.59493	0.110728	0.919407

RPL37A	1224.795	0.043483	0.396297	0.691886	0.997402
MT-RNR1	6272.272	-0.1543	-1.40441	0.160196	0.997402
ATP5F1B	1276.85	0.26181	2.373666	0.017612	0.316209
VIM-AS1	1556.033	0.366845	3.322557	0.000892	0.030981
ALOX5AP	1130.369	-0.30345	-2.74204	0.006106	0.147207
UBXN11	1111.614	-0.00405	-0.03644	0.970932	0.997402
TPSB2	2953.215	-0.5641	-5.05308	4.35E-07	3.90E-05
RPL7A	1143.534	0.197955	1.769794	0.076761	0.764152
CTSL	1327.377	1.968033	17.53005	8.45E-69	2.42E-65
PFN1	1325.17	0.169341	1.507685	0.131635	0.981137
RHOA	1225.601	0.228484	2.030297	0.042326	0.553484
CAPZA1	1055.217	0.110767	0.983068	0.325574	0.997402
C6orf62	1065.96	0.117557	1.041818	0.297496	0.997402
CD52	1018.307	0.386953	3.427388	0.000609	0.022945
RPL13	1174.612	0.211232	1.870072	0.061474	0.692117
TPM4	1349.101	-0.23991	-2.12154	0.033877	0.483584
HNRNPA1	1056.085	0.1047	0.924517	0.355217	0.997402
CLU	1243.797	0.73629	6.494278	8.34E-11	1.29E-08
SDCBP	1408.422	-0.23596	-2.07841	0.037672	0.516145
SAT1	1050.697	-0.64068	-5.63117	1.79E-08	1.98E-06
LGALS1	1126.222	0.232398	2.041257	0.041225	0.544045
ARF1	1032.105	-0.06895	-0.6048	0.545313	0.997402
P4HB	1003.046	0.275342	2.414797	0.015744	0.292548
HNRNPK	1100.328	-0.03414	-0.2994	0.764633	0.997402
LDHB	1101.121	-0.1286	-1.12656	0.259928	0.997402
ITGB2	1024.711	0.307629	2.688719	0.007173	0.167045
H3-3B	1102.601	-0.05214	-0.45435	0.649579	0.997402
SLC18A2	1368.753	-1.12989	-9.82404	8.87E-23	3.86E-20
TPM3	1165.029	0.101748	0.882997	0.377238	0.997402
ESYT1	967.6371	-0.48694	-4.21976	2.45E-05	0.001381
SAMSN1	1163.109	0.283503	2.450658	0.01426	0.274383
CALM1	980.5529	0.294465	2.541339	0.011043	0.228256
S100A4	1121.863	-0.21261	-1.83452	0.066576	0.717535
RPS13	930.2586	0.17242	1.487138	0.136978	0.990899
RPL31	965.7755	0.074447	0.640304	0.521975	0.997402
NACA	917.7238	0.065271	0.559483	0.575832	0.997402
DUSP6	1059.395	-0.3874	-3.32039	0.000899	0.031072
PKM	2443.2	0.301549	2.581541	0.009836	0.209873
RPN2	927.905	0.308096	2.636201	0.008384	0.187345
PGD	901.9296	0.257713	2.200284	0.027787	0.426767
WARS1	913.9137	-0.05113	-0.4361	0.662767	0.997402
HSP90AA1	1045.874	0.130712	1.113765	0.26538	0.997402
SLC25A3	928.0904	0.110442	0.940352	0.347037	0.997402
ASAH1	964.5309	-0.51708	-4.3995	1.09E-05	0.000721
MT-CO3	1369.707	-0.10243	-0.87103	0.383739	0.997402
MS4A2	955.132	0.012956	0.110179	0.912268	0.997402
MAT2A	992.8019	0.124798	1.06103	0.288676	0.997402
HNRNPA2I	931.5876	0.13388	1.136201	0.255872	0.997402
HLA-E	898.7621	-0.48868	-4.14566	3.39E-05	0.001831
PABPC1	1086.831	0.40037	3.386112	0.000709	0.025803

ARHGAP18	1171.243	-1.39925	-11.8217	3.01E-32	2.70E-29
MYL12A	874.1182	0.244314	2.060703	0.039331	0.529773
ATP5F1A	895.5935	0.137867	1.162611	0.244988	0.997402
ENPP3	937.4925	1.152912	9.712562	2.67E-22	1.09E-19
PTPN6	901.654	0.196426	1.65442	0.098042	0.859956
PLD3	1094.571	0.089253	0.749815	0.453366	0.997402
BTK	963.6286	-0.78566	-6.59051	4.38E-11	7.06E-09
SLC25A6	1045.718	0.23916	2.004875	0.044976	0.57299
NEAT1	1820.755	0.069823	0.584858	0.558643	0.997402
SARAF	908.9629	0.110559	0.922429	0.356305	0.997402
ANXA4	924.4758	-0.62525	-5.21188	1.87E-07	1.80E-05
EIF4G2	1257.645	0.234134	1.949003	0.051295	0.622528
RPL30	860.6326	0.037478	0.311405	0.755492	0.997402
SNHG29	828.9066	-0.12028	-0.99928	0.317658	0.997402
AL662797.	852.7862	-0.01352	-0.11225	0.910627	0.997402
RPL28	866.4821	0.127127	1.054857	0.291491	0.997402
RPS11	900.1352	0.079705	0.659911	0.509311	0.997402
DAZAP2	830.5956	-0.05589	-0.46234	0.643839	0.997402
TYROBP	834.2448	-0.15897	-1.31363	0.188969	0.997402
TKT	857.812	0.238851	1.967476	0.049128	0.608065
HSP90B1	839.71	0.35988	2.957018	0.003106	0.086692
TPI1	989.591	0.057067	0.468569	0.639378	0.997402
S100A11	950.8734	0.03765	0.30848	0.757717	0.997402
RPS24	818.5043	0.201604	1.64859	0.099232	0.866389
PRDX1	918.4706	-0.1614	-1.31895	0.187185	0.997402
TXNIP	859.0223	-0.11643	-0.94947	0.342384	0.997402
CALM2	901.185	0.024574	0.200386	0.841179	0.997402
RPS23	993.6363	0.050694	0.413006	0.679602	0.997402
RPSA	808.5062	0.253645	2.06203	0.039205	0.528566
PTGS1	800.2815	-0.54378	-4.41381	1.02E-05	0.000684
ID2	929.4317	-0.02207	-0.17902	0.857921	0.997402
CORO1A	772.0162	0.158847	1.287556	0.197901	0.997402
HLA-A	1069.59	0.191431	1.55132	0.120825	0.952666
PRDX6	832.0026	-0.66674	-5.39622	6.81E-08	7.07E-06
HNRNPC	837.1979	0.214804	1.736725	0.082436	0.78836
RPL34	770.0577	0.174089	1.407184	0.159373	0.997402
AC068580.	792.995	-0.67426	-5.44868	5.07E-08	5.35E-06
PPIA	792.5593	0.066569	0.537935	0.590622	0.997402
EIF1	1083.813	0.067651	0.54584	0.585176	0.997402
CD53	862.5643	0.087157	0.703044	0.482028	0.997402
CD44	1540.638	-0.14339	-1.15634	0.247544	0.997402
GM2A	792.2291	-0.0157	-0.12656	0.89929	0.997402
KIT	2982.578	-1.15553	-9.30758	1.31E-20	5.07E-18
HLA-B	816.0016	0.182985	1.468927	0.141853	0.997402
LIPA	979.8954	1.971201	15.75758	6.09E-56	1.25E-52
SEPTIN2	963.3031	-0.24443	-1.95392	0.050711	0.619101
PLAT	907.7284	-1.51386	-12.0997	1.06E-33	1.09E-30
RPL18	738.518	0.230545	1.83628	0.066316	0.716338
TPSD1	1621.404	-0.50873	-4.04357	5.26E-05	0.002714
RPS20	732.3265	0.155297	1.233999	0.217203	0.997402

ATG10	766.9249	0.148147	1.173582	0.240563	0.997402
MT-ND6	745.4293	0.060217	0.476618	0.633634	0.997402
LGALS3	743.1107	-0.11553	-0.9135	0.360982	0.997402
AP003352.	722.0504	0.162145	1.28092	0.200222	0.997402
PDIA3	772.6987	-0.05311	-0.41919	0.675078	0.997402
RPL12	766.2885	0.276487	2.179599	0.029287	0.441063
MSN	1023.119	0.153537	1.209249	0.226567	0.997402
RPS27A	993.5675	0.002236	0.017601	0.985957	0.997402
RPS16	719.667	0.11136	0.875518	0.381292	0.997402
GPNMB	803.472	-1.27844	-10.0381	1.04E-23	4.96E-21
LAPTM4A	756.6987	0.039133	0.306976	0.758861	0.997402
SERPINB1	893.5662	-0.09553	-0.74789	0.454525	0.997402
NCL	761.0083	0.145593	1.136897	0.255581	0.997402
RPS19	934.0629	0.553862	4.323551	1.54E-05	0.000962
TUBA1A	744.7869	-0.67069	-5.22166	1.77E-07	1.73E-05
M6PR	699.5291	-0.03889	-0.30272	0.762106	0.997402
PTMA	760.8493	0.239673	1.862824	0.062487	0.695944
MT-ATP6	1141.926	0.173132	1.341784	0.179666	0.997402
CSTB	752.5808	1.021306	7.89972	2.80E-15	6.57E-13
CD82	780.1115	0.089042	0.688696	0.491015	0.997402
RPL23	684.7668	0.043999	0.340051	0.733818	0.997402
RPLP2	711.9913	0.163853	1.26605	0.205495	0.997402
VCP	713.1277	-0.1355	-1.0464	0.295378	0.997402
GSN	703.1579	0.49655	3.827714	0.000129	0.005947
ALDH1A1	746.743	-0.06605	-0.50896	0.610777	0.997402
MYL12B	682.7406	-0.15221	-1.17104	0.241584	0.997402
RPL7	722.1289	0.143043	1.099788	0.271425	0.997402
RPS9	716.7398	0.086738	0.666495	0.505095	0.997402
TALDO1	728.8184	0.15939	1.224629	0.220715	0.997402
AC020914.	708.7243	-0.119	-0.91388	0.360782	0.997402
RPL10A	888.0999	0.27588	2.114811	0.034446	0.486648
CNBP	705.5363	0.052563	0.402223	0.68752	0.997402
GPX1	704.8641	-0.02479	-0.18962	0.849609	0.997402
ARHGEF6	744.5543	-0.72307	-5.52796	3.24E-08	3.52E-06
ENPP2	698.3633	-0.21356	-1.63094	0.102902	0.884443
UBB	672.138	-0.24785	-1.89242	0.058435	0.676556
RBM3	656.1717	-0.02112	-0.1612	0.871938	0.997402
ARPC5	806.3659	0.20125	1.532851	0.125313	0.968243
MT-ND3	683.2346	0.24583	1.871127	0.061328	0.691241
PTPN7	732.0314	1.035572	7.878338	3.32E-15	7.55E-13
NONO	664.5273	-0.11637	-0.88391	0.376745	0.997402
RAB27B	670.3716	-0.47611	-3.61004	0.000306	0.012766
ARPC1B	912.5915	-0.03617	-0.27421	0.783925	0.997402
FERMT3	703.4584	0.077238	0.583854	0.559318	0.997402
TAPBP	644.5116	0.136608	1.032335	0.301915	0.997402
VMP1	663.996	0.542453	4.092607	4.27E-05	0.002225
YWHAB	665.5324	-0.04033	-0.30396	0.761158	0.997402
APLP2	648.0037	0.080311	0.605251	0.545012	0.997402
CD74	728.2225	1.287898	9.701194	2.98E-22	1.19E-19
TAGLN2	740.0221	0.17354	1.302922	0.192602	0.997402

RPL32	623.3783	0.134033	1.005369	0.314719	0.997402
HLA-C	767.5348	0.12609	0.945047	0.344635	0.997402
CAP1	1369.737	-0.00145	-0.01089	0.99131	0.998821
ARL6IP5	698.4891	0.283475	2.12351	0.033711	0.483103
LPXN	670.2201	0.043555	0.326255	0.744231	0.997402
P2RX1	669.0503	-0.43553	-3.25807	0.001122	0.036737
THBS1	699.5956	-1.69924	-12.6748	8.15E-37	1.06E-33
PSMD1	612.0732	-0.43329	-3.22895	0.001242	0.039607
RPS27	612.3582	0.026951	0.200759	0.840887	0.997402
ARPC3	675.1661	-0.13524	-1.0061	0.314367	0.997402
AP2M1	722.5563	-0.04423	-0.32896	0.742187	0.997402
PPT1	616.449	0.446858	3.323093	0.00089	0.030981
RAN	704.1341	0.191414	1.421611	0.155139	0.997402
NCOA4	717.7205	-0.77432	-5.74804	9.03E-09	1.03E-06
HNRNPF	606.4191	-0.00668	-0.04946	0.960556	0.997402
RPL38	595.4218	0.119391	0.881251	0.378182	0.997402
PTAFR	608.4187	-0.46015	-3.3953	0.000686	0.025216
TPP1	1072.57	-0.14478	-1.06814	0.285459	0.997402
EIF3E	606.9082	0.144595	1.065777	0.286524	0.997402
SRP14	591.8492	-0.00019	-0.00141	0.998874	0.999695
SERF2	702.5638	0.005461	0.040117	0.968	0.997402
CSDE1	615.9396	0.082951	0.609352	0.542291	0.997402
RPL29	604.7162	0.039026	0.285993	0.774883	0.997402
LEO1	606.4673	-0.0151	-0.10985	0.912531	0.997402
LRRC75A	571.2057	-0.1092	-0.79315	0.427691	0.997402
ATP6V0E1	567.7813	0.004468	0.032395	0.974157	0.997402
JSRP1	578.3204	0.125476	0.909545	0.363062	0.997402
S100A9	566.0101	0.059964	0.43419	0.664151	0.997402
SLC39A11	567.8642	-0.4262	-3.0847	0.002038	0.060143
VAPA	564.0289	-0.33851	-2.44662	0.01442	0.27508
IFITM2	677.4018	-0.60563	-4.37606	1.21E-05	0.000795
SEC61A1	563.8967	0.106993	0.772321	0.439924	0.997402
AHNAK	1035.129	-0.30966	-2.2343	0.025464	0.404961
PDZD8	653.922	-0.93588	-6.73015	1.69E-11	2.83E-09
RPS25	557.6474	0.13778	0.990784	0.321791	0.997402
RPL35A	583.0501	0.087963	0.63105	0.528008	0.997402
RPL27A	548.8605	0.085874	0.615836	0.538003	0.997402
GHITM	547.4784	0.197127	1.412257	0.157874	0.997402
SH3BGRL	552.7351	-0.13801	-0.98769	0.323304	0.997402
SLC25A5	662.0607	0.223232	1.596959	0.110275	0.918885
ENO3	561.9791	0.12967	0.927238	0.353803	0.997402
NPM1	594.6821	0.044134	0.315453	0.752417	0.997402
CCT7	547.1694	0.081795	0.584418	0.558939	0.997402
RPL27	553.233	0.181085	1.293748	0.195753	0.997402
MTHFD2	576.6303	-0.47241	-3.37127	0.000748	0.026766
VAT1	615.1968	-0.38912	-2.77639	0.005497	0.13548
GPI	566.4199	0.265641	1.893462	0.058296	0.675494
COX4I1	602.3926	0.165809	1.180938	0.237627	0.997402
SYNGR2	606.1654	1.043279	7.416116	1.21E-13	2.34E-11
PPIB	690.4007	0.186209	1.321414	0.186363	0.997402

CTSA	540.0675	0.012636	0.08963	0.928581	0.997402
LGALS8	548.3084	-0.59804	-4.24008	2.23E-05	0.001292
SNX22	558.6555	0.039089	0.275833	0.782677	0.997402
LASP1	560.9628	0.231196	1.63126	0.102835	0.884397
ARHGAP25	600.9799	-0.45471	-3.20809	0.001336	0.042127
IFITM3	721.9637	-1.51338	-10.6698	1.41E-26	7.77E-24
AC093484.	559.6837	-0.2344	-1.64708	0.099541	0.867675
EIF4A2	521.6102	0.012748	0.089411	0.928755	0.997402
CCT8	545.9682	0.194331	1.362918	0.172908	0.997402
PRTN3	755.404	0.539652	3.783438	0.000155	0.006912
RPS29	539.9478	-0.03545	-0.24845	0.803787	0.997402
SURF4	514.6118	0.082929	0.58057	0.561531	0.997402
NRP1	543.9746	-0.21861	-1.52972	0.126087	0.969144
PSME1	550.2306	0.05253	0.367554	0.713206	0.997402
WDR1	581.8818	0.109336	0.764635	0.444489	0.997402
ATP1B3	521.9873	-0.22717	-1.58642	0.112644	0.923356
TESPA1	554.0197	1.080961	7.54235	4.62E-14	9.33E-12
PLEKHB2	534.8629	0.32276	2.251667	0.024343	0.393693
GDI2	539.3517	0.149662	1.043522	0.296707	0.997402
XRCC5	521.7372	-0.00449	-0.03117	0.975136	0.997402
ATP6V1B2	611.8895	0.717225	4.975282	6.52E-07	5.66E-05
LYZ	1080.572	-1.69173	-11.7165	1.05E-31	8.36E-29
CD164	501.0163	-0.01612	-0.11154	0.911191	0.997402
SSR2	499.5979	0.163667	1.129568	0.258658	0.997402
CNN2	557.431	-0.13009	-0.89755	0.369427	0.997402
UCP2	611.2857	-0.18838	-1.2976	0.194425	0.997402
LAT2	517.5571	-0.87256	-6.01009	1.85E-09	2.42E-07
CCT3	509.1164	0.329886	2.26976	0.023222	0.37926
CD84	590.9936	-0.03228	-0.22191	0.824385	0.997402
DNAJA1	504.258	0.15239	1.044456	0.296275	0.997402
PSMA7	508.2192	0.263358	1.804121	0.071212	0.744009
IL1RL1	539.8704	-0.11463	-0.78524	0.432315	0.997402
PDIA6	525.8224	0.310049	2.121503	0.03388	0.483584
CD59	503.2642	-1.01991	-6.96595	3.26E-12	5.85E-10
CCT6A	493.5149	0.352909	2.407855	0.016047	0.296252
SET	519.8879	0.012771	0.087083	0.930606	0.997402
CMTM6	605.8156	0.028698	0.195342	0.845125	0.997402
HSPA9	517.7785	0.205646	1.397605	0.162232	0.997402
EEF1B2	533.4885	0.258038	1.752283	0.079725	0.780653
TSC22D1	522.4153	-0.66059	-4.4841	7.32E-06	0.0005
MBOAT7	493.1869	-0.49759	-3.37747	0.000732	0.026302
HCLS1	523.1193	-0.12961	-0.87897	0.379415	0.997402
CHP1	502.7465	-0.20834	-1.41132	0.158151	0.997402
RPN1	475.9408	0.138255	0.936539	0.348996	0.997402
MYH9	478.1448	-0.05804	-0.39245	0.694729	0.997402
RAB7A	504.0522	0.011611	0.078432	0.937485	0.997402
PIK3R6	491.4899	0.640459	4.318909	1.57E-05	0.00097
CCT5	496.7145	0.283552	1.911831	0.055898	0.655111
COPA	507.6449	-0.05904	-0.39804	0.6906	0.997402
HADHA	492.0725	0.150765	1.016363	0.309457	0.997402

DDOST	490.5016	-0.07611	-0.51228	0.608455	0.997402
PFDN5	478.1868	0.096831	0.651333	0.514831	0.997402
GLB1	484.9143	0.364829	2.452555	0.014185	0.273925
S100A8	607.6529	-0.14605	-0.98174	0.326226	0.997402
XRCC6	553.7222	0.003119	0.020955	0.983282	0.997402
SIGLEC10	486.6298	-0.76063	-5.10733	3.27E-07	3.01E-05
PEBP1	515.913	-0.40628	-2.72612	0.006408	0.153206
SRSF3	493.3235	0.237167	1.589349	0.111982	0.922675
RPL6	536.7401	0.348228	2.33324	0.019636	0.341286
CYP1B1-AS	584.0783	-0.12789	-0.85548	0.392288	0.997402
ELOVL5	472.3383	-0.03001	-0.20069	0.840938	0.997402
RPS4Y1	495.6338	0.11668	0.779931	0.435431	0.997402
VSIR	501.5237	-0.4963	-3.31734	0.000909	0.031338
CAVIN2	466.3264	-0.39717	-2.64889	0.008076	0.181864
UQCRC1	472.9665	0.392269	2.613024	0.008975	0.197151
ATP5F1C	486.5424	-0.12608	-0.83935	0.401271	0.997402
ATP6AP2	457.7341	0.287302	1.911867	0.055893	0.655111
SLC3A2	491.8736	0.42698	2.839417	0.00452	0.115362
COPB2	449.5879	-0.1572	-1.04354	0.296698	0.997402
PPP1CB	502.6489	0.003955	0.02624	0.979066	0.997402
UBE2D3	587.0074	-0.0578	-0.3833	0.701499	0.997402
EIF3D	461.2523	0.332593	2.202228	0.027649	0.426251
EIF3L	447.5572	0.219439	1.450604	0.14689	0.997402
NPC2	461.2785	0.281969	1.863476	0.062395	0.695693
EDEM2	464.6111	-1.02823	-6.77446	1.25E-11	2.13E-09
SNX32	574.1318	0.214344	1.410798	0.158304	0.997402
CAPN1	461.6177	-0.32044	-2.10731	0.035091	0.490621
RBMX	439.0176	-0.12127	-0.79714	0.42537	0.997402
MPP1	529.4724	0.086166	0.566243	0.571228	0.997402
ITGA2B	463.4287	1.229899	8.073383	6.84E-16	1.78E-13
CXCL8	600.2314	1.513232	9.921216	3.37E-23	1.56E-20
NDUFA4	464.6069	0.255167	1.670216	0.094877	0.84543
HNRNPDL	446.3377	0.129375	0.845414	0.39788	0.997402
SERINC1	445.0924	-0.29405	-1.91656	0.055294	0.653233
MTHFD1	473.4999	-1.1891	-7.74119	9.85E-15	2.14E-12
H2AZ1	442.3532	0.025027	0.162823	0.870658	0.997402
ATP5PB	426.4796	0.089612	0.582539	0.560203	0.997402
SSR4	446.7003	0.050113	0.325637	0.744699	0.997402
RHOG	430.1678	-0.23717	-1.53908	0.123784	0.962949
VDAC1	426.537	0.38486	2.496442	0.012545	0.250282
PLEK	495.6079	0.673579	4.368467	1.25E-05	0.000816
TMEM176	453.2288	-0.31241	-2.01968	0.043417	0.561093
CLC	612.085	-0.31188	-2.01542	0.043861	0.564291
UBC	452.2797	-0.01994	-0.12857	0.897702	0.997402
ANPEP	656.0149	2.070591	13.32617	1.63E-40	2.34E-37
ACSL4	680.6292	0.280577	1.804659	0.071128	0.744009
C3AR1	427.4071	-0.66944	-4.30332	1.68E-05	0.001031
BTG2	543.9961	1.968141	12.64146	1.25E-36	1.38E-33
CORO1C	423.8306	-0.31486	-2.02177	0.0432	0.559687
ATP6AP1	499.8711	-0.13926	-0.89395	0.371346	0.997402

SEPTIN7	437.9885	0.117171	0.752081	0.452003	0.997402
PARK7	419.3999	-0.00575	-0.0368	0.970642	0.997402
COX7C	412.9886	0.06694	0.428501	0.668286	0.997402
LITAF	578.1374	-0.88691	-5.67664	1.37E-08	1.54E-06
TM9SF2	447.4604	-0.10071	-0.64424	0.519423	0.997402
STT3A	418.8116	0.53887	3.440469	0.000581	0.022096
SSR1	436.6804	-0.02692	-0.17156	0.863786	0.997402
LMNA	562.5735	-0.0118	-0.07521	0.940048	0.997402
BTF3	431.7599	0.27926	1.779724	0.075121	0.756394
UBA52	445.6675	0.058663	0.373762	0.708581	0.997402
EIF3H	459.2217	0.108422	0.690331	0.489986	0.997402
MIR223HG	410.3583	0.324768	2.062684	0.039143	0.528223
ANXA5	403.0872	0.046219	0.293538	0.769111	0.997402
OS9	416.0591	0.048391	0.307268	0.75864	0.997402
CELF2	415.4271	-0.22049	-1.39935	0.161707	0.997402
NRBP1	433.7538	-0.0148	-0.09389	0.925195	0.997402
ATP5MC3	434.4392	0.367704	2.332833	0.019657	0.341286
EIF2S3	410.7835	0.01297	0.08228	0.934424	0.997402
MYD88	424.6985	-0.55897	-3.54315	0.000395	0.015887
ICAM1	420.2279	0.91027	5.767961	8.02E-09	9.21E-07
PAPSS1	404.8181	-0.58857	-3.72701	0.000194	0.0085
LMAN2	413.9614	0.204189	1.292843	0.196065	0.997402
FKBP1A	400.6303	0.355923	2.252279	0.024305	0.39351
NEK6	469.022	-0.13403	-0.8473	0.396829	0.997402
CAPZA2	457.439	0.058089	0.366962	0.713647	0.997402
FXYD5	443.9813	-0.22554	-1.4234	0.154621	0.997402
MMACHC	445.9613	-0.24502	-1.5434	0.122734	0.957903
MGAT1	417.8083	0.391162	2.463824	0.013746	0.268654
ILF2	400.0857	0.151205	0.951269	0.341468	0.997402
AL157895.	395.8431	-0.51154	-3.21577	0.001301	0.041196
TMED2	392.6	-0.09799	-0.61588	0.537973	0.997402
DBI	402.0605	-0.67453	-4.23853	2.25E-05	0.001296
MDH1	394.3493	0.337046	2.117122	0.03425	0.486445
MFSD1	431.0201	0.40219	2.52501	0.011569	0.23608
RPL37	488.1533	0.235219	1.476732	0.139748	0.997402
ATP6V1F	394.2099	-0.29807	-1.87103	0.061341	0.691241
ETNK1	402.9579	0.582087	3.649757	0.000262	0.011107
YWHAQ	402.2252	-0.05269	-0.33002	0.741384	0.997402
SLC2A3	399.2495	-0.8765	-5.48735	4.08E-08	4.37E-06
TBXAS1	397.4669	-0.13151	-0.82188	0.411146	0.997402
GSTO1	390.0661	0.017174	0.107229	0.914608	0.997402
LRPAP1	400.3167	0.255034	1.592243	0.11133	0.920631
RPL14	441.0452	0.341266	2.127981	0.033339	0.478706
HS3ST1	473.5782	1.136072	7.079973	1.44E-12	2.69E-10
RPS28	438.9239	0.057602	0.358488	0.719978	0.997402
FUCA1	501.3293	-1.79652	-11.1806	5.07E-29	3.47E-26
GPX4	389.2647	0.273741	1.702904	0.088586	0.818792
UQCRC2	391.0542	0.502339	3.122916	0.001791	0.054224
NCSTN	384.2742	0.185886	1.155316	0.247961	0.997402
HK1	408.798	-0.31702	-1.96922	0.048928	0.606108

MARS1	406.6051	0.159765	0.991873	0.32126	0.997402
SERINC3	446.1911	-0.54668	-3.39348	0.00069	0.025307
RPL36	394.2524	0.111019	0.688819	0.490937	0.997402
CALU	390.6373	0.043222	0.267676	0.788949	0.997402
ATP6V0B	410.0037	-0.15678	-0.97028	0.331907	0.997402
PTTG1IP	381.3978	-0.56815	-3.51551	0.000439	0.017277
STING1	393.2834	-0.95646	-5.91374	3.34E-09	4.17E-07
IL1B	417.4196	-0.84472	-5.21985	1.79E-07	1.74E-05
RPL23A	383.1404	0.142096	0.877577	0.380173	0.997402
ZEB2	501.8091	-0.10362	-0.63978	0.522314	0.997402
CD9	405.7961	-1.33631	-8.24284	1.68E-16	4.55E-14
DBNL	378.568	-0.02549	-0.15718	0.875104	0.997402
SELPLG	404.9356	0.240244	1.480906	0.138632	0.997225
MOB1A	379.9435	-0.16142	-0.99483	0.319817	0.997402
ANXA2	433.6997	1.234624	7.608077	2.78E-14	5.87E-12
MAPRE1	375.2585	-0.03474	-0.214	0.830546	0.997402
PSMB1	421.2159	-0.00883	-0.05428	0.956713	0.997402
HNRNPH1	402.872	0.011971	0.073578	0.941346	0.997402
RPL41	369.9838	0.186761	1.146917	0.251416	0.997402
GANAB	446.5477	0.005396	0.033136	0.973566	0.997402
DCBLD2	401.6612	0.07903	0.485303	0.627462	0.997402
IL12a	505.078	-0.02345	-0.14392	0.885564	0.997402
IL10	385.6257	-0.14943	-0.91622	0.359553	0.997402
TGFB1	394.87	-0.24362	-1.49337	0.135339	0.990539
TMCC3	373.2775	-0.17149	-1.04917	0.2941	0.997402
MTA3	376.0662	0.210447	1.284697	0.198898	0.997402
ATP11B	365.7472	-0.28352	-1.73071	0.083504	0.793815
MARCH1	365.9888	-0.04229	-0.25809	0.796338	0.997402
PPP1R12A	372.5393	0.437653	2.670451	0.007575	0.172925
CAMK2B	410.4136	-0.49367	-3.00826	0.002627	0.074784
CROCC	382.6319	0.074448	0.453191	0.650412	0.997402
TIMD4	494.104	-0.79724	-4.85209	1.22E-06	0.000101
ATP2B4	367.7111	0.165704	1.005194	0.314804	0.997402
ZC3H11A	360.3856	-0.16324	-0.98875	0.322785	0.997402
PIK3R1	398.635	0.200787	1.215177	0.224299	0.997402
STAT3	360.2403	0.132983	0.804656	0.421018	0.997402
TLR4	369.9042	0.025542	0.15417	0.877476	0.997402
TARBP1	359.3269	-0.09416	-0.56831	0.569823	0.997402
MXD1	370.609	0.106355	0.641788	0.521011	0.997402
CD86	354.5905	0.0779	0.469691	0.638576	0.997402
DNAJC25	437.9938	-0.22616	-1.36346	0.172737	0.997402
SLC2A3	371.6667	-0.1474	-0.88836	0.374348	0.997402
CTDP1	366.1172	0.306853	1.846409	0.064833	0.708764
IRE1	365.7949	0.217403	1.307691	0.190978	0.997402
WNK1	393.5384	0.199862	1.199381	0.23038	0.997402
CCAR1	368.6148	0.189375	1.136212	0.255868	0.997402
CREB3L3	355.7598	-0.25512	-1.52981	0.126064	0.969144
PIGV	350.8615	-0.03957	-0.23711	0.812569	0.997402
BCL2	353.1418	0.532878	3.191253	0.001417	0.044175
SNRNP40	471.1342	-0.24087	-1.44122	0.149523	0.997402

CD16	370.4333	0.146919	0.878255	0.379806	0.997402
CMIP	367.2344	0.418989	2.503895	0.012283	0.246787
GR1	371.751	-0.03605	-0.21525	0.829576	0.997402
WNT8A	349.388	0.514768	3.073294	0.002117	0.061853
GRP78	347.4858	-0.29315	-1.7492	0.080257	0.783717
SFSWAP	367.7218	0.66852	3.985579	6.73E-05	0.0034
TNK2	362.3292	-0.28753	-1.71397	0.086535	0.808693
MON2	380.4686	-0.09015	-0.53691	0.591328	0.997402
C2TA	356.81	-0.1925	-1.14504	0.252193	0.997402
ARSF	368.8788	-0.60533	-3.60031	0.000318	0.013177
GPBP1	349.7112	0.17342	1.028098	0.303904	0.997402
CD80	343.3311	0.153793	0.911659	0.361948	0.997402
ATF6	341.262	-0.0275	-0.16297	0.870541	0.997402
LTK	347.1212	-0.21813	-1.29259	0.196154	0.997402
WAPL	359.4635	-0.31493	-1.86333	0.062416	0.695693
APPBP2	435.0837	0.293833	1.734521	0.082826	0.790508
SEZ6	355.6498	0.158304	0.934345	0.350126	0.997402
JAK1	387.9435	-0.64916	-3.8284	0.000129	0.005947
MAPK11	352.0808	-0.81377	-4.79504	1.63E-06	0.00013
BICRA	636.7641	3.740921	22.0175	1.96E-107	9.36E-104
CA11	351.4794	0.074857	0.440445	0.659615	0.997402
GSC2	342.0593	0.190912	1.12187	0.261918	0.997402
MTMR1	358.4568	-0.08868	-0.52102	0.602352	0.997402
RNF4	418.2132	-0.04767	-0.2797	0.77971	0.997402
CASP8	341.2739	0.266114	1.559759	0.118817	0.94743
INTS13	356.6269	0.707297	4.144284	3.41E-05	0.001831
TSPAN32	574.7273	-0.32994	-1.93223	0.053331	0.638054
CAPZB	355.8398	-0.18662	-1.09276	0.274498	0.997402
TMEM50A	350.5467	0.613988	3.59429	0.000325	0.01337
TMEM154	336.1735	-0.58038	-3.39613	0.000683	0.025204
CTNNA1	363.3183	0.830634	4.860457	1.17E-06	9.71E-05
SUB1	333.5964	-0.0923	-0.54004	0.589172	0.997402
SMIM3	339.2086	0.315798	1.847123	0.064729	0.708764
MYO1G	402.1833	0.33053	1.933193	0.053212	0.637705
IDH1	332.0037	-0.01774	-0.10372	0.917393	0.997402
NDFIP2	378.1392	1.946715	11.37701	5.44E-30	3.90E-27
ARPC4	338.2193	-0.10443	-0.61006	0.541819	0.997402
VAMP8	349.3651	0.106942	0.623548	0.532924	0.997402
RBBP4	335.1916	0.080334	0.467968	0.639808	0.997402
CD48	325.5254	-0.11713	-0.68173	0.495411	0.997402
PAFAH1B2	337.1518	-0.06609	-0.38454	0.700576	0.997402
CCNG1	327.31	0.000887	0.005158	0.995884	0.999604
RPS3A	331.4633	0.192516	1.119117	0.26309	0.997402
PDAP1	324.8168	-0.08459	-0.49156	0.623028	0.997402
PLIN3	331.8923	-0.21012	-1.22013	0.222417	0.997402
GARS1	345.6687	0.154363	0.896196	0.370148	0.997402
HM13	324.6644	0.104724	0.607695	0.543389	0.997402
RPL36AL	327.8872	0.097276	0.564275	0.572567	0.997402
BCAP31	348.3697	0.260806	1.512742	0.130345	0.976908
SRRM2	336.1221	-0.00922	-0.05346	0.957369	0.997402

AC020656	439.6665	-1.57035	-9.0926	9.67E-20	3.30E-17
MORF4L2	334.8143	-0.35229	-2.03972	0.041378	0.545561
RNH1	324.4114	0.102437	0.592941	0.55322	0.997402
AKAP13	408.7262	-0.06041	-0.34789	0.727921	0.997402
CTSS	388.0805	0.483099	2.780033	0.005435	0.134472
HLA-DPA1	367.0778	0.502788	2.887044	0.003889	0.103114
ARHGAP3(	322.3363	-0.05813	-0.3338	0.738533	0.997402
SNX17	375.2731	-0.22449	-1.28896	0.197413	0.997402
MARCHF6	325.7914	0.198374	1.138228	0.255025	0.997402
SUMF2	318.7347	-0.13132	-0.75345	0.451178	0.997402
SSR3	349.9948	-0.35824	-2.05264	0.040108	0.535705
EIF4H	322.7958	0.081257	0.465392	0.641651	0.997402
TAX1BP1	315.1988	0.466152	2.669114	0.007605	0.173168
UGP2	341.7778	0.548504	3.138696	0.001697	0.051905
CCND3	329.9756	-0.02326	-0.13303	0.894173	0.997402
HEXB	322.0309	-0.08256	-0.47166	0.637172	0.997402
STIP1	312.5237	0.211964	1.210672	0.226021	0.997402
NCOR1	337.804	-0.04947	-0.28197	0.777966	0.997402
PEPD	336.9373	-1.35994	-7.74997	9.19E-15	2.03E-12
STAT3	361.3943	-0.51724	-2.94494	0.00323	0.08911
SLC2A6	316.0133	-0.12085	-0.68787	0.491534	0.997402
SKP1	353.1072	-0.17039	-0.96917	0.332461	0.997402
AP005329.	320.2525	0.274403	1.560559	0.118628	0.946449
QSOX1	359.5601	2.089992	11.8719	1.66E-32	1.58E-29
YWHAE	400.2713	0.060979	0.346376	0.729061	0.997402
RNF130	423.8715	-0.65512	-3.72027	0.000199	0.008704
CD300A	316.9804	-0.20851	-1.18332	0.236681	0.997402
SELENOT	361.8104	0.520478	2.952643	0.003151	0.087545
PSMA3	316.0288	0.42019	2.383406	0.017153	0.310307
SH3GLB1	329.8634	-0.2058	-1.16663	0.243359	0.997402
SPP1	359.6374	0.833789	4.723371	2.32E-06	0.000178
SND1	320.7493	-0.00282	-0.01596	0.987268	0.997464
SELENOF	322.1608	-0.00604	-0.03419	0.972723	0.997402
GNG5	306.6391	0.214787	1.215942	0.224007	0.997402
GNAI3	329.9209	0.106498	0.602842	0.546614	0.997402
DYNLL1	326.0112	0.060319	0.341397	0.732805	0.997402
CALM3	398.8146	-0.04061	-0.22985	0.81821	0.997402
ILF3	316.8889	0.224605	1.270404	0.203941	0.997402
NFATC3	339.3475	1.192375	6.744034	1.54E-11	2.60E-09
TSPO	317.1818	-0.11832	-0.66881	0.503615	0.997402
ST6GAL1	379.5223	0.265235	1.498856	0.133911	0.985105
SON	307.4525	0.244549	1.381373	0.167164	0.997402
PPP2R1A	342.4077	-0.11935	-0.67409	0.500254	0.997402
CCT4	326.1952	0.230576	1.302048	0.1929	0.997402
ERGIC3	304.2772	0.129187	0.728458	0.466333	0.997402
PDLIM5	314.1484	0.127402	0.71837	0.472529	0.997402
ATP5MG	326.5453	0.34625	1.951097	0.051045	0.621075
TGFBI	393.459	2.248093	12.66001	9.85E-37	1.18E-33
FUS	332.5184	-0.03685	-0.20748	0.835637	0.997402
RPS15	349.825	0.166857	0.939085	0.347687	0.997402

SEC31A	301.1069	0.06356	0.357533	0.720693	0.997402
EEF1D	305.3058	0.194421	1.093437	0.274202	0.997402
IL2RG	345.4026	0.731026	4.109924	3.96E-05	0.002095
UBA1	372.6421	-0.27006	-1.5176	0.129116	0.973703
ARL8B	328.2186	0.180558	1.014426	0.31038	0.997402
CDK4	331.1838	-0.49483	-2.77993	0.005437	0.134472
FAU	311.7589	0.139456	0.783073	0.433584	0.997402
MORF4L1	314.5719	-0.32766	-1.83954	0.065836	0.714218
COPZ1	303.3265	-0.00994	-0.05576	0.95553	0.997402
CD99	314.3214	-0.25125	-1.40932	0.158741	0.997402
SPINT2	304.2488	0.706138	3.960087	7.49E-05	0.003732
OAT	317.5264	-0.28104	-1.57504	0.115247	0.933223
CBX5	303.1718	-0.09474	-0.53086	0.595512	0.997402
NUCB1	318.3051	0.090648	0.507468	0.611827	0.997402
SRPRA	296.4877	0.228057	1.275922	0.201983	0.997402
ZNF207	296.7787	-0.03035	-0.1697	0.865246	0.997402
IFNGR1	306.3307	0.276237	1.544217	0.122536	0.957847
EFCAB14	324.041	-0.90371	-5.05073	4.40E-07	3.92E-05
FBXO7	302.4828	0.320057	1.787344	0.073882	0.755128
PAIP2	299.595	-0.04263	-0.23802	0.811865	0.997402
GCSAML	455.3243	0.079605	0.443951	0.657078	0.997402
LCP2	321.9916	0.444115	2.47364	0.013374	0.263539
CASP3	355.6574	1.197881	6.668966	2.58E-11	4.20E-09
FLII	334.991	-0.21608	-1.2026	0.229131	0.997402
DDIT4	339.1194	-0.70548	-3.92432	8.70E-05	0.004248
RPL24	318.662	0.169605	0.943281	0.345537	0.997402
COPB1	300.0873	-0.15807	-0.87909	0.379351	0.997402
HBS1L	293.1027	-0.14304	-0.79538	0.426394	0.997402
SNX5	297.4057	0.129514	0.719871	0.471605	0.997402
ADRB2	358.0497	-1.67032	-9.28391	1.63E-20	6.01E-18
ATP6V0D1	314.0539	-0.10522	-0.58478	0.558698	0.997402
SLC45A3	336.4317	-0.61457	-3.41529	0.000637	0.02374
RPS7	315.545	0.250777	1.393182	0.163565	0.997402
UBA7	296.6028	-0.55999	-3.10966	0.001873	0.056211
HSPD1	292.5555	0.41134	2.284139	0.022363	0.369654
AKR1A1	302.2592	-0.15003	-0.83296	0.40487	0.997402
ATP6V0A2	298.5498	-0.73935	-4.10397	4.06E-05	0.002134
RALB	309.4082	-0.44208	-2.45379	0.014136	0.273876
EIF5	292.3559	0.267639	1.484993	0.137546	0.992942
ARHGAP15	331.4189	-0.49633	-2.75143	0.005934	0.143293
PPP1CA	335.91	0.00782	0.043305	0.965459	0.997402
BEX4	290.3798	-0.4259	-2.35783	0.018382	0.326782
RAB1A	313.5385	0.196627	1.088534	0.276359	0.997402
CRTAP	293.5541	-0.24252	-1.3425	0.179433	0.997402
TUFM	297.5776	0.448542	2.482931	0.013031	0.258183
SHMT2	326.0761	0.13962	0.772727	0.439684	0.997402
SIGLEC17P	310.8337	0.279764	1.54819	0.121577	0.954579
HERC3	300.4583	0.046046	0.254662	0.798984	0.997402
CHCHD2	288.5572	0.096723	0.534712	0.592849	0.997402
ZFP36L1	311.7108	-0.61535	-3.39915	0.000676	0.025044

CSF2RB	458.3288	0.155121	0.856821	0.391544	0.997402
MAP1LC3E	305.7355	0.261629	1.443511	0.148877	0.997402
FDFT1	303.1122	-0.16299	-0.89917	0.368562	0.997402
GRAP2	301.3065	0.590161	3.254989	0.001134	0.037054
HMGN1	287.2832	0.104292	0.575147	0.565192	0.997402
PRDX3	312.2241	0.134457	0.74138	0.458463	0.997402
AC024909.	370.1352	-0.60655	-3.3389	0.000841	0.029501
NDUFA1	286.8546	0.159866	0.878557	0.379641	0.997402
EIF3A	341.0201	-0.07511	-0.41272	0.679815	0.997402
BNIP2	298.3477	-0.20099	-1.10419	0.26951	0.997402
SPCS1	345.855	-0.05478	-0.30089	0.763498	0.997402
POMP	284.3392	0.227932	1.25101	0.210931	0.997402
TARS1	283.7648	0.080242	0.439894	0.660014	0.997402
MTND2P28	307.8423	0.387447	2.122016	0.033836	0.483584
PSMA5	282.2178	0.039475	0.216079	0.828926	0.997402
HDLBP	403.2692	-0.19108	-1.0455	0.29579	0.997402
UBE2L6	295.5815	-0.20558	-1.12473	0.260702	0.997402
CDC42SE1	286.1198	-0.02999	-0.16396	0.869765	0.997402
GTF3A	280.557	0.082864	0.452869	0.650643	0.997402
MYO1F	301.9384	-0.07545	-0.41219	0.680199	0.997402
NEU1	307.6631	0.11521	0.628283	0.529819	0.997402
GSTP1	288.2318	0.139473	0.760117	0.447184	0.997402
PSMA4	280.8166	0.064393	0.350914	0.725653	0.997402
KCNQ1OT1	332.7509	0.09492	0.517159	0.605045	0.997402
OXA1L	320.1583	0.236709	1.288628	0.197527	0.997402
NARS1	288.4864	0.264865	1.441556	0.149428	0.997402
TUBA1C	282.7484	0.075637	0.411564	0.680659	0.997402
EIF3K	281.9985	0.206705	1.12399	0.261017	0.997402
TMED10	345.5133	0.098836	0.537098	0.5912	0.997402
OSBPL3	295.3226	0.802404	4.35872	1.31E-05	0.000849
BSG	322.4034	0.429203	2.331134	0.019746	0.341286
ADPGK	306.9806	0.406215	2.205976	0.027386	0.425606
AL671883.	332.1407	0.022892	0.124234	0.90113	0.997402
SHISA5	298.5225	-0.32697	-1.77192	0.076407	0.761153
PSME2	291.5094	0.433718	2.349259	0.018811	0.331908
KPNA2	275.249	0.27317	1.479319	0.139055	0.997402
ILK	285.5148	-0.20893	-1.13111	0.25801	0.997402
ERAP1	276.2183	-0.28189	-1.52594	0.127026	0.970279
MAPK1IP1	294.0746	0.214177	1.159009	0.246453	0.997402
HMGN2	323.3981	0.007079	0.038288	0.969458	0.997402
CDC37	283.8177	0.305881	1.654258	0.098075	0.859956
SRI	300.7928	0.244782	1.323729	0.185593	0.997402
OSTF1	280.5407	-0.32243	-1.74306	0.081323	0.783898
UQCRH	280.0503	0.465853	2.514093	0.011934	0.241796
KMT2E	277.3796	-0.17123	-0.924	0.355486	0.997402
AC010761.	275.8591	0.070783	0.381842	0.702578	0.997402
GATA2	406.0997	-0.22894	-1.23475	0.216924	0.997402
COX5A	283.8461	0.336378	1.814156	0.069654	0.734154
VPS35	286.8874	-0.25654	-1.38346	0.166523	0.997402
COX7A2	289.4873	0.302605	1.631684	0.102746	0.884158

HNRNPR	331.1037	0.102144	0.550066	0.582274	0.997402
MIF-AS1	270.8478	0.049871	0.268427	0.788371	0.997402
DSTN	278.4246	-0.63475	-3.41608	0.000635	0.023732
DAP	301.4908	-0.01091	-0.05873	0.953171	0.997402
CTNNBL1	286.7547	0.028045	0.150885	0.880066	0.997402
RPL35	304.513	0.163103	0.875119	0.381509	0.997402
IDI1	280.126	-0.31266	-1.67612	0.093714	0.839679
NCF2	295.8424	1.496003	8.019064	1.07E-15	2.59E-13
IDH2	268.4761	-0.08661	-0.46422	0.64249	0.997402
CD14	329.8732	-1.66405	-8.91221	5.00E-19	1.59E-16
AC009690	272.0102	0.457374	2.449274	0.014314	0.274887
TXLNA	268.7046	-0.21406	-1.14625	0.251694	0.997402
NAGA	267.0157	-0.19686	-1.05357	0.292078	0.997402
MDH2	268.7933	0.251566	1.345587	0.178436	0.997402
ABHD2	267.7984	-0.35807	-1.91462	0.055541	0.653964
DHX15	295.599	0.15468	0.826803	0.408349	0.997402
PARP1	292.8428	-0.3296	-1.76177	0.078109	0.773271
HSPA4	266.3083	0.154571	0.825825	0.408903	0.997402
RPS6KA1	271.1396	0.647078	3.455009	0.00055	0.021163
TAOK3	309.5954	-0.71303	-3.80667	0.000141	0.006374
PSMC5	267.0665	-0.1376	-0.7343	0.462766	0.997402
EIF3I	286.0398	-0.0764	-0.40744	0.683683	0.997402
PTP4A1	285.6019	0.076348	0.406712	0.68422	0.997402
DHRS7	266.8334	-0.34347	-1.82902	0.067397	0.724199
APOBEC3C	282.605	-0.05261	-0.27994	0.779523	0.997402
SLC46A3	289.8267	0.352442	1.873915	0.060942	0.689444
PSMB3	263.826	0.250104	1.328548	0.183997	0.997402
GLO1	338.7516	-0.79944	-4.24654	2.17E-05	0.001266
NCKAP1L	295.2626	-0.24611	-1.30721	0.19114	0.997402
DECR1	264.936	-0.31902	-1.69392	0.09028	0.827516
HLA-DRA	405.6919	1.711596	9.085631	1.03E-19	3.44E-17
DNAJC8	268.0083	-0.13605	-0.72197	0.470312	0.997402
ERP44	288.0168	0.21284	1.129437	0.258713	0.997402
GUSB	305.5169	-0.28524	-1.51303	0.130273	0.976908
PIM1	456.6959	-0.63802	-3.38412	0.000714	0.025867
OAZ2	275.5674	-0.77296	-4.09689	4.19E-05	0.002192
MLF2	265.9368	0.114908	0.60899	0.542531	0.997402
DUSP14	298.2939	-1.62505	-8.612	7.18E-18	2.10E-15
ATXN10	295.4106	0.04832	0.25607	0.797897	0.997402
CREB3L2	262.7461	-0.21397	-1.13383	0.256865	0.997402
PRKAR1A	460.3144	-0.28816	-1.52559	0.127111	0.970416
CCT2	281.2057	0.329558	1.744256	0.081114	0.783898
RAB6A	266.7069	-0.11213	-0.59228	0.553664	0.997402
COPG1	313.4865	-0.18543	-0.97833	0.327912	0.997402
PLAUR	269.8306	0.013291	0.070093	0.944119	0.997402
GAS5	276.8398	0.461471	2.43339	0.014958	0.280876
ATP6V1G1	263.0334	0.158605	0.834683	0.403896	0.997402
EZR	261.04	0.464238	2.439138	0.014722	0.278778
RNF213	316.6672	1.364148	7.159451	8.10E-13	1.53E-10
RPL9	270.4807	0.165695	0.869336	0.384663	0.997402

PRRC2C	288.9203	0.126897	0.665521	0.505718	0.997402
ARL6IP1	260.1151	0.283149	1.483997	0.13781	0.993906
GPAA1	274.7136	0.171455	0.898385	0.368981	0.997402
DCTN2	292.1129	-0.44705	-2.34188	0.019187	0.336889
PSMD8	276.0898	0.188171	0.984758	0.324743	0.997402
PIP4K2A	279.7266	-0.13808	-0.72228	0.47012	0.997402
RBM39	272.2166	0.057799	0.302208	0.762493	0.997402
TXN	308.861	0.307565	1.607763	0.107887	0.906643
SYPL1	269.7857	0.093144	0.486772	0.62642	0.997402
AK2	258.6422	0.369765	1.931586	0.053411	0.638385
FAM32A	257.0739	0.259218	1.353945	0.175754	0.997402
NOL4L	259.6396	0.759785	3.965163	7.33E-05	0.003666
N4BP2L2	253.3628	0.212998	1.111505	0.266351	0.997402
MBNL1	257.0642	0.190556	0.994284	0.320085	0.997402
TRAFD1	260.2461	-0.46028	-2.40042	0.016376	0.301948
SCAMP2	263.955	-0.11108	-0.5789	0.56266	0.997402
GBE1	328.0175	-1.43719	-7.4863	7.08E-14	1.39E-11
AARS1	264.8504	-0.56036	-2.9149	0.003558	0.095759
SASH3	252.7715	0.553678	2.878714	0.003993	0.104525
EYA3	255.2106	-0.60485	-3.14466	0.001663	0.050968
PMP22	268.908	-0.37968	-1.97389	0.048394	0.601537
SKAP2	250.1634	0.066292	0.344477	0.730487	0.997402
PSMB6	249.7349	0.316953	1.645945	0.099775	0.868491
PDCD6IP	270.3315	0.051508	0.267359	0.789193	0.997402
PROS1	259.4516	-0.70907	-3.67886	0.000234	0.010062
LAIR1	249.0182	0.032692	0.169554	0.865361	0.997402
DOK3	256.3894	-0.29542	-1.53196	0.125533	0.968243
PRMT2	264.2366	-0.08117	-0.42058	0.674061	0.997402
SLA	264.7804	1.145306	5.928331	3.06E-09	3.85E-07
RGS19	248.2504	0.012234	0.063295	0.949531	0.997402
SERBP1	257.8311	0.324324	1.677818	0.093383	0.839679
ACSL1	312.1674	-0.0714	-0.36921	0.71197	0.997402
KDELR2	251.7678	-0.07435	-0.38442	0.700667	0.997402
PSMB2	250.3327	0.109916	0.568002	0.570034	0.997402
USP11	252.3316	-0.20228	-1.04508	0.295987	0.997402
HDAC2	285.2331	0.190742	0.985284	0.324485	0.997402
SEC11A	296.7249	0.047287	0.244184	0.807088	0.997402
FCER1A	333.6102	1.164189	6.010573	1.85E-09	2.42E-07
MAT2B	258.4847	0.084276	0.434942	0.663605	0.997402
WASF2	249.5244	0.067575	0.348708	0.727309	0.997402
WDR6	263.7155	-0.04026	-0.20762	0.835522	0.997402
CD47	259.4985	0.479177	2.469547	0.013528	0.265479
SOD1	274.3122	0.172673	0.889735	0.373608	0.997402
DDIT4-AS1	252.9875	-0.74072	-3.81476	0.000136	0.00623
THRAP3	247.3047	-0.27099	-1.39429	0.163229	0.997402
TMC6	250.4045	0.191971	0.987702	0.323298	0.997402
BACE2	244.7662	0.125039	0.643296	0.520032	0.997402
VPS28	248.9744	-0.24758	-1.27347	0.202853	0.997402
LAMTOR1	244.127	0.186855	0.960651	0.336728	0.997402
DLD	245.0157	0.265554	1.364449	0.172426	0.997402

ANKRD10	259.3645	-0.07116	-0.36559	0.714673	0.997402
PFKP	251.2089	0.006561	0.033694	0.973121	0.997402
G6PD	261.9315	1.145419	5.882356	4.04E-09	4.96E-07
RAB8A	246.8265	0.211633	1.086533	0.277243	0.997402
ZFAND6	242.2335	-0.05953	-0.30551	0.759977	0.997402
QARS1	274.4591	-0.01094	-0.05612	0.955244	0.997402
CYRIB	281.7006	0.143415	0.735656	0.46194	0.997402
COX5B	263.1249	0.057323	0.294026	0.768738	0.997402
CYBC1	244.9941	0.015144	0.077651	0.938105	0.997402
KLF6	293.2134	-0.50802	-2.60171	0.009276	0.202522
RBBP7	246.1713	-0.18817	-0.96216	0.335968	0.997402
NCOA3	258.6491	0.521166	2.663967	0.007723	0.174731
AP1G2	241.6077	0.147066	0.75163	0.452274	0.997402
COX6C	248.7347	0.058713	0.30003	0.764154	0.997402
CYB5R3	258.0287	-0.21705	-1.10783	0.267934	0.997402
AP1B1	269.2341	0.869126	4.43367	9.26E-06	0.00063
STRAP	245.2955	0.218942	1.116562	0.264182	0.997402
JUP	268.0639	-0.17611	-0.89793	0.369224	0.997402
SF3B3	265.1582	0.004681	0.02386	0.980964	0.997402
RHBDD2	246.4111	-0.24426	-1.24433	0.213376	0.997402
AP1S2	255.419	0.341269	1.737872	0.082233	0.788236
FMNL3	251.7319	-0.1254	-0.63798	0.523487	0.997402
APEH	247.0986	-0.22043	-1.12074	0.2624	0.997402
SHKBP1	241.5359	0.164852	0.837385	0.402376	0.997402
CNPY3	237.6573	-0.1152	-0.58442	0.558935	0.997402
ANAPC5	248.8237	0.145751	0.739407	0.45966	0.997402
STAT1	270.5074	0.098263	0.498275	0.61829	0.997402
DDX39A	236.9871	0.279764	1.418135	0.156151	0.997402
ITGAM	306.1435	0.576301	2.919899	0.003501	0.094592
SPART	249.4175	-0.85304	-4.31934	1.56E-05	0.00097
SWAP70	244.8905	0.165058	0.835734	0.403305	0.997402
TSPYL1	241.26	-0.15025	-0.75966	0.44746	0.997402
CHI3L1	327.8783	-0.9532	-4.81304	1.49E-06	0.00012
SBNO1	234.7024	0.18623	0.940239	0.347095	0.997402
RPL22	252.1374	0.170416	0.860284	0.389632	0.997402
STX3	233.0757	-0.06678	-0.33706	0.736073	0.997402
ADGRE5	247.0955	-0.35692	-1.80077	0.07174	0.745729
TLN1	294.8498	-0.0481	-0.24265	0.808275	0.997402
PSMD6	242.3782	0.282624	1.425613	0.15398	0.997402
C14orf119	242.5801	0.358526	1.808217	0.070573	0.741659
BZW1	240.3494	0.235428	1.187115	0.235182	0.997402
SNX2	245.6219	0.481348	2.425745	0.015277	0.285722
SAR1A	232.3299	-0.08261	-0.41617	0.677283	0.997402
SUMO2	237.8187	-0.00161	-0.00808	0.993552	0.99958
TMEM230	259.3487	-0.00084	-0.00424	0.996615	0.999604
HPS1	232.3442	0.14194	0.713604	0.475472	0.997402
SRSF5	260.5111	-0.08512	-0.42796	0.668682	0.997402
NCBP2	238.5448	0.513894	2.582923	0.009797	0.209752
NDUFS2	233.9064	-0.12707	-0.63835	0.523246	0.997402
KMT2C	257.2951	0.956678	4.804449	1.55E-06	0.000124

BST2	249.6389	-0.23006	-1.15477	0.248187	0.997402
SNX1	236.3842	0.005013	0.025141	0.979943	0.997402
AUP1	239.8557	-0.06847	-0.34324	0.731418	0.997402
SRSF6	238.2809	0.176927	0.886292	0.37546	0.997402
CNIH4	240.6943	0.364727	1.825915	0.067863	0.725567
EFTUD2	260.1958	0.295794	1.480699	0.138687	0.997225
TMEM63A	233.4425	-0.22739	-1.13805	0.255098	0.997402
SLC25A39	239.4466	-0.02052	-0.10267	0.918223	0.997402
CS	240.0678	0.021128	0.105703	0.915818	0.997402
FES	242.8217	0.586814	2.934962	0.003336	0.091418
DDB1	253.3651	0.06304	0.315283	0.752547	0.997402
PPP3CA	235.368	-0.21258	-1.06295	0.287804	0.997402
TRIB2	235.4195	-0.22412	-1.11981	0.262794	0.997402
ALOX5	245.4427	-0.19731	-0.98561	0.324323	0.997402
DDX17	244.2751	-0.11745	-0.58649	0.557547	0.997402
GSTK1	240.7709	0.301873	1.505469	0.132204	0.982113
KCNMB1	292.0859	2.101003	10.47287	1.15E-25	6.11E-23
ABRACL	233.5223	-0.65629	-3.27107	0.001071	0.03566
AKAP12	253.9341	0.310388	1.54654	0.121974	0.955088
SCP2	239.5587	0.021856	0.108864	0.91331	0.997402
HMGB1	228.5845	0.255861	1.272566	0.203172	0.997402
HIPK2	227.4969	0.401047	1.993759	0.046178	0.583637
DNAJB1	228.6632	-0.28618	-1.42247	0.15489	0.997402
EMP3	229.1195	-0.48532	-2.41228	0.015853	0.293818
CNDP2	232.7115	0.295525	1.468829	0.141879	0.997402
MTCH2	224.5653	0.221584	1.099883	0.271383	0.997402
PDCD4	236.0016	-0.93337	-4.63197	3.62E-06	0.000265
PSTPIP2	232.4121	-0.27266	-1.35243	0.176238	0.997402
WSB1	269.2617	-0.06039	-0.29949	0.764563	0.997402
CSNK1A1	226.6311	-0.1402	-0.69523	0.486913	0.997402
CLINT1	241.2491	0.049948	0.247593	0.804449	0.997402
STMP1	226.5806	-0.32045	-1.58838	0.112201	0.923117
PSMD13	241.7759	0.007492	0.037124	0.970386	0.997402
AP3S1	229.8148	-0.55156	-2.73242	0.006287	0.150819
VAMP3	226.9092	-0.02836	-0.14047	0.888287	0.997402
RPS2	223.3583	0.042839	0.212172	0.831973	0.997402
INPP5D	261.996	-0.46354	-2.2951	0.021728	0.364113
TPD52L2	227.1897	-0.36733	-1.81855	0.06898	0.729765
FCN1	229.5838	-0.18298	-0.90559	0.365155	0.997402
AGBL5	227.507	-0.2737	-1.35428	0.175648	0.997402
BHLHE40	341.1602	-0.42819	-2.11867	0.034119	0.485068
RSU1	224.4786	-0.07529	-0.37244	0.709568	0.997402
CYTH4	222.5988	-0.1172	-0.57934	0.562357	0.997402
SEC13	235.8606	-0.12601	-0.62264	0.533522	0.997402
LIMS1	230.4444	-0.116	-0.5728	0.566777	0.997402
ADIPOR1	237.1751	-0.17787	-0.8783	0.379782	0.997402
CDKN1A	254.4625	1.631458	8.051464	8.18E-16	2.02E-13
TNFRSF14	259.3338	0.628542	3.101026	0.001929	0.057755
TBC1D9B	322.4966	-0.36146	-1.78285	0.074611	0.755855
TANC1	224.9764	-0.10052	-0.49544	0.620288	0.997402

MRFAP1	321.3512	-0.21001	-1.03476	0.300783	0.997402
TINF2	225.8991	-0.24115	-1.18796	0.23485	0.997402
SRP9	227.1073	0.231367	1.139201	0.254619	0.997402
AC245140	248.0324	0.119646	0.588315	0.556321	0.997402
AP2B1	233.8632	-0.31255	-1.53681	0.124339	0.964132
NUCB2	243.2908	1.262486	6.205007	5.47E-10	7.62E-08
TAF7	227.1948	-0.12176	-0.59823	0.549689	0.997402
PRPF40A	232.78	0.11856	0.581516	0.560892	0.997402
LPCAT3	222.8292	-0.28372	-1.39148	0.164081	0.997402
FOXP1	230.2784	-0.07607	-0.373	0.709148	0.997402
GNAS	226.798	-0.0465	-0.22761	0.819948	0.997402
DNAJC3	217.8283	0.030207	0.147807	0.882495	0.997402
PHB2	235.5819	0.424062	2.074017	0.038078	0.517919
ACO2	218.1373	0.053221	0.260205	0.794706	0.997402
DEGS1	230.4571	-0.07307	-0.35712	0.721005	0.997402
UBR4	260.7341	0.048882	0.238894	0.811188	0.997402
ACTR1A	250.9911	0.026314	0.12858	0.89769	0.997402
SARS1	232.0781	0.051033	0.249264	0.803157	0.997402
RBPJ	225.05	0.803487	3.923563	8.72E-05	0.004248
YTHDF2	223.6754	0.188331	0.91965	0.357755	0.997402
PUF60	240.1289	0.238194	1.160511	0.245841	0.997402
XRN2	258.7719	-0.32405	-1.57881	0.114379	0.930141
ABCC4	243.1359	0.597253	2.907577	0.003642	0.097482
MRPS6	255.8163	-0.10166	-0.49484	0.620712	0.997402
RTRAF	215.8156	0.132203	0.643461	0.519925	0.997402
PPP1R15A	239.0122	-0.2091	-1.01774	0.308804	0.997402
APEX1	283.2759	-0.03518	-0.17122	0.86405	0.997402
CCNDBP1	231.8703	-0.02211	-0.10759	0.914321	0.997402
ATP6V1E1	216.576	-0.07318	-0.35597	0.721864	0.997402
OSBPL9	229.6529	-0.51134	-2.48552	0.012936	0.256664
VPS29	217.7019	-0.17465	-0.84893	0.395921	0.997402
ACLY	307.8415	0.087362	0.424003	0.671564	0.997402
CISH	267.0042	2.418171	11.72247	9.78E-32	8.25E-29
CAT	250.6418	0.058894	0.285418	0.775324	0.997402
COPS3	219.4102	0.429319	2.07868	0.037647	0.516145
CCNA1	277.0336	-2.03359	-9.84215	7.41E-23	3.32E-20
VDAC3	233.6123	0.043269	0.209188	0.834301	0.997402
CYTIP	224.1512	-1.28458	-6.20658	5.42E-10	7.62E-08
CD55	239.1476	-0.32342	-1.56236	0.118204	0.944642
RPL21	213.6463	0.080349	0.388048	0.697981	0.997402
TFG	255.6799	-0.12479	-0.60259	0.54678	0.997402
PFKL	226.0958	-0.19201	-0.92702	0.353918	0.997402
MAPRE2	214.3649	-0.45486	-2.19584	0.028103	0.430249
PSMB8	232.0251	0.094816	0.457649	0.647204	0.997402
PSMD4	215.1126	0.121627	0.587052	0.557169	0.997402
AIF1	219.0028	-0.32578	-1.57194	0.115963	0.936653
ATF4	213.9895	-0.03962	-0.19115	0.848411	0.997402
HCK	224.7529	0.08312	0.400901	0.688493	0.997402
CD46	311.4433	0.076324	0.367931	0.712925	0.997402
UQCRB	222.6291	0.335049	1.613916	0.106546	0.900642

FGR	220.6223	0.782628	3.769848	0.000163	0.007239
STAP1	231.1245	-0.37857	-1.82134	0.068555	0.729082
BLVRA	216.3688	-0.46495	-2.23623	0.025337	0.403685
ELOVL1	256.6164	-0.04443	-0.21359	0.830865	0.997402
KEAP1	213.913	-0.03086	-0.14822	0.882168	0.997402
PLCB2	224.87	0.367362	1.764439	0.077658	0.76934
HEXA	210.241	0.219134	1.052303	0.292661	0.997402
PSMD7	218.5386	0.15982	0.76747	0.442802	0.997402
CYBB	265.6614	-0.19723	-0.94619	0.344053	0.997402
ARFGAP2	231.6085	-0.18764	-0.89931	0.36849	0.997402
DOCK2	231.0481	-0.20436	-0.97947	0.327349	0.997402
MLPH	242.5467	-1.51292	-7.24944	4.19E-13	8.00E-11
CDS2	212.6983	-0.0502	-0.24032	0.810082	0.997402
ATP6V1A	246.8365	0.267891	1.282025	0.199834	0.997402
TMEM176	224.7437	-0.22242	-1.06435	0.287171	0.997402
ABI1	207.3593	-0.08839	-0.4229	0.672366	0.997402
TTYH2	223.6973	0.219089	1.047366	0.294931	0.997402
PSMC3	224.5154	0.149539	0.714788	0.47474	0.997402
OSTC	220.2671	-0.02282	-0.109	0.913202	0.997402
PLP2	221.4199	0.173612	0.829176	0.407005	0.997402
CTR9	245.5927	-0.73329	-3.5017	0.000462	0.018119
DDX6	224.994	-0.21237	-1.01397	0.310598	0.997402
HGS	239.0184	0.08609	0.410789	0.681227	0.997402
HNRNPH3	209.5188	-0.05593	-0.26667	0.789726	0.997402
CASP2	221.3367	0.346525	1.651664	0.098603	0.862477
RNF7	209.1476	-0.25259	-1.20394	0.228614	0.997402
NABP1	215.0499	0.224883	1.071651	0.283877	0.997402
TMEM273	234.2194	1.806234	8.606746	7.52E-18	2.16E-15
USP10	232.3214	0.10407	0.495853	0.619998	0.997402
DR1	212.3727	0.042657	0.203194	0.838983	0.997402
MAGED2	258.8492	-0.19254	-0.91678	0.359256	0.997402
ELMO1	217.3671	-0.49554	-2.3578	0.018384	0.326782
MOB3A	231.7041	0.300169	1.427941	0.153309	0.997402
FUCA2	209.8893	0.077916	0.370425	0.711066	0.997402
TSG101	209.7411	0.010845	0.051551	0.958886	0.997402
LAMTOR5	207.8609	0.100085	0.475718	0.634275	0.997402
POLR2A	226.5157	0.403744	1.918264	0.055078	0.651349
UBL5	204.2658	0.224884	1.068049	0.285498	0.997402
LIF	239.0095	0.832219	3.95023	7.81E-05	0.003875
EDF1	203.0485	-0.12275	-0.58225	0.5604	0.997402
SRSF7	204.0695	0.320458	1.519982	0.128516	0.972853
ATP6V1C2	204.4188	0.207199	0.981919	0.32614	0.997402
PSMC2	207.0756	0.336913	1.596227	0.110438	0.919407
ARAP1	212.432	-0.25299	-1.19845	0.23074	0.997402
C4orf3	208.7591	0.017418	0.082373	0.93435	0.997402
ESD	211.1713	0.031815	0.150427	0.880428	0.997402
CNP	213.7381	-0.13911	-0.65771	0.510725	0.997402
SNU13	212.413	0.132047	0.623274	0.533105	0.997402
BECN1	210.0273	0.027302	0.128339	0.897881	0.997402
FLOT2	199.1622	-0.27379	-1.28689	0.198133	0.997402

IL3RA	254.5822	2.367067	11.12347	9.64E-29	5.76E-26
FYTTD1	203.0672	-0.0795	-0.37345	0.708815	0.997402
CCND2	203.3296	-0.24131	-1.13337	0.25706	0.997402
ERP29	200.758	0.038756	0.181979	0.855599	0.997402
CD33	208.46	1.081385	5.074705	3.88E-07	3.50E-05
VTA1	201.762	0.291648	1.368447	0.171172	0.997402
HDAC1	210.6144	-0.11689	-0.54842	0.583405	0.997402
UFC1	203.2091	0.067582	0.316702	0.75147	0.997402
COPS6	204.0126	-0.12793	-0.59945	0.54887	0.997402
TNIK	211.5887	-1.36437	-6.39236	1.63E-10	2.39E-08
PPA1	206.1259	0.01293	0.060579	0.951695	0.997402
RTCB	198.6726	-0.03291	-0.15391	0.877681	0.997402
UBA3	197.1129	0.25625	1.19763	0.231061	0.997402
SF3A3	200.6834	0.077619	0.362734	0.716804	0.997402
DHCR24	202.5194	-0.50791	-2.37114	0.017733	0.317582
TMEM33	203.1018	-0.22314	-1.04085	0.297947	0.997402
RPS27L	203.8094	0.096273	0.448925	0.653486	0.997402
ZC3HAV1	205.9034	0.458158	2.13616	0.032666	0.472379
GALNT6	214.5678	-0.06901	-0.32159	0.74776	0.997402
CIRBP	223.0475	0.162284	0.755982	0.44966	0.997402
RBM5	194.4143	-0.15666	-0.72894	0.466039	0.997402
C1QBP	206.447	0.526899	2.451395	0.01423	0.274375
SUPT20H	202.3516	-0.63272	-2.9426	0.003255	0.089612
CD163	218.8077	-0.57326	-2.66508	0.007697	0.174427
HNRNPH2	206.2531	-0.24334	-1.13012	0.258424	0.997402
MAPKAPK3	204.7239	-0.04628	-0.21487	0.829866	0.997402
IMPDH2	211.2963	0.085402	0.396286	0.691894	0.997402
LRRC41	207.5873	-0.12732	-0.59056	0.554812	0.997402
TMEM123	202.7063	0.091528	0.424292	0.671353	0.997402
TXN2	195.0591	0.042228	0.195749	0.844806	0.997402
SNRPD2	200.4515	-0.14571	-0.67533	0.499463	0.997402
HCST	192.6358	0.064321	0.297958	0.765735	0.997402
SEC61B	195.1874	0.167725	0.776891	0.437223	0.997402
TPST2	205.0514	-0.92477	-4.28239	1.85E-05	0.001114
NT5C2	200.722	0.302994	1.402951	0.160631	0.997402
PSMB4	203.7526	-0.08922	-0.41277	0.679774	0.997402
EIF4G1	193.2092	-0.08672	-0.40109	0.68835	0.997402
DDX1	200.635	-0.09741	-0.45018	0.652582	0.997402
SRSF1	208.2494	0.31317	1.447125	0.147862	0.997402
EIF3G	213.1016	0.312608	1.444398	0.148627	0.997402
ADGRE2	217.0693	-1.21005	-5.58587	2.33E-08	2.55E-06
CSRP1	210.4461	-0.45761	-2.11123	0.034752	0.488345
SFXN3	207.3477	-0.43658	-2.01357	0.044054	0.565348
BIN2	202.8379	-0.48165	-2.21943	0.026457	0.417067
IMMT	196.6043	0.256338	1.181069	0.237575	0.997402
RNPS1	194.2038	0.092737	0.427005	0.669376	0.997402
DNAJC7	199.9755	0.29033	1.335003	0.181875	0.997402
TENT5A	204.1916	1.346449	6.19024	6.01E-10	8.21E-08
STK10	205.8414	0.243019	1.116566	0.26418	0.997402
SLC11A2	244.174	0.129382	0.594163	0.552403	0.997402

C11orf58	241.5741	-0.01336	-0.06132	0.951103	0.997402
PCBP1	195.7435	-0.10887	-0.49961	0.617349	0.997402
CEACAM8	225.1006	-0.92184	-4.23008	2.34E-05	0.001335
UQCRQ	225.5419	-0.18773	-0.86113	0.389164	0.997402
DAP3	192.4242	0.371377	1.703545	0.088466	0.818211
MLX	187.8145	0.15609	0.715928	0.474036	0.997402
TMEM9B	192.6983	-0.24686	-1.13195	0.257657	0.997402
RTF2	190.4269	-0.09257	-0.42446	0.671228	0.997402
CALB2	224.8692	-1.46093	-6.69647	2.14E-11	3.52E-09
RNF4	198.6422	0.164473	0.753637	0.451067	0.997402
COMMD6	189.0024	-0.04804	-0.22013	0.82577	0.997402
DUSP10	206.1626	-0.82744	-3.79114	0.00015	0.006722
STOM	191.5697	-0.6902	-3.16228	0.001565	0.048395
RPS6KA5	223.2546	-0.69929	-3.20288	0.001361	0.04273
CDC42SE2	189.8469	-0.30604	-1.39903	0.161805	0.997402
VAMP7	200.7642	0.295275	1.349713	0.177108	0.997402
KARS1	189.1912	0.125442	0.573376	0.56639	0.997402
PPM1G	206.5336	0.211298	0.965312	0.334389	0.997402
MYADM	205.0986	0.103771	0.473955	0.635532	0.997402
EIF4EBP2	197.2148	-0.20269	-0.92543	0.354742	0.997402
SEC24C	200.848	0.293658	1.340537	0.180071	0.997402
SDHB	212.8653	0.512332	2.338724	0.01935	0.338502
PSMD11	217.7064	0.204792	0.934604	0.349992	0.997402
CERS2	198.0106	-0.16881	-0.7698	0.44142	0.997402
APRT	192.3905	0.158568	0.722418	0.470038	0.997402
TSN	185.9579	-0.13471	-0.6135	0.539547	0.997402
IAH1	192.5158	-0.04019	-0.18301	0.854793	0.997402
ATRX	195.7243	0.241533	1.09984	0.271402	0.997402
KIF5B	213.4537	0.224616	1.022725	0.306438	0.997402
UBAP2L	218.989	0.114662	0.521892	0.601745	0.997402
RGS10	197.2747	-0.30168	-1.37306	0.169735	0.997402
PARVG	191.6277	-0.36878	-1.67839	0.093272	0.839679
PIGS	188.4346	0.09876	0.449356	0.653175	0.997402
NAGK	192.001	-0.33642	-1.52985	0.126053	0.969144
EIF2A	195.3846	0.143202	0.651125	0.514966	0.997402
ATRAID	189.6405	0.096042	0.436615	0.662391	0.997402
SERPINB6	190.2607	-0.19624	-0.89137	0.372729	0.997402
CDC123	187.2944	0.000717	0.003256	0.997402	0.999604
RPA1	185.3833	-0.19655	-0.89204	0.372373	0.997402
HPRT1	198.4688	-0.48149	-2.18477	0.028906	0.436934
COX7B	194.3026	0.212836	0.965518	0.334285	0.997402
CTTNBP2	214.868	0.086399	0.391706	0.695275	0.997402
NCF4	193.6103	-0.06304	-0.28576	0.775062	0.997402
NDUFB9	187.3541	0.015069	0.06831	0.945539	0.997402
KRT19	210.2633	-0.94934	-4.30352	1.68E-05	0.001031
AL157871.	184.8036	-0.15607	-0.70739	0.479323	0.997402
SFT2D2	214.334	0.119175	0.540094	0.589132	0.997402
MPG	196.9056	-0.17047	-0.77243	0.43986	0.997402
POLR2B	225.069	0.054853	0.248481	0.803762	0.997402
AC135457	184.5957	-0.00423	-0.01916	0.984717	0.997402

PEF1	191.1333	-0.06522	-0.2954	0.76769	0.997402
PSMB7	183.5591	0.139587	0.632214	0.527247	0.997402
MAVS	183.8221	-0.28537	-1.29224	0.196276	0.997402
SF3B1	345.7612	-0.06933	-0.31375	0.753708	0.997402
MFNG	213.5026	-0.02154	-0.09739	0.922416	0.997402
YIPF3	198.8991	-0.23487	-1.06162	0.288406	0.997402
USP4	191.6744	0.042757	0.19321	0.846795	0.997402
PSMF1	195.8121	-0.33295	-1.50394	0.132596	0.983401
FXR1	195.4811	-0.00016	-0.00074	0.999411	0.99976
PHIP	186.729	-0.01315	-0.05927	0.952735	0.997402
CASP1	183.8388	0.150039	0.676495	0.498726	0.997402
RPL18A	221.4529	-0.00523	-0.02359	0.981183	0.997402
GLA	181.6883	-0.19786	-0.89157	0.372622	0.997402
KPNA6	190.1064	0.15706	0.707631	0.479175	0.997402
SGK1	187.3089	-0.78536	-3.53631	0.000406	0.016123
IVNS1ABP	193.6586	-0.08523	-0.38369	0.701206	0.997402
PSME3	229.0741	0.195636	0.880429	0.378627	0.997402
IL2RA	191.4094	-0.92577	-4.16405	3.13E-05	0.001718
CTNNB1	195.6167	0.170266	0.765496	0.443976	0.997402
ELF1	183.5342	-0.31262	-1.40538	0.159909	0.997402
PDXDC1	185.9546	0.36701	1.649621	0.099021	0.8656
SFT2D1	183.3503	0.151523	0.680863	0.495958	0.997402
YARS1	201.0014	-0.00178	-0.00801	0.993613	0.99958
PAG1	197.5384	0.050693	0.227649	0.819919	0.997402
GIT2	186.9111	-0.10069	-0.45214	0.651165	0.997402
SIGLEC8	188.0397	-0.20263	-0.90986	0.362898	0.997402
AMD1	234.0926	0.398323	1.78837	0.073716	0.755056
PRDX5	198.4173	-0.33176	-1.48942	0.136376	0.990539
SRSF10	184.7248	0.191575	0.860047	0.389763	0.997402
CCAR1	180.4808	-0.17963	-0.80642	0.42	0.997402
ARF4	187.2104	-0.03616	-0.16228	0.871085	0.997402
MAP2K3	181.702	-0.19768	-0.88682	0.375173	0.997402
CRBN	181.5908	-0.52227	-2.34269	0.019145	0.336567
EIF1B	199.2595	1.105635	4.958581	7.10E-07	6.11E-05
CD38	188.3847	1.207197	5.41309	6.19E-08	6.49E-06
SNX6	204.6897	0.035051	0.157154	0.875123	0.997402
SOX4	187.9997	-0.01707	-0.07652	0.939005	0.997402
CYCS	179.7233	0.483176	2.166161	0.030299	0.447696
TXNL1	185.6194	0.081768	0.366552	0.713953	0.997402
DCAF7	196.4764	0.110022	0.493063	0.621968	0.997402
TCF25	178.4489	-0.04258	-0.19065	0.848802	0.997402
ENG	179.4669	0.447007	2.001245	0.045366	0.576928
CMA1	189.2912	-0.27672	-1.23868	0.215464	0.997402
POLDIP3	178.1419	0.150028	0.671457	0.501929	0.997402
IL9R	188.6798	1.264284	5.655845	1.55E-08	1.72E-06
ACTR10	189.981	-0.2385	-1.06689	0.286024	0.997402
TMCO1	182.1377	-0.05977	-0.26736	0.789196	0.997402
AC008894	189.5518	0.312758	1.398311	0.16202	0.997402
PNN	179.9257	0.241437	1.079298	0.280455	0.997402
SEMA4A	181.5696	0.029144	0.130252	0.896367	0.997402

SCAF11	191.6682	-0.32312	-1.44412	0.148705	0.997402
AC092069.	198.2266	-0.08466	-0.37831	0.705199	0.997402
FUBP1	186.6203	-0.01059	-0.04729	0.962284	0.997402
CNOT1	226.6585	0.105229	0.469704	0.638567	0.997402
MITF	201.5958	-0.9503	-4.24085	2.23E-05	0.001292
ADAMTS14	201.0667	0.393757	1.757111	0.078899	0.776805
EBP	194.0186	-0.72883	-3.25165	0.001147	0.037328
TSPAN14	241.8306	-0.25781	-1.14998	0.25015	0.997402
EMG1	184.7774	-0.04549	-0.20281	0.839285	0.997402
PSMD14	188.135	0.360423	1.606913	0.108073	0.907677
AC114271.	188.7299	0.478215	2.131933	0.033012	0.475155
NOP56	194.7693	0.536484	2.391654	0.016773	0.305722
SNX27	178.7724	0.114397	0.509856	0.610152	0.997402
AL121985.	188.9309	-0.20733	-0.92398	0.355497	0.997402
ACP1	191.7081	0.198774	0.885805	0.375722	0.997402
RBM8A	187.8079	-0.0311	-0.13842	0.88991	0.997402
PLPP1	259.5831	1.149362	5.115788	3.12E-07	2.91E-05
CIAO1	179.8081	-0.05647	-0.25125	0.80162	0.997402
AHSA1	183.8735	-0.2152	-0.95749	0.338321	0.997402
MMP9	211.5095	1.977483	8.795718	1.42E-18	4.43E-16
LBH	180.0034	0.020788	0.092458	0.926334	0.997402
ARL4A	185.2884	0.615004	2.734747	0.006243	0.150005
GSS	175.2832	0.024939	0.11089	0.911704	0.997402
PAK1	176.1955	-0.25501	-1.13325	0.25711	0.997402
UBXN1	182.5992	-0.13033	-0.57902	0.562578	0.997402
WBP2	198.0888	-0.25382	-1.12703	0.25973	0.997402
MANBA	176.1025	-0.40353	-1.79145	0.073222	0.752411
PLA2G7	182.3138	1.022676	4.533032	5.81E-06	0.000403
PIAS1	187.1356	-0.38023	-1.68521	0.091947	0.832177
PHB	176.6541	0.150109	0.66505	0.506018	0.997402
PRPF40B	188.2342	-0.17608	-0.77993	0.435434	0.997402
SLC48A1	177.0651	-0.08708	-0.38566	0.699752	0.997402
MTRNR2L1	203.4162	0.1069	0.473017	0.636201	0.997402
ARIH2	176.2585	0.241074	1.065557	0.286624	0.997402
CNOT9	194.3309	-0.13955	-0.61667	0.537452	0.997402
ARHGEF2	174.8124	0.05003	0.220979	0.825109	0.997402
SSBP1	179.1436	0.262873	1.160876	0.245692	0.997402
IQGAP1	187.392	-0.47632	-2.10247	0.035512	0.49458
CYFIP2	173.5611	-0.24792	-1.09418	0.273878	0.997402
TOMM7	176.7068	0.109158	0.481764	0.629973	0.997402
FBXL5	182.2412	0.042672	0.188308	0.850636	0.997402
TBL1XR1	181.2253	0.32433	1.431066	0.152411	0.997402
TRMT112	173.5555	-0.15583	-0.68742	0.491821	0.997402
ST13	177.8716	0.032685	0.144179	0.885359	0.997402
CYB561A3	173.8848	-0.06913	-0.30476	0.760547	0.997402
CSF1R	239.4403	0.998994	4.401426	1.08E-05	0.000718
LSM4	178.6574	0.494307	2.177697	0.029429	0.441456
CYSTM1	178.6105	-0.45587	-2.00671	0.044781	0.571169
TMED4	184.7064	-0.03755	-0.16528	0.868721	0.997402
TMED3	172.1618	0.078245	0.344264	0.730648	0.997402

PLEKHO2	199.3891	0.1087	0.478218	0.632495	0.997402
NOP10	188.385	0.154426	0.679089	0.497081	0.997402
CD276	174.8894	0.390423	1.715096	0.086328	0.808693
ARMC8	187.7933	0.04196	0.18431	0.85377	0.997402
NAP1L4	173.7469	-0.09967	-0.43777	0.661551	0.997402
ATXN7L3B	192.7425	0.353077	1.549948	0.121154	0.952899
SCIN	225.0376	-1.99571	-8.75366	2.07E-18	6.30E-16
MRPL37	170.9466	0.14115	0.618926	0.535965	0.997402
SS18	183.2262	-0.04401	-0.19286	0.847065	0.997402
C5orf22	212.5546	-0.12742	-0.55818	0.576719	0.997402
CLTA	265.9169	-0.25923	-1.13551	0.256161	0.997402
MMADHC	171.7399	0.452101	1.979186	0.047795	0.596711
PCBP1-AS1	190.4199	0.05161	0.225805	0.821353	0.997402
BNIP3L	180.2497	-0.45286	-1.98102	0.047589	0.595692
MKNK1	170.8334	0.497043	2.172196	0.029841	0.444054
CHTF8	169.6267	-0.13527	-0.59103	0.554499	0.997402
COX6B1	203.7466	-0.02411	-0.10527	0.91616	0.997402
GAPT	184.8499	-0.1194	-0.52127	0.602179	0.997402
HSBP1	169.1333	0.295535	1.29004	0.197037	0.997402
SNHG5	183.5556	0.153698	0.670839	0.502323	0.997402
CEACAM1	213.0209	0.362952	1.58347	0.113314	0.926736
ZC3H13	172.2252	-0.32176	-1.40364	0.160427	0.997402
DCTN5	168.6611	0.044837	0.195524	0.844983	0.997402
UBLCP1	172.8525	-0.25427	-1.10836	0.267708	0.997402
HAVCR2	170.9798	0.004721	0.020576	0.983584	0.997402
DKC1	174.6786	0.250804	1.092902	0.274437	0.997402
B4GALT1	176.9366	-0.03399	-0.14788	0.882439	0.997402
HERPUD1	184.3988	-0.21303	-0.92638	0.35425	0.997402
WNK1	172.0981	-0.53476	-2.32378	0.020137	0.345534
PPP1R12A	185.0975	0.104424	0.453529	0.650168	0.997402
KLRG1	171.2515	-0.32413	-1.40767	0.159228	0.997402
KDM5A	170.5661	-0.07255	-0.31505	0.752726	0.997402
EPB41L1	172.048	-0.8109	-3.52009	0.000431	0.017048
RIC8A	168.2221	-0.15473	-0.67166	0.501799	0.997402
RAF1	170.6898	-0.04122	-0.17892	0.857997	0.997402
PDCD6	167.0432	0.186093	0.807645	0.419295	0.997402
RBM6	170.0596	-0.18472	-0.80167	0.422742	0.997402
PITPNB	174.7273	0.584031	2.534569	0.011259	0.231381
RAD21	170.6529	0.65983	2.862973	0.004197	0.108281
PSMB5	182.5623	0.154231	0.669048	0.503465	0.997402
MAN2B2	172.027	-0.13041	-0.56571	0.57159	0.997402
MDM4	180.2742	-0.17536	-0.76066	0.446858	0.997402
ACTR2	222.5053	-0.2183	-0.9467	0.343789	0.997402
AHCY	181.7531	1.063174	4.60935	4.04E-06	0.000285
PRKDC	169.9053	0.401015	1.737881	0.082232	0.788236
CALCOCO2	170.2481	-0.14421	-0.6249	0.532037	0.997402
PAQR5	172.0624	-0.99752	-4.3217	1.55E-05	0.000966
GPS1	168.1374	-0.17948	-0.77751	0.436859	0.997402
ADI1	181.797	-0.08091	-0.35049	0.725968	0.997402
EIF4E2	166.441	-0.10006	-0.4332	0.664871	0.997402

ATP5IF1	169.9992	-0.35688	-1.54398	0.122594	0.957847
COMT	164.8452	-0.01986	-0.0859	0.931542	0.997402
SP100	167.3646	0.040501	0.175104	0.860998	0.997402
STAU1	185.5947	-0.51048	-2.20506	0.02745	0.425701
SNX20	219.6826	1.006976	4.3479	1.37E-05	0.000888
FARSA	172.3202	0.370889	1.600765	0.109429	0.915312
C11orf24	192.4952	-1.18162	-5.09907	3.41E-07	3.12E-05
TOR1AIP1	165.804	0.201052	0.867436	0.385703	0.997402
SAP18	164.2568	0.11452	0.493996	0.621309	0.997402
RDH11	167.2532	0.200255	0.863663	0.387773	0.997402
IFI16	164.1758	-0.20341	-0.87707	0.380451	0.997402
AC109460	166.8639	-0.4151	-1.78963	0.073514	0.753795
LEPROTL1	165.4591	0.135815	0.585519	0.558199	0.997402
NUDC	179.0558	0.116346	0.50129	0.616167	0.997402
ATP2A3	164.5785	-0.31683	-1.36491	0.172283	0.997402
NDUFB3	174.0457	0.195753	0.84321	0.399111	0.997402
POLR2E	175.917	0.294809	1.26962	0.20422	0.997402
RER1	166.0444	-0.13671	-0.58823	0.556376	0.997402
AP1M1	184.6779	-0.08168	-0.35123	0.725413	0.997402
SAFB	167.8932	-0.07262	-0.31224	0.754857	0.997402
PRKACB	164.7841	-0.59146	-2.54283	0.010996	0.227612
IDH3B	163.5815	0.17142	0.736877	0.461197	0.997402
RSL1D1	176.3056	0.532102	2.287287	0.022179	0.368666
ATG3	165.0084	0.375512	1.613637	0.106606	0.900642
DCAF13	163.8658	-0.26034	-1.11847	0.263365	0.997402
HINT1	173.9857	0.315731	1.356121	0.175061	0.997402
ATP6V0A1	180.5982	-0.24455	-1.05027	0.293596	0.997402
AC008750	166.7628	-0.8619	-3.70077	0.000215	0.009316
STK26	177.3954	0.000308	0.001323	0.998944	0.999695
TSPAN31	189.5645	-0.45128	-1.93702	0.052743	0.633133
GLMP	167.8029	-0.37481	-1.60863	0.107697	0.90656
SEC23B	186.9958	0.415496	1.783244	0.074547	0.755855
PDHB	176.2777	0.19772	0.848121	0.39637	0.997402
RPS26	169.0767	0.072193	0.309651	0.756826	0.997402
GOLGB1	188.9376	1.880479	8.061316	7.55E-16	1.93E-13
MCFD2	183.2573	-0.37989	-1.62835	0.103451	0.885974
GNG2	169.3968	-0.99173	-4.2487	2.15E-05	0.001264
DHRS9	183.2724	-1.51188	-6.47638	9.39E-11	1.42E-08
TOX4	162.0642	0.182256	0.780704	0.434977	0.997402
GMFG	161.3866	-0.04838	-0.20686	0.836117	0.997402
GART	163.6547	0.35726	1.527304	0.126685	0.969929
PGM1	160.7593	-0.14623	-0.62486	0.532065	0.997402
CNOT7	162.7575	0.150243	0.641982	0.520885	0.997402
TUBGCP2	176.4941	0.095588	0.40835	0.683017	0.997402
TNFAIP8	173.0807	-0.35094	-1.49849	0.134007	0.985306
ORMDL1	161.8305	0.413893	1.766763	0.077268	0.768056
NDUFV1	160.387	0.241655	1.03136	0.302372	0.997402
MAP4	178.4264	-0.01688	-0.07205	0.942564	0.997402
GCA	164.8688	-0.19788	-0.84428	0.398512	0.997402
PKIB	178.0066	-0.56707	-2.41818	0.015598	0.290217

TOMM20	185.1049	0.131962	0.562437	0.573818	0.997402
SNRNP200	166.6929	0.071592	0.305106	0.760286	0.997402
TXNDC12	161.7189	0.133684	0.569535	0.568993	0.997402
NRDC	183.7323	-0.16266	-0.69291	0.488366	0.997402
CHMP2A	167.2519	-0.19269	-0.8207	0.411817	0.997402
CHD4	170.8325	-0.04221	-0.17972	0.857371	0.997402
MRPS16	163.7297	0.13351	0.568374	0.569781	0.997402
PSMD2	189.3825	-0.77073	-3.28085	0.001035	0.034688
BRD2	163.482	0.290506	1.236297	0.216348	0.997402
RAB18	163.4547	-0.08454	-0.35976	0.719028	0.997402
FADS1	197.1249	-0.88588	-3.7696	0.000164	0.007239
CD4	180.018	0.323067	1.374651	0.16924	0.997402
XPNPEP1	166.3791	0.162487	0.691352	0.489345	0.997402
ETFA	168.9083	0.187933	0.799563	0.423964	0.997402
AC125611	159.3292	0.143382	0.609865	0.541952	0.997402
EXOSC8	165.8276	-0.61489	-2.61404	0.008948	0.196869
AGTRAP	166.0454	-0.24369	-1.03516	0.300596	0.997402
MINPP1	165.5892	-0.64885	-2.75479	0.005873	0.142751
MTRNR2L1	158.4455	0.004354	0.018483	0.985254	0.997402
NBEAL2	169.787	0.060098	0.255082	0.79866	0.997402
TRA2B	168.8792	0.125295	0.531761	0.594892	0.997402
OCIAD1	162.1074	0.17188	0.729325	0.465803	0.997402
SERPINB9	182.3902	-0.17411	-0.73869	0.460095	0.997402
RRM2	163.272	0.320748	1.360744	0.173594	0.997402
ELOB	162.381	-0.21941	-0.9304	0.352164	0.997402
NUMB	184.9812	0.157978	0.669533	0.503155	0.997402
RHOH	167.6855	0.435892	1.847262	0.064709	0.708764
LAMTOR4	168.1591	-0.20728	-0.8784	0.379726	0.997402
PRPF6	158.547	-0.08048	-0.34091	0.733169	0.997402
AD000864	167.4867	-0.02111	-0.08942	0.928752	0.997402
NDUFS5	161.3162	0.079259	0.335445	0.73729	0.997402
SELENOW	158.5813	-0.13605	-0.57571	0.564814	0.997402
ATF6B	166.5287	-0.2147	-0.9081	0.363828	0.997402
VTI1B	158.6731	-0.01773	-0.07492	0.940281	0.997402
UBE2I	181.6369	-0.30988	-1.30966	0.190311	0.997402
PNISR	157.6857	0.230599	0.974394	0.329861	0.997402
DERL1	167.2779	0.032396	0.136855	0.891145	0.997402
LAMP2	178.6039	0.332486	1.40342	0.160492	0.997402
HSD3B7	164.0762	-0.14706	-0.62051	0.53492	0.997402
UQCR10	162.6137	0.127004	0.535712	0.592158	0.997402
DDAH2	227.0408	-0.55847	-2.35294	0.018625	0.329041
ICAM3	192.2937	0.604579	2.545997	0.010897	0.225884
FYB1	168.3905	0.406769	1.71266	0.086775	0.809449
SLC35B2	171.5194	-0.4142	-1.74381	0.081192	0.783898
GNPTAB	170.2759	-1.21045	-5.09581	3.47E-07	3.15E-05
CPNE1	158.9907	0.077237	0.325068	0.74513	0.997402
OGT	160.2377	-0.12757	-0.5367	0.591476	0.997402
PTDSS1	199.7247	-0.13646	-0.57409	0.565909	0.997402
AP1G1	207.256	-0.4182	-1.75891	0.078593	0.775924
BUD31	162.9302	0.382064	1.606582	0.108146	0.907757

YPEL5	155.2123	-0.01997	-0.08395	0.933097	0.997402
HIF1AN	157.1068	-0.28733	-1.20766	0.227179	0.997402
CLTC	160.1052	-0.03418	-0.14364	0.885783	0.997402
COPS7A	165.8104	-0.12493	-0.52505	0.599551	0.997402
CSNK2A2	162.5338	-0.03228	-0.13565	0.892101	0.997402
DAPK1	192.7234	-0.19946	-0.83804	0.40201	0.997402
RBX1	154.8799	-0.09803	-0.41152	0.680691	0.997402
BUB3	158.6612	0.258129	1.083566	0.278557	0.997402
LARP4B	161.3039	0.071392	0.299608	0.764477	0.997402
JPT1	158.7645	0.080003	0.335737	0.737069	0.997402
ERLEC1	158.0093	-0.01484	-0.06226	0.950356	0.997402
CLNS1A	182.7593	-0.15562	-0.6525	0.514076	0.997402
USP15	164.2845	0.274947	1.152621	0.249066	0.997402
CMTM7	157.0971	-0.05399	-0.22633	0.820943	0.997402
ADRM1	160.8431	0.322995	1.353573	0.175873	0.997402
AZIN1	187.0328	-0.03172	-0.13287	0.8943	0.997402
SRSF4	174.7379	0.182108	0.762699	0.445643	0.997402
UBE2L3	156.251	-0.2908	-1.21759	0.223378	0.997402
MIR1282	155.0518	0.039461	0.165206	0.868782	0.997402
MIDEAS	156.9201	-0.30166	-1.26285	0.206644	0.997402
ELMO2	170.646	0.217423	0.909554	0.363058	0.997402
AL008726.	158.9232	0.161365	0.674987	0.499684	0.997402
COX7A2L	172.6845	0.084439	0.353175	0.723957	0.997402
KCNAB2	174.9689	-0.05459	-0.2283	0.81941	0.997402
FCGRT	158.1645	0.100199	0.418789	0.67537	0.997402
SLC35A4	156.2739	-0.12412	-0.51867	0.603988	0.997402
PMPCB	162.474	0.065609	0.274083	0.784021	0.997402
ASMTL	153.5041	0.068254	0.285105	0.775564	0.997402
SH3KBP1	157.1833	-0.60093	-2.50947	0.012091	0.244293
MTPN	154.9593	-0.49851	-2.08142	0.037396	0.515278
GPR65	179.1658	0.706978	2.951741	0.00316	0.087545
AC091564	155.9391	-0.22101	-0.92266	0.356183	0.997402
IDH3G	160.0422	0.065651	0.274067	0.784033	0.997402
MPV17	163.349	-0.12865	-0.53668	0.591487	0.997402
ZNF106	153.0109	0.278902	1.163428	0.244656	0.997402
UQCRFS1	152.8055	0.350355	1.46138	0.143911	0.997402
NDEL1	154.9176	-0.53709	-2.23936	0.025133	0.401978
UNC45A	156.7638	-0.13291	-0.55406	0.579535	0.997402
ZMYND8	154.1623	-0.13191	-0.54959	0.582601	0.997402
POR	160.3938	0.112584	0.469001	0.639069	0.997402
AC006030	154.0562	-0.21611	-0.90016	0.368038	0.997402
SUPT16H	173.8176	0.009772	0.040703	0.967532	0.997402
ADCY7	162.8373	-0.00141	-0.00589	0.995303	0.999604
ADAR	152.1895	-0.02035	-0.0847	0.932496	0.997402
DLC1	155.121	0.49255	2.050315	0.040334	0.53772
MTREX	159.2458	0.620604	2.583352	0.009785	0.209752
BCAT1	177.7314	-1.00982	-4.20335	2.63E-05	0.001474
MRPS27	151.5115	0.021171	0.088114	0.929786	0.997402
UFM1	169.2149	0.086094	0.358079	0.720284	0.997402
SRP72	152.1441	0.15305	0.636531	0.524431	0.997402

IVD	152.3665	0.186382	0.774981	0.438351	0.997402
KHNYN	153.6508	-0.01061	-0.04407	0.964845	0.997402
BCKDK	156.5802	0.37422	1.555037	0.119937	0.94989
RUVBL1	164.0116	0.056296	0.233932	0.815038	0.997402
SYTL2	157.2649	-1.04307	-4.33321	1.47E-05	0.000934
CYB5B	154.8862	-0.44869	-1.86376	0.062355	0.695693
EMC7	155.0481	0.275473	1.144066	0.252596	0.997402
SNRPB	165.553	0.19488	0.8093	0.418343	0.997402
AP3M1	153.793	0.035722	0.148278	0.882123	0.997402
NOP58	156.16	0.841041	3.49103	0.000481	0.018756
LONP2	153.4416	0.032069	0.133082	0.894129	0.997402
SEPTIN11	153.0818	-0.23639	-0.98094	0.326624	0.997402
HUWE1	174.373	-0.03556	-0.14752	0.882725	0.997402
YIF1B	166.898	-0.02223	-0.0922	0.926539	0.997402
TSPAN3	151.453	-0.31505	-1.30688	0.191255	0.997402
STK4	153.0334	0.002666	0.011057	0.991178	0.998821
AC018362	156.2804	0.110138	0.456769	0.647837	0.997402
CHD9	150.6645	-0.13593	-0.56318	0.573315	0.997402
CST7	160.2051	0.643395	2.66568	0.007683	0.174427
GTF2A2	153.7964	0.620118	2.569238	0.010192	0.214381
CBLB	152.2441	-0.56319	-2.3333	0.019632	0.341286
GUK1	165.1091	-0.53338	-2.20917	0.027163	0.422709
RASSF5	164.8893	-0.2341	-0.9693	0.332395	0.997402
ARMCX3	162.0297	-0.12128	-0.50211	0.615592	0.997402
NUTF2	154.3312	-0.03235	-0.13393	0.893455	0.997402
TRIM27	162.6377	-0.39607	-1.63933	0.101145	0.875634
NOP16	150.6497	0.456404	1.888772	0.058922	0.67932
ATP5PD	154.7793	0.373989	1.547011	0.121861	0.955088
DYNLT1	165.2905	-0.27401	-1.1333	0.25709	0.997402
MFSD10	166.2223	0.287425	1.188762	0.234533	0.997402
CSF3R	172.0718	-0.51868	-2.14468	0.031978	0.464775
JAK3	160.4157	-0.78309	-3.23763	0.001205	0.038766
EMC3	156.6247	0.680824	2.814534	0.004885	0.122934
ANAPC11	151.9877	-0.19929	-0.82384	0.410029	0.997402
LRRFIP1	153.9181	0.287138	1.186527	0.235414	0.997402
DCBLD2	153.9989	0.192342	0.794438	0.426941	0.997402
LEPROT	149.1024	0.078097	0.322556	0.747031	0.997402
TBCB	225.3802	-0.59438	-2.45428	0.014117	0.273876
PLPBP	151.1119	-0.06764	-0.27922	0.780079	0.997402
NAA15	152.471	0.165559	0.682988	0.494614	0.997402
GYG1	159.4812	-0.10475	-0.43208	0.665682	0.997402
EPRS1	156.2999	0.119325	0.492048	0.622686	0.997402
TMEM109	163.091	-0.24974	-1.02981	0.3031	0.997402
STXBP5	235.8816	-0.56008	-2.3073	0.021038	0.356306
OTUB1	155.5885	-0.02665	-0.10977	0.912594	0.997402
DDX24	149.9376	0.226999	0.934728	0.349929	0.997402
PTPRE	184.8013	1.906808	7.849582	4.17E-15	9.36E-13
ADD1	156.2011	0.062785	0.258438	0.796069	0.997402
PLA2G4A	157.8366	-0.06659	-0.27408	0.784021	0.997402
AP001267	150.2201	0.268727	1.105971	0.268739	0.997402

DCTN1	149.2515	0.045548	0.187447	0.85131	0.997402
S100A10	152.1122	-0.67148	-2.76334	0.005721	0.140054
EIF4A3	221.9018	0.607505	2.499669	0.012431	0.249355
CYB5R4	163.0393	0.156286	0.642982	0.520236	0.997402
IK	181.3744	0.159758	0.657186	0.511061	0.997402
FAR2	150.4171	0.129225	0.531517	0.59506	0.997402
CAMLG	158.2083	-0.2389	-0.98257	0.325821	0.997402
PYGL	178.787	0.1335	0.548814	0.583133	0.997402
GAA	148.3393	0.320203	1.315998	0.188175	0.997402
NUMA1	155.2897	0.242747	0.996968	0.31878	0.997402
NFE2L2	153.7301	-0.0403	-0.16546	0.868579	0.997402
POLR1D	157.4863	-0.03474	-0.14259	0.886611	0.997402
MTF2	147.6309	0.11698	0.479904	0.631295	0.997402
LYPLA1	155.2334	0.4433	1.818271	0.069023	0.729765
ANP32E	161.1558	0.372862	1.529264	0.126199	0.969144
FDPS	158.0296	0.019614	0.080438	0.935889	0.997402
RPA2	148.163	0.145389	0.595442	0.551548	0.997402
UBE2N	189.904	0.230138	0.942456	0.345959	0.997402
TPR	153.6646	-0.1813	-0.74232	0.457895	0.997402
COPE	165.3795	-0.01223	-0.05005	0.960086	0.997402
SLC39A1	151.4488	-0.10123	-0.41433	0.678633	0.997402
ANKRD27	153.2726	-0.11636	-0.47624	0.633904	0.997402
PSEN1	154.2349	-0.15237	-0.6234	0.533021	0.997402
NSMCE1	152.347	-0.46522	-1.90309	0.057029	0.665106
MYDGF	154.5994	0.416079	1.701696	0.088812	0.818865
GMPR	189.7885	-1.0293	-4.20916	2.56E-05	0.001442
ANP32B	151.4353	0.060544	0.247506	0.804516	0.997402
HCFC1	147.2563	0.035841	0.146475	0.883547	0.997402
VPS26C	148.5474	0.069845	0.285339	0.775384	0.997402
STAT6	161.3262	-0.18362	-0.75007	0.453214	0.997402
TAF10	157.3977	-0.36736	-1.50049	0.133489	0.983678
ABHD4	161.9386	-0.2394	-0.97755	0.328296	0.997402
TMX1	170.761	0.284507	1.161592	0.245401	0.997402
CFLAR	158.0208	-0.23762	-0.97018	0.331959	0.997402
TMEM87A	147.2174	-0.14088	-0.57504	0.565262	0.997402
TMEM164	158.5306	-0.85485	-3.48881	0.000485	0.018852
CASP4	154.2057	0.271475	1.107428	0.268109	0.997402
CD2BP2	147.9464	0.198854	0.811002	0.417364	0.997402
STOML2	151.959	0.436693	1.780905	0.074928	0.755887
ATP5PF	150.2678	0.190821	0.77808	0.436522	0.997402
ARHGAP1	163.4541	-0.09539	-0.38896	0.697304	0.997402
HERC1	185.569	-0.06341	-0.25849	0.796031	0.997402
STAM	157.9566	0.4701	1.916293	0.055328	0.653233
TIMP3	150.8991	-0.86924	-3.54227	0.000397	0.015896
VAV1	146.2906	0.016072	0.065471	0.947799	0.997402
TRIM13	151.6927	0.006801	0.027703	0.977899	0.997402
SH3D19	149.7996	0.506691	2.063631	0.039053	0.527506
ATP5MD	145.6323	0.148424	0.604327	0.545626	0.997402
NDUFS8	145.1398	0.059179	0.240944	0.809599	0.997402
POLR2G	144.6334	0.109134	0.444288	0.656834	0.997402

SNX4	168.3321	-0.22745	-0.92594	0.35448	0.997402
RNF13	155.5643	0.588336	2.394874	0.016626	0.30421
ZDHHC12	145.8244	0.054194	0.220531	0.825457	0.997402
SGPL1	168.5871	0.990712	4.031415	5.54E-05	0.00284
IARS1	219.1659	0.135962	0.553229	0.580106	0.997402
ETF1	154.2356	0.185796	0.755787	0.449777	0.997402
VSIG4	150.032	-0.65832	-2.67768	0.007413	0.170908
NOTCH2	148.16	-0.50607	-2.05766	0.039623	0.532701
DDX21	144.9994	0.252111	1.024982	0.305371	0.997402
GGCX	169.8268	0.051937	0.2111	0.832809	0.997402
DCTN4	154.7769	0.291281	1.18391	0.236449	0.997402
DHX9	225.1656	0.081018	0.329267	0.741954	0.997402
NDUFA6	157.1609	0.202482	0.822884	0.410574	0.997402
GTPBP4	146.2517	0.304983	1.239375	0.215207	0.997402
NECAP2	156.7393	-0.09381	-0.38119	0.703059	0.997402
SNRPG	147.8506	0.369557	1.500838	0.133398	0.983678
SDHA	151.4939	0.126981	0.515666	0.606088	0.997402
SUSD6	158.3709	-0.01161	-0.04714	0.9624	0.997402
DPF2	146.3728	0.32307	1.311095	0.189826	0.997402
TES	150.8074	-0.10005	-0.40602	0.684724	0.997402
CXCL16	147.8737	-0.82312	-3.33971	0.000839	0.029487
WBP1L	146.818	-0.61736	-2.50475	0.012254	0.246537
SPAG7	148.4616	0.020971	0.085079	0.932198	0.997402
SLC40A1	176.5954	-0.29219	-1.18524	0.235921	0.997402
HAX1	143.5165	-0.09024	-0.36603	0.714342	0.997402
SSB	156.2183	0.089993	0.364958	0.715143	0.997402
GLRX	143.4772	-0.08982	-0.36424	0.715677	0.997402
LPP	144.4175	-0.42297	-1.71456	0.086426	0.808693
ECHS1	153.0395	0.18307	0.742034	0.458066	0.997402
DALRD3	147.6372	-0.24782	-1.00434	0.315215	0.997402
TMEM50B	144.0933	-0.03061	-0.124	0.901318	0.997402
RMND5B	151.0312	-0.05656	-0.22907	0.818814	0.997402
HMGB2	147.7047	-0.0133	-0.05387	0.95704	0.997402
BIRC6	155.6347	0.648075	2.624313	0.008682	0.192502
ATIC	146.2803	0.254299	1.02957	0.303212	0.997402
DNM2	146.2366	0.186961	0.756907	0.449106	0.997402
USP14	145.7457	0.0156	0.063153	0.949644	0.997402
TLK1	146.058	0.088941	0.360012	0.718838	0.997402
FAM120B	147.7567	-0.11034	-0.44644	0.655276	0.997402
TCN1	186.8273	-0.12679	-0.51294	0.607991	0.997402
LAX1	153.7635	0.561123	2.269475	0.023239	0.37926
FIBP	156.0241	-0.06475	-0.26189	0.79341	0.997402
WDR41	146.1308	-0.39132	-1.58257	0.113519	0.926743
PRPF8	154.9461	-0.00425	-0.01717	0.986299	0.997402
JKAMP	144.1243	0.383504	1.54934	0.1213	0.953451
SYNCRIP	146.6325	0.126825	0.511703	0.608859	0.997402
ENO2	175.6886	-0.57846	-2.33356	0.019619	0.341286
NIPSNAP3/	143.5621	-0.61666	-2.48727	0.012873	0.25576
ACTN4	143.8577	-0.26986	-1.08845	0.276395	0.997402
CNOT2	146.535	-0.03763	-0.15155	0.87954	0.997402

CCDC115	141.5017	-0.09194	-0.37027	0.71118	0.997402
GORASP2	160.4683	-0.10696	-0.43075	0.666651	0.997402
CCR1	149.98	0.661956	2.665179	0.007695	0.174427
TUBB6	145.5068	-0.44152	-1.77705	0.075559	0.757442
OGDH	142.7089	-0.0649	-0.26119	0.793948	0.997402
EIF5A	172.038	-0.10864	-0.43721	0.661957	0.997402
CD300C	151.4621	0.14888	0.599071	0.549126	0.997402
TIAM1	143.4425	0.715752	2.879458	0.003984	0.104469
SPN	209.7162	0.362787	1.458696	0.144649	0.997402
AL592166.	153.5636	0.228923	0.920437	0.357344	0.997402
RO60	171.3973	-0.04308	-0.17317	0.862515	0.997402
MEIS2	146.6464	-0.84683	-3.40269	0.000667	0.024798
ATP8B4	186.8104	-0.32449	-1.3038	0.1923	0.997402
VDAC2	143.1414	0.437899	1.759286	0.078529	0.775924
RAP1GDS1	142.4391	-0.52357	-2.10272	0.03549	0.49458
EIF2S1	146.4501	0.524765	2.107522	0.035072	0.490621
DPYD	207.2319	-0.10454	-0.41983	0.674607	0.997402
TAF9	140.7752	0.243	0.97513	0.329496	0.997402
EMC4	151.1385	-0.00228	-0.00915	0.992702	0.999461
EIF2B1	150.6912	-0.0293	-0.11747	0.906484	0.997402
SMYD3	151.1786	-1.20118	-4.81535	1.47E-06	0.00012
SQLE	168.3991	-0.48412	-1.94035	0.052337	0.631439
PLTP	141.5835	-0.37227	-1.49164	0.135793	0.990539
PYCARD	145.9544	-0.55275	-2.21441	0.026801	0.421098
RIOK3	139.72	0.141533	0.566992	0.57072	0.997402
ZNF460	144.2036	0.10758	0.430919	0.666527	0.997402
SAE1	145.6179	-0.0495	-0.19811	0.842961	0.997402
GLOD4	139.5538	-0.09394	-0.37598	0.706935	0.997402
LTA4H	143.1797	-0.81533	-3.26298	0.001102	0.036356
MACROH2	142.2279	0.18986	0.759624	0.447479	0.997402
GPAT4	142.5283	-0.29599	-1.184	0.236413	0.997402
RAB10	206.7952	-0.20354	-0.81407	0.415605	0.997402
SNHG32	150.328	0.47387	1.895115	0.058077	0.674042
CIAO2B	139.8748	0.075554	0.30212	0.76256	0.997402
TDRD3	171.4123	-1.9746	-7.89393	2.93E-15	6.78E-13
TOR1A	141.3292	0.045647	0.182445	0.855233	0.997402
SRP54	140.6378	-0.0023	-0.00919	0.992669	0.999461
EEF2K	149.0217	0.741013	2.958106	0.003095	0.086555
PLAU	144.4154	0.952889	3.8034	0.000143	0.006418
CCDC18-A!	141.2208	-0.15884	-0.63395	0.52611	0.997402
ACADVL	157.0792	-0.33297	-1.32797	0.184188	0.997402
SLC44A1	153.2976	-0.48015	-1.91438	0.055572	0.653964
ELAC2	143.4308	0.074824	0.298184	0.765562	0.997402
PRMT1	145.5153	0.230247	0.917559	0.35885	0.997402
SLC7A8	191.7493	2.800655	11.15909	6.47E-29	4.03E-26
NXF1	144.2878	0.3687	1.468456	0.14198	0.997402
SLCO2B1	171.2699	0.048026	0.191266	0.848317	0.997402
ACIN1	144.3145	0.056102	0.223401	0.823223	0.997402
TADA3	181.6311	0.216514	0.862022	0.388675	0.997402
MRPL3	146.7983	0.566089	2.253587	0.024222	0.393062

FER	146.2884	-0.45382	-1.80638	0.070859	0.743036
DCXR	141.2936	0.272291	1.083805	0.278451	0.997402
HNRNPA3	139.0193	-0.04217	-0.16782	0.866726	0.997402
TTN	164.966	0.307811	1.224816	0.220645	0.997402
PSMC6	143.2561	0.190226	0.756735	0.449209	0.997402
DAXX	143.8044	-0.07634	-0.30367	0.761383	0.997402
LAP3	139.5224	0.547908	2.178989	0.029332	0.441063
HSD17B12	150.8497	0.189651	0.754011	0.450843	0.997402
RNF114	139.225	0.079253	0.315083	0.752699	0.997402
SDR39U1	146.4791	0.043051	0.171129	0.864123	0.997402
HACD3	139.2529	0.08942	0.355224	0.722422	0.997402
TIAM2	139.7795	0.202376	0.803939	0.421432	0.997402
GOLGA4	145.1826	-0.31291	-1.24302	0.213862	0.997402
IP6K2	137.3333	0.215932	0.857678	0.39107	0.997402
CERS5	151.0519	0.749959	2.976408	0.002916	0.082518
CNOT8	139.5052	0.126599	0.502248	0.615493	0.997402
BCL2L1	138.8388	0.533223	2.114775	0.034449	0.486648
FBXO34	146.2955	-0.26468	-1.04971	0.29385	0.997402
NFKBIA	242.7681	-0.24931	-0.9884	0.322958	0.997402
DYNLRB1	140.8616	-0.0056	-0.02218	0.982301	0.997402
TANK	137.2632	0.111263	0.441063	0.659168	0.997402
AC012640	137.5302	0.468606	1.856665	0.063359	0.701222
SNRPD1	137.3753	0.342795	1.358166	0.174411	0.997402
DOK1	139.6842	-0.16042	-0.63549	0.525112	0.997402
SRRM1	136.5369	0.212343	0.840971	0.400364	0.997402
CDK6	208.7639	1.947335	7.711282	1.25E-14	2.67E-12
AC008894	138.1527	-0.55641	-2.2014	0.027708	0.426251
C1orf162	136.7258	0.069667	0.275609	0.782848	0.997402
KANSL3	139.8262	0.348759	1.379537	0.167729	0.997402
GABARAPL	136.2864	-0.13372	-0.52893	0.596856	0.997402
BUD23	135.9544	0.107724	0.425936	0.670154	0.997402
CTSW	154.9162	1.247004	4.930279	8.21E-07	7.01E-05
ZNFX1	143.5038	0.114237	0.451607	0.651552	0.997402
MPDU1	144.5861	0.453906	1.793868	0.072834	0.749504
MCM7	148.9606	0.000784	0.003099	0.997527	0.999604
SLC25A11	149.0956	-0.31374	-1.23983	0.215037	0.997402
COG5	150.6952	-0.29049	-1.14731	0.251255	0.997402
SRPRB	141.5773	-0.16559	-0.65395	0.513144	0.997402
DYNC1H1	146.8102	0.026694	0.105416	0.916046	0.997402
AATF	135.8639	0.239312	0.945011	0.344653	0.997402
PARP9	143.9315	-0.63463	-2.50541	0.012231	0.246537
PHC1	141.3342	0.461997	1.822916	0.068316	0.728079
NEMF	147.3582	0.003053	0.012036	0.990397	0.998541
RARS1	138.461	0.117617	0.463624	0.642917	0.997402
RBM23	149.1967	-0.0104	-0.041	0.967295	0.997402
NPEPPS	138.2841	0.579627	2.283633	0.022393	0.369654
ACOT9	136.3572	-0.1245	-0.49047	0.623805	0.997402
MCM3	140.918	-0.21341	-0.84056	0.400595	0.997402
RSRP1	150.9161	0.266833	1.04988	0.293773	0.997402
ATP6V1C1	134.705	0.006495	0.02555	0.979616	0.997402

AIP	138.0926	-0.09521	-0.37451	0.708028	0.997402
HARS1	139.455	0.284675	1.1196	0.262884	0.997402
THOC2	134.5838	-0.05587	-0.21947	0.826283	0.997402
VPS35L	134.9834	-0.19349	-0.75986	0.447341	0.997402
TMEM179	159.1708	-0.05737	-0.22529	0.82175	0.997402
VCL	136.8414	-0.59977	-2.35495	0.018525	0.328075
YME1L1	135.5935	0.202844	0.796424	0.425786	0.997402
CUTA	155.5267	-0.22257	-0.8733	0.382502	0.997402
MRFAP1L1	137.1959	0.175298	0.687735	0.491619	0.997402
KXD1	138.049	0.230296	0.902978	0.366538	0.997402
SMARCA5	137.9491	-0.01837	-0.07198	0.942616	0.997402
CHTOP	140.3035	-0.23536	-0.92244	0.3563	0.997402
ALDH9A1	138.7052	-0.20421	-0.80033	0.42352	0.997402
TXNDC17	141.5417	0.059933	0.234658	0.814474	0.997402
PPP6C	139.8877	-0.02669	-0.1045	0.916772	0.997402
ARID4B	137.0716	0.338675	1.325586	0.184977	0.997402
TOM1	137.7616	-0.30239	-1.18238	0.237055	0.997402
LPIN1	135.8184	0.165732	0.647976	0.517	0.997402
ZNF706	135.3105	-0.3201	-1.25125	0.210845	0.997402
PNRC2	134.738	0.10337	0.403848	0.686325	0.997402
KPNB1	138.4564	0.285975	1.117206	0.263906	0.997402
AC010768	137.4807	0.094993	0.371104	0.71056	0.997402
PSAT1	146.115	-0.41117	-1.6062	0.10823	0.90793
AC025165	133.3093	-0.07292	-0.28482	0.775779	0.997402
TECR	134.8011	0.230054	0.89864	0.368845	0.997402
ELANE	139.2205	-1.17101	-4.57264	4.82E-06	0.000339
MS4A3	133.1853	-0.05852	-0.22845	0.819294	0.997402
PRKAB1	152.2622	-0.32997	-1.28788	0.197788	0.997402
NADSYN1	138.5057	0.159131	0.620965	0.534623	0.997402
CSNK2A1	132.4119	-0.31847	-1.24224	0.214148	0.997402
NDUFA10	140.4901	0.023344	0.091048	0.927454	0.997402
SMAD2	135.1557	0.276444	1.078058	0.281008	0.997402
ACER3	146.0575	-0.49037	-1.91211	0.055862	0.655111
ACAA1	169.4497	-0.75048	-2.92586	0.003435	0.093325
ATF6	150.3187	0.042601	0.165935	0.868208	0.997402
PRPSAP2	136.4672	-0.6482	-2.52443	0.011589	0.236137
SLC43A3	134.6861	-0.37308	-1.45216	0.146457	0.997402
SUMO1	131.511	0.109232	0.425159	0.67072	0.997402
SAMHD1	141.7157	0.677321	2.636181	0.008384	0.187345
BRD8	139.4488	-0.17459	-0.6795	0.496819	0.997402
UBL4A	137.2591	0.102139	0.397512	0.69099	0.997402
TMEM258	133.1763	0.050622	0.196974	0.843848	0.997402
BTBD10	148.0154	-0.22278	-0.86658	0.386174	0.997402
COG1	131.826	0.113868	0.442867	0.657862	0.997402
NAA20	135.9312	0.227817	0.885877	0.375684	0.997402
PORCN	145.7324	-1.59339	-6.19009	6.01E-10	8.21E-08
GNPDA1	131.6659	-0.09248	-0.35926	0.719402	0.997402
SLC44A2	133.4059	-0.66377	-2.57796	0.009939	0.211212
TNRC6B	132.4614	0.012124	0.047084	0.962446	0.997402
MESD	135.2344	0.340072	1.320224	0.18676	0.997402

ACAT2	144.2692	-0.19783	-0.76799	0.44249	0.997402
ACSL5	135.6057	0.126802	0.491917	0.622778	0.997402
LUC7L3	133.8448	-0.09554	-0.37034	0.711131	0.997402
HEATR1	141.3637	-0.67701	-2.6235	0.008703	0.192662
ADAM12	134.2292	0.277705	1.075225	0.282274	0.997402
PLA2G15	133.3739	-0.26011	-1.00698	0.313945	0.997402
CPSF1	131.1048	0.18641	0.7211	0.470848	0.997402
GLRX3	140.5714	0.385976	1.492962	0.135447	0.990539
ST7	129.9774	0.06613	0.25573	0.79816	0.997402
NELFE	129.9739	0.04465	0.172648	0.862928	0.997402
SF3B6	130.3995	0.235571	0.910278	0.362676	0.997402
LAMTOR2	131.382	-0.02113	-0.08165	0.934927	0.997402
RRM1	130.7794	-0.29652	-1.1455	0.252003	0.997402
MAP3K7	140.1183	-0.09325	-0.3601	0.71877	0.997402
NUP93	135.6426	0.189956	0.733125	0.463482	0.997402
TOMM22	132.032	0.18494	0.71365	0.475444	0.997402
PSMD12	141.8372	0.166249	0.641476	0.521214	0.997402
USP39	129.4343	-0.00013	-0.00049	0.999609	0.999818
ARPC1A	132.6307	0.129063	0.497813	0.618616	0.997402
RAB11A	137.2379	-0.30291	-1.16835	0.242666	0.997402
CTNS	133.73	1.074169	4.143114	3.43E-05	0.001834
FIS1	137.5639	-0.22346	-0.86136	0.389038	0.997402
ATP6V1H	129.3518	0.478831	1.845486	0.064967	0.709246
ANXA11	163.2751	-0.79662	-3.06964	0.002143	0.062487
MAEA	131.3141	-0.05265	-0.20286	0.839241	0.997402
NCBP1	145.8251	0.222209	0.85614	0.391921	0.997402
FKBP15	150.9952	0.023968	0.092319	0.926444	0.997402
NAV1	133.5297	-0.61972	-2.38677	0.016997	0.309419
C1orf56	129.9802	-0.20251	-0.77973	0.435548	0.997402
IL4R	142.5022	-1.6052	-6.18014	6.40E-10	8.67E-08
GTF2B	135.4961	-0.02253	-0.08673	0.930888	0.997402
IKZF1	133.3251	0.600358	2.310425	0.020865	0.354625
FOPNL	131.7053	0.137336	0.528091	0.597436	0.997402
UXT	129.1695	-0.13946	-0.53604	0.591929	0.997402
RNASEH2C	133.5261	-0.02369	-0.09094	0.92754	0.997402
MRPL16	127.8806	-0.06703	-0.25716	0.797052	0.997402
RAB5A	127.9418	0.251182	0.963638	0.335227	0.997402
STRIP2	144.576	-1.70071	-6.52175	6.95E-11	1.08E-08
PGAM1	129.995	-0.24824	-0.95187	0.341165	0.997402
FTSJ3	134.184	0.179442	0.687789	0.491586	0.997402
CCND1	158.7747	-2.42427	-9.29036	1.54E-20	5.80E-18
CPM	139.564	1.055135	4.042914	5.28E-05	0.002714
MRPS21	130.0335	-0.1029	-0.39423	0.693413	0.997402
LYPLA2	131.8062	-0.02991	-0.1146	0.908762	0.997402
NDUFB2	131.2639	0.004225	0.016177	0.987094	0.997464
STXBP3	132.8693	-0.14942	-0.57206	0.567284	0.997402
SUPT5H	137.5762	0.389797	1.492051	0.135686	0.990539
STK17B	129.2623	-0.15362	-0.58786	0.556623	0.997402
COLGALT1	142.8366	0.147677	0.565022	0.572059	0.997402
ITPR1	135.5265	-0.33309	-1.27365	0.202789	0.997402

NDUFB4	157.1213	0.22036	0.841998	0.399789	0.997402
PRR13	129.3951	0.259311	0.990788	0.321789	0.997402
SNRPE	131.9592	0.108951	0.416168	0.677287	0.997402
PWP1	144.4032	0.298847	1.141401	0.253703	0.997402
IFI6	126.4815	-0.02356	-0.08989	0.928375	0.997402
DENND2D	134.4421	-0.6822	-2.6025	0.009255	0.202522
NFX1	132.2561	-0.05972	-0.2278	0.819805	0.997402
RASSF1	131.7695	-0.54133	-2.06484	0.038938	0.526458
TTC17	129.8775	0.249394	0.951242	0.341481	0.997402
PUM1	125.6233	0.134628	0.513454	0.607634	0.997402
METTL3	129.9203	0.056777	0.216532	0.828573	0.997402
CYP27A1	127.5324	-0.2998	-1.14335	0.252895	0.997402
EHD1	134.7518	-0.11264	-0.42954	0.667529	0.997402
DOCK8	133.584	0.709297	2.704651	0.006838	0.16106
CACUL1	141.6269	-0.15357	-0.58533	0.558329	0.997402
PAICS	135.0166	0.599246	2.283773	0.022385	0.369654
PPP2R5C	136.8125	-0.20883	-0.79573	0.42619	0.997402
BRD7	129.3742	0.092953	0.354019	0.723325	0.997402
TWF2	139.3068	0.02589	0.098603	0.921454	0.997402
MFN2	128.2017	0.357639	1.36191	0.173226	0.997402
MEA1	125.5649	-0.21233	-0.80833	0.418901	0.997402
NPRL3	130.3301	-0.40227	-1.53137	0.125679	0.968243
GALC	137.137	0.535591	2.038901	0.04146	0.546137
AAMP	126.4871	0.056225	0.214029	0.830524	0.997402
CD300LF	131.1112	0.851342	3.240014	0.001195	0.03853
MAGT1	126.8132	0.11804	0.449143	0.653329	0.997402
CFP	156.1209	0.874492	3.327399	0.000877	0.030596
PAK2	125.2038	0.28466	1.083063	0.27878	0.997402
SELENOK	125.9528	0.323261	1.229664	0.218823	0.997402
CARD16	132.7782	-0.05201	-0.19783	0.843179	0.997402
PDHA1	133.8578	0.405806	1.543596	0.122686	0.957903
RGS2	143.407	-0.80847	-3.07504	0.002105	0.061617
CHFR	133.2898	-0.02797	-0.1064	0.915266	0.997402
CAST	129.8087	-0.64461	-2.45042	0.014269	0.274383
MCM5	125.9648	-0.38418	-1.46032	0.144202	0.997402
SH2B3	127.3483	-0.23719	-0.90153	0.367304	0.997402
AP3B1	128.0321	-0.03495	-0.13282	0.894332	0.997402
RIPOR1	130.9323	-0.0337	-0.12796	0.898178	0.997402
MDM2	127.2856	0.406614	1.542369	0.122984	0.958808
CLDND1	152.884	0.636014	2.412323	0.015851	0.293818
CYFIP1	156.0658	-0.37078	-1.40597	0.159732	0.997402
NASP	130.0765	0.108305	0.410644	0.681333	0.997402
ARSA	126.6544	-0.10919	-0.41398	0.678886	0.997402
ITGA4	125.5337	-0.36283	-1.37547	0.168985	0.997402
PSMC4	136.6671	0.16704	0.633117	0.526657	0.997402
H4C3	125.2635	0.364664	1.381698	0.167065	0.997402
SEMA4D	128.103	0.418963	1.586768	0.112565	0.92324
GFM1	145.4974	0.48817	1.848723	0.064498	0.707897
CPSF6	161.0632	0.08498	0.321647	0.74772	0.997402
SRSF11	149.0937	-0.26564	-1.00533	0.31474	0.997402

TOR1AIP2	123.3635	0.130819	0.495061	0.620557	0.997402
FAH	134.1667	-0.16465	-0.62269	0.533486	0.997402
RBM17	125.0123	0.021754	0.082258	0.934441	0.997402
GMPR2	126.6225	-0.45519	-1.72103	0.085245	0.803328
ATG7	124.1399	0.12431	0.46998	0.638369	0.997402
ACP2	123.2763	-0.00834	-0.03153	0.974848	0.997402
GLIPR2	125.3188	0.47056	1.778974	0.075244	0.756925
RASSF2	150.9529	-0.45178	-1.70793	0.087649	0.813323
SNW1	130.5352	0.009027	0.034123	0.972779	0.997402
PNP	139.3787	-0.06999	-0.26459	0.791324	0.997402
TMA7	126.3269	0.220739	0.834422	0.404044	0.997402
ALDH18A1	126.5768	0.18098	0.683975	0.493991	0.997402
FCGR2A	133.831	1.563198	5.904456	3.54E-09	4.38E-07
SLC5A3	155.3024	-0.00918	-0.03466	0.972351	0.997402
SEC62	126.0914	-0.12016	-0.4537	0.650048	0.997402
ANKRD44	127.7023	-0.02765	-0.10438	0.916866	0.997402
RIT1	126.7958	0.396485	1.496509	0.134521	0.987683
MBTPS1	126.6244	0.226231	0.853662	0.393292	0.997402
MTMR14	125.395	0.599811	2.262384	0.023674	0.385033
ERI1	126.8755	0.616756	2.32582	0.020028	0.344612
KIAA1522	131.5282	0.837227	3.156517	0.001597	0.049256
RFX5	149.3175	-0.15726	-0.59253	0.553499	0.997402
COQ10B	131.6734	0.107581	0.405179	0.685346	0.997402
PSMA3-AS	123.5152	0.009806	0.036929	0.970541	0.997402
ZMIZ1	130.4493	-0.18911	-0.71184	0.476562	0.997402
FAM102A	124.6297	0.573249	2.157381	0.030976	0.455744
PBX1	125.8547	-0.32241	-1.21334	0.225001	0.997402
NOP2	126.2539	0.731959	2.754332	0.005881	0.142751
PTK2B	151.2617	-0.19319	-0.72687	0.467307	0.997402
GAPVD1	125.5697	0.204894	0.770886	0.440775	0.997402
ZC3H15	138.2576	0.351512	1.322357	0.186049	0.997402
CD44-AS1	129.0766	-0.35241	-1.32567	0.184949	0.997402
LAMTOR3	123.6476	-0.1228	-0.46194	0.644127	0.997402
NPL	126.3079	-0.02023	-0.07607	0.939366	0.997402
HBP1	125.8155	-0.69134	-2.59902	0.009349	0.202957
NUP98	131.7795	0.072265	0.271627	0.785909	0.997402
RRN3	144.878	0.659649	2.479457	0.013158	0.259993
ST3GAL2	122.8207	-0.21154	-0.79493	0.426652	0.997402
DNAJA2	134.2346	0.180471	0.678149	0.497677	0.997402
DCAF12	125.3415	0.014772	0.055504	0.955737	0.997402
MFSD14A	121.6527	-0.18541	-0.69655	0.486084	0.997402
TET2	131.1582	-0.17994	-0.67599	0.499048	0.997402
SNRPD3	126.9698	-0.04754	-0.17855	0.858289	0.997402
CLK1	127.1653	-0.34916	-1.3112	0.189792	0.997402
BID	122.3868	0.516098	1.937044	0.05274	0.633133
DYM	124.2448	0.084958	0.318862	0.749831	0.997402
DHX30	123.6085	0.218435	0.819467	0.41252	0.997402
RAP1B	125.6169	0.19336	0.725356	0.468234	0.997402
FAM50A	125.3949	-0.13753	-0.5159	0.605923	0.997402
TRIM22	129.2322	0.658835	2.469924	0.013514	0.265479

PMPCA	122.3751	0.480487	1.801133	0.071682	0.745669
ATP1A1	134.6415	0.134838	0.505433	0.613255	0.997402
CDCP1	134.4302	-1.1719	-4.39153	1.13E-05	0.000744
TIMM23	122.2298	0.230251	0.862774	0.388262	0.997402
GSDMD	123.8179	-0.58133	-2.17694	0.029485	0.441456
METAP1	120.752	-0.05666	-0.21217	0.831975	0.997402
MED4	130.6478	-0.24933	-0.93304	0.350799	0.997402
SEMA7A	125.2777	-0.14841	-0.55506	0.578854	0.997402
CUX1	123.6398	0.264579	0.989287	0.322523	0.997402
DNAJB9	122.1739	-0.41426	-1.54892	0.121401	0.953724
PRDX4	127.9866	0.14684	0.548694	0.583215	0.997402
AL354733.	120.1499	0.150757	0.563287	0.57324	0.997402
TYK2	124.7574	0.342059	1.277941	0.20127	0.997402
NINJ1	136.5356	0.469904	1.755109	0.079241	0.778427
PRPF38A	123.3836	0.036292	0.135514	0.892205	0.997402
PRCP	127.2186	0.45355	1.693495	0.090361	0.827735
KPNA1	123.4335	-0.15269	-0.57006	0.568636	0.997402
RASGRP4	129.9161	0.392664	1.464905	0.142947	0.997402
UBXN4	155.4172	0.166731	0.621957	0.53397	0.997402
CLK3	126.6114	-0.01473	-0.05494	0.956184	0.997402
CD83	154.7824	1.279191	4.769838	1.84E-06	0.000145
BIRC3	147.1117	2.218441	8.26874	1.35E-16	3.73E-14
ARG2	129.1647	-0.19802	-0.73801	0.460506	0.997402
WDR33	124.3276	0.403469	1.503495	0.132711	0.983401
HMGN4	119.8605	-0.25509	-0.95028	0.341972	0.997402
MTSS1	130.1738	-0.72159	-2.68788	0.007191	0.167045
BTN3A2	126.2976	-1.05423	-3.92621	8.63E-05	0.004248
RBM25	128.5513	0.428861	1.596825	0.110305	0.918885
CELF1	128.8413	-0.32622	-1.21459	0.224523	0.997402
TMEM219	121.3996	-0.48333	-1.79901	0.072017	0.746201
SLTM	121.2724	0.21983	0.818091	0.413305	0.997402
CCL4	136.9089	1.149893	4.279268	1.88E-05	0.001121
AC120057.	130.3942	0.057555	0.214186	0.830402	0.997402
VPS41	133.128	-0.25399	-0.94509	0.344615	0.997402
SCFD1	121.7006	-0.23391	-0.87017	0.384207	0.997402
SNX14	119.4238	0.108713	0.404382	0.685932	0.997402
METTL17	129.7595	-0.11835	-0.44021	0.659784	0.997402
ATF7IP	123.346	0.026464	0.098433	0.921588	0.997402
RAB20	132.0784	-0.68787	-2.55774	0.010535	0.220305
DCTN3	131.4055	-0.2334	-0.8678	0.385507	0.997402
DNAJB6	123.999	0.080288	0.298465	0.765349	0.997402
PRKCB	121.5267	-0.76152	-2.83025	0.004651	0.117881
ECH1	133.9924	0.010253	0.038105	0.969604	0.997402
SPCS3	122.235	0.385831	1.43384	0.151618	0.997402
LARS1	128.5119	0.341343	1.267928	0.204824	0.997402
SF3B2	123.5556	0.457627	1.699787	0.089171	0.82146
IRF2	129.5845	-0.03862	-0.14344	0.885944	0.997402
RUSC1-AS1	118.9289	-0.16071	-0.59677	0.550662	0.997402
DUSP4	132.0684	0.848452	3.149688	0.001634	0.050314
C9orf78	147.7963	0.32016	1.188462	0.234652	0.997402

ARL2BP	124.4005	-0.17609	-0.65365	0.513335	0.997402
ADD3	140.6937	0.46766	1.735224	0.082701	0.789845
STK38	146.703	-0.26779	-0.99356	0.320437	0.997402
NDUFS7	124.6858	0.028838	0.106992	0.914796	0.997402
POLE3	136.0774	0.027891	0.103466	0.917593	0.997402
TTC19	127.8531	0.002039	0.007564	0.993965	0.99958
KAT7	124.23	-0.41855	-1.55229	0.120593	0.952589
C6orf89	121.7001	-0.01919	-0.07117	0.94326	0.997402
SNRNP40	122.9545	0.177897	0.65969	0.509453	0.997402
ACTL6A	120.5432	0.251185	0.931301	0.351698	0.997402
TRA2A	122.0063	0.213741	0.791461	0.428675	0.997402
PYURF	123.8136	-0.13865	-0.5133	0.607743	0.997402
DARS1	119.4467	0.204794	0.758061	0.448415	0.997402
NFYC	121.6882	0.161744	0.598702	0.549372	0.997402
ERCC1	127.5695	0.154855	0.573198	0.56651	0.997402
KCTD10	126.1387	0.364238	1.348138	0.177614	0.997402
C16orf70	119.5988	0.006539	0.024197	0.980695	0.997402
RABGGTB	123.821	0.345922	1.280001	0.200545	0.997402
SLC7A5	134.9646	0.821848	3.040791	0.00236	0.068105
ATP5MPL	118.9123	-0.18516	-0.68479	0.493479	0.997402
TM6SF1	117.7521	-0.09568	-0.35384	0.723461	0.997402
C5orf15	125.8904	-0.29874	-1.10469	0.269293	0.997402
HAT1	118.3317	-0.04747	-0.17548	0.860706	0.997402
TBC1D2	135.6542	-0.9593	-3.54609	0.000391	0.015789
SEM1	117.7269	-0.09553	-0.35308	0.724031	0.997402
TPCN1	120.8002	-0.67097	-2.4799	0.013142	0.259993
UBQLN2	121.3188	-0.23954	-0.8844	0.376479	0.997402
SNRPC	129.3418	0.35226	1.300204	0.193531	0.997402
ADH5	117.1671	-0.25615	-0.94528	0.344514	0.997402
SDHC	117.3776	0.098886	0.364878	0.715203	0.997402
SUGT1	134.9279	0.230916	0.851931	0.394253	0.997402
USB1	118.9145	0.230997	0.851934	0.394251	0.997402
DOCK5	117.8703	-0.29652	-1.09313	0.274337	0.997402
LIMD2	137.9935	0.6173	2.27564	0.022868	0.374351
SYK	127.6582	-0.32369	-1.1931	0.232831	0.997402
ITGB1	138.3062	-0.05608	-0.20671	0.836237	0.997402
MOB3C	128.1728	1.411133	5.197962	2.01E-07	1.91E-05
TRIM38	119.7198	-0.00931	-0.03428	0.972652	0.997402
SPECC1	121.0545	-0.7151	-2.63269	0.008471	0.188693
RPS15A	117.8362	0.062718	0.230895	0.817396	0.997402
PLRG1	118.8107	-0.10837	-0.39895	0.689929	0.997402
EXT2	119.4694	-0.02766	-0.10179	0.918925	0.997402
BATF	125.8269	0.349782	1.287199	0.198025	0.997402
TNPO1	120.0024	0.277595	1.021439	0.307046	0.997402
NDUFS1	125.9178	-0.09063	-0.33348	0.738775	0.997402
PARP4	118.9671	0.15796	0.581207	0.561101	0.997402
METAP2	125.664	0.317464	1.167742	0.242911	0.997402
GBF1	118.2073	0.189538	0.697102	0.485739	0.997402
VBP1	116.4772	0.147253	0.541525	0.588146	0.997402
IMP3	119.6354	0.005955	0.021892	0.982534	0.997402

CDK2AP2	121.0283	-0.2087	-0.76716	0.442988	0.997402
RAD23A	124.0992	0.216624	0.796241	0.425892	0.997402
SUCLG1	124.3041	-0.1372	-0.50404	0.614232	0.997402
EXOC6B	117.1925	-0.52225	-1.91828	0.055076	0.651349
PCIF1	120.8856	-0.24186	-0.88832	0.374367	0.997402
USP22	122.8401	-0.41401	-1.52055	0.128372	0.972853
PCNX4	123.7286	-0.135	-0.49581	0.620027	0.997402
CRYZ	126.9531	-0.19577	-0.71881	0.472256	0.997402
DNAJB12	126.6126	0.086606	0.317978	0.750502	0.997402
PITRM1	131.053	0.100193	0.367811	0.713014	0.997402
NHP2	117.9743	-0.05192	-0.19056	0.848874	0.997402
GTF3C6	125.8899	0.327483	1.20147	0.229569	0.997402
PPM1H	140.8737	-1.23055	-4.51373	6.37E-06	0.000439
FAM104A	118.3519	-0.07284	-0.26714	0.789359	0.997402
FBXW5	126.948	-0.38182	-1.40023	0.161445	0.997402
PSMG2	126.6606	0.004101	0.015031	0.988007	0.997464
MTATP6P1	123.7553	-0.15038	-0.55119	0.581501	0.997402
NDUFS3	116.0894	-0.04366	-0.15993	0.872935	0.997402
FASTKD5	116.2351	-0.10932	-0.4004	0.688859	0.997402
WSB2	119.8229	-0.10635	-0.38934	0.697024	0.997402
AKAP9	118.5957	-0.02864	-0.10481	0.916524	0.997402
TAP1	125.9605	0.070388	0.257631	0.796691	0.997402
ZFR	118.6915	-0.1418	-0.51897	0.603783	0.997402
WDR55	117.7653	0.275754	1.008999	0.312975	0.997402
DEK	115.6186	-0.00601	-0.02199	0.982456	0.997402
AC037198	131.9121	-1.87784	-6.8691	6.46E-12	1.12E-09
ORMDL2	116.4809	-0.16606	-0.60719	0.543724	0.997402
ENDOD1	137.2974	-1.29119	-4.71895	2.37E-06	0.00018
USP3	127.0125	0.030781	0.112478	0.910444	0.997402
SIL1	122.4756	0.008303	0.030334	0.975801	0.997402
SLC29A1	119.4359	0.119647	0.436992	0.662117	0.997402
ATP6V1D	128.4166	0.100077	0.365431	0.71479	0.997402
LNPEP	167.6556	0.118476	0.432613	0.665296	0.997402
HYAL3	117.1696	0.556331	2.031273	0.042227	0.552693
MLLT11	116.3192	0.649194	2.370088	0.017784	0.318091
CHID1	117.3501	0.36068	1.31666	0.187953	0.997402
AC044849	115.6583	0.167898	0.612895	0.539945	0.997402
UBASH3B	140.0348	0.216865	0.791535	0.428632	0.997402
PIK3C2B	127.7699	-0.70465	-2.57176	0.010118	0.213451
CSE1L	116.586	0.706933	2.579728	0.009888	0.210446
CXCR4	128.0916	-0.59033	-2.15397	0.031243	0.458692
TRAF3IP3	117.467	0.172083	0.627788	0.530143	0.997402
DCAF11	127.7255	-0.25571	-0.93258	0.351034	0.997402
SNAPIN	117.5391	-0.31572	-1.15134	0.249591	0.997402
EXOSC10	150.5101	0.244712	0.892338	0.372212	0.997402
MED28	117.6035	0.141217	0.514654	0.606795	0.997402
CASC4	114.9321	0.036543	0.133134	0.894088	0.997402
HELZ	116.1981	0.080187	0.292118	0.770196	0.997402
CHCHD3	117.0112	0.159302	0.580031	0.561894	0.997402
CACYBP	119.1834	0.128318	0.467207	0.640352	0.997402

BLVRB	132.4231	1.299156	4.729743	2.25E-06	0.000174
WDR61	114.3159	-0.01207	-0.04391	0.964973	0.997402
HMGN3	118.706	-0.41459	-1.5088	0.131349	0.980846
CNNM2	121.577	0.235614	0.857147	0.391363	0.997402
MTPAP	119.6447	0.386503	1.405424	0.159895	0.997402
SLC25A38	122.8717	-0.00079	-0.00288	0.997705	0.999604
CCNI	133.66	-0.1574	-0.57222	0.56717	0.997402
BFAR	118.9533	-0.03833	-0.13932	0.889195	0.997402
CHMP5	123.3609	7.14E-05	0.00026	0.999793	0.999925
ENOPH1	114.9879	0.495	1.798841	0.072044	0.746201
PPP2R5D	119.9116	-0.06298	-0.22884	0.818994	0.997402
PSME3IP1	116.8321	0.124112	0.450934	0.652037	0.997402
PLD1	114.7862	-0.20087	-0.72968	0.465583	0.997402
CAMK2G	113.629	-0.00681	-0.02471	0.980283	0.997402
NRIP3	158.7268	-1.0813	-3.92326	8.74E-05	0.004248
FLNB	143.5088	-0.25991	-0.94294	0.345713	0.997402
PDLIM7	118.5406	0.192293	0.697514	0.485481	0.997402
MGST3	114.4453	-0.28208	-1.02298	0.306318	0.997402
NR1H2	117.7749	0.13857	0.502469	0.615338	0.997402
PCTP	117.53	-0.71026	-2.57528	0.010016	0.212524
VPS25	128.3518	0.001494	0.005416	0.995678	0.999604
NELFCD	113.5623	-0.07038	-0.25515	0.798608	0.997402
SEC22C	112.591	-0.08409	-0.30481	0.760512	0.997402
GMPS	118.4342	-0.07678	-0.27815	0.780901	0.997402
MLH1	116.4469	-0.0658	-0.23823	0.811699	0.997402
UBE2G2	117.0347	0.08157	0.295283	0.767778	0.997402
ARID1A	119.0704	0.042531	0.153956	0.877644	0.997402
ATG13	115.2308	-0.04824	-0.17459	0.861405	0.997402
KLHL9	119.1388	-0.04116	-0.14886	0.881668	0.997402
SETX	117.4274	0.172978	0.625573	0.531595	0.997402
AL139099.	112.4533	-0.21065	-0.76152	0.446347	0.997402
TGFBR2	114.3167	-0.34509	-1.24748	0.212221	0.997402
SPOP	117.5604	0.559236	2.021482	0.04323	0.559687
IFNGR2	161.9783	0.161609	0.584044	0.559191	0.997402
DGUOK	118.1633	-0.04525	-0.1635	0.870124	0.997402
RC3H1	115.8074	0.586566	2.118959	0.034094	0.485068
SMC3	113.3587	0.038151	0.137695	0.890481	0.997402
MEAF6	112.7451	0.183517	0.66231	0.507772	0.997402
GBA	120.8294	0.119779	0.43222	0.665582	0.997402
ACO1	130.0957	-1.0639	-3.83783	0.000124	0.005762
ELK4	115.5243	-0.5912	-2.13179	0.033024	0.475155
RABAC1	114.8849	-0.45957	-1.65692	0.097536	0.857572
MRPS7	121.9579	0.1881	0.677965	0.497794	0.997402
UBE2A	135.0608	0.311182	1.121491	0.262079	0.997402
IL16	117.0608	-0.14203	-0.51148	0.609015	0.997402
TCHP	117.1326	-0.14324	-0.51554	0.606174	0.997402
NKIRAS2	118.5827	0.201954	0.726809	0.467343	0.997402
DDB2	116.6726	0.811762	2.921419	0.003484	0.094309
EVI2B	114.6978	0.387672	1.394854	0.16306	0.997402
UBAP1	119.8495	-0.0858	-0.30869	0.757556	0.997402

MED8	115.2473	-0.28718	-1.03313	0.301544	0.997402
AGPAT5	112.208	-0.21717	-0.78075	0.434951	0.997402
TRNT1	112.1154	-0.4248	-1.52712	0.12673	0.969929
MNDA	113.9089	-0.59789	-2.14923	0.031616	0.461617
PTCD1	114.0757	0.602778	2.166644	0.030262	0.447696
UBQLN1	116.7141	0.327068	1.175384	0.239841	0.997402
CNTRL	115.6108	0.258289	0.928156	0.353327	0.997402
NTRK1	144.7728	1.086407	3.903557	9.48E-05	0.004575
GRK2	115.3935	0.200299	0.719641	0.471746	0.997402
SCD	114.2323	-0.9314	-3.34526	0.000822	0.029045
ASH2L	115.5227	0.097873	0.351488	0.725222	0.997402
STARD7	119.8673	0.207425	0.744788	0.4564	0.997402
SOGA1	111.5926	-0.40087	-1.43888	0.150185	0.997402
CYLD	128.3532	0.746124	2.677463	0.007418	0.170908
ATOX1	112.8447	-0.1549	-0.55564	0.578455	0.997402
AC009120	110.6631	0.4135	1.481685	0.138424	0.996834
MRPL51	109.8079	-0.13262	-0.4751	0.634716	0.997402
FGD3	116.3029	-0.24801	-0.88844	0.374301	0.997402
SPTLC2	113.9369	-0.38745	-1.3879	0.165167	0.997402
ARHGAP4	148.1572	-0.18086	-0.6474	0.517373	0.997402
ATP5ME	116.911	-0.05223	-0.18692	0.851727	0.997402
MRPS35	117.6146	0.241922	0.865778	0.386612	0.997402
RPS28P7	123.4835	-0.21631	-0.77405	0.438899	0.997402
DDX39B	115.6067	0.057531	0.205746	0.836989	0.997402
EXOC4	127.9719	0.081737	0.292222	0.770117	0.997402
LRP1	111.2116	0.00379	0.013546	0.989192	0.998222
CEBPZ	110.7414	0.081719	0.292019	0.770272	0.997402
SLC30A7	113.7605	0.467605	1.670894	0.094743	0.84543
PPCS	110.6834	0.068349	0.244228	0.807054	0.997402
SLAMF8	113.6616	0.77862	2.781277	0.005415	0.13438
AKR1B1	121.3335	-0.38525	-1.37588	0.168859	0.997402
MCEMP1	115.2117	0.267265	0.954379	0.339892	0.997402
CST3	142.4387	-0.70793	-2.52781	0.011478	0.234539
SCOC	109.7093	0.016049	0.057304	0.954303	0.997402
GALM	111.7858	0.675247	2.410948	0.015911	0.29413
MGAT2	113.304	-0.38753	-1.38355	0.166497	0.997402
CCL3	116.4942	0.733851	2.619889	0.008796	0.194417
AAK1	113.2409	-0.46333	-1.65387	0.098155	0.860129
CAPN2	111.2044	-0.68971	-2.46067	0.013868	0.270179
NDUFAB1	117.7195	0.298814	1.065741	0.286541	0.997402
PTP4A2	116.7127	-0.11979	-0.42723	0.669212	0.997402
LSM14A	111.3549	-0.0912	-0.32526	0.744985	0.997402
MACF1	127.825	0.062256	0.22202	0.824298	0.997402
TLE3	117.1431	-0.7169	-2.55662	0.01057	0.220698
AFF4	117.0358	0.221711	0.790587	0.429185	0.997402
MRPS23	111.4315	-0.06732	-0.24003	0.810309	0.997402
KCP	112.7401	-0.45257	-1.61338	0.106662	0.900642
MCTP2	135.7128	0.041277	0.147147	0.883016	0.997402
TMX2	110.7553	0.212356	0.756793	0.449174	0.997402
FKBP3	113.8737	0.152129	0.542127	0.587731	0.997402

TMEM167	120.2554	0.43184	1.538702	0.123877	0.963152
PHC3	112.4055	-0.18408	-0.6559	0.511891	0.997402
PPA2	112.2795	-0.20688	-0.73693	0.461167	0.997402
OSBPL8	111.6182	-0.42816	-1.52516	0.12722	0.970732
SPRYD3	114.0792	-0.35102	-1.25032	0.211182	0.997402
RNF216	110.6683	0.308042	1.09722	0.272545	0.997402
NFE2	115.0979	1.192546	4.247286	2.16E-05	0.001266
ANKRD17	108.4482	0.023255	0.08277	0.934035	0.997402
AC011511	115.8244	0.837229	2.979913	0.002883	0.081741
TMED9	154.0809	0.078243	0.278465	0.780655	0.997402
ENY2	113.7932	0.037866	0.134763	0.892799	0.997402
EIF2AK1	110.419	0.062012	0.220614	0.825393	0.997402
NIFK	114.8467	0.446876	1.589393	0.111972	0.922675
NBDY	112.5916	0.05624	0.200003	0.841479	0.997402
BAZ2B	126.1312	-0.11787	-0.41912	0.675126	0.997402
GNL3	111.2278	0.425013	1.510708	0.130863	0.979254
TBRG1	117.8151	0.247661	0.880113	0.378798	0.997402
SLC16A3	109.6023	-0.42162	-1.49799	0.134137	0.985755
ERGIC2	116.8922	-0.11473	-0.40752	0.683628	0.997402
ATP5MC1	109.8665	0.410712	1.458186	0.144789	0.997402
RAB1B	116.0657	-0.3565	-1.26555	0.205675	0.997402
CDK5RAP3	114.4341	-0.47634	-1.69035	0.090961	0.830663
TMEM14C	109.4089	-0.41781	-1.48254	0.138197	0.996197
PISD	107.5815	0.235829	0.836783	0.402714	0.997402
ETS2	116.4867	0.996301	3.535122	0.000408	0.016151
TOP2B	115.4319	0.341718	1.212334	0.225384	0.997402
AL135999.	114.6465	0.415156	1.472872	0.140786	0.997402
NEDD8	107.6098	-0.07614	-0.27012	0.78707	0.997402
SMIM12	109.2134	0.377989	1.340842	0.179972	0.997402
CPSF2	108.9383	-0.41957	-1.48827	0.136679	0.990775
CIAO2A	110.9926	0.009092	0.032251	0.974272	0.997402
NDUFB5	111.6659	0.106773	0.378674	0.70493	0.997402
COX20	108.5777	0.31455	1.11539	0.264683	0.997402
MFF	112.5432	-0.62079	-2.20118	0.027723	0.426251
NBAS	110.3591	-0.45184	-1.60203	0.109148	0.914028
NDUFB10	108.1167	-0.03616	-0.1282	0.897994	0.997402
ASH1L	113.5649	0.122715	0.434906	0.663631	0.997402
WDR45B	108.6089	0.2331	0.826093	0.408751	0.997402
NAMPT	111.1783	-0.10013	-0.35484	0.722706	0.997402
BCCIP	108.7967	0.241498	0.855756	0.392133	0.997402
NUP88	107.3286	-0.12336	-0.43676	0.662286	0.997402
BCLAF1	118.0068	-0.25092	-0.88809	0.37449	0.997402
CSK	108.8388	0.240921	0.852685	0.393834	0.997402
CCDC88A	111.5113	-0.69589	-2.46238	0.013802	0.269371
CDC23	120.9221	-0.00127	-0.0045	0.996413	0.999604
RSL24D1	107.0944	0.223256	0.789929	0.429569	0.997402
SSU72	109.031	-0.08665	-0.30639	0.759311	0.997402
ADAM10	128.7556	0.568324	2.009092	0.044527	0.570308
TBC1D14	128.1048	-0.64729	-2.28785	0.022146	0.368544
ANTXR2	125.5526	-1.21672	-4.30042	1.70E-05	0.001041

NDUFB11	113.011	0.085101	0.300777	0.763584	0.997402
CHD1L	118.4798	0.036644	0.129455	0.896998	0.997402
MTFR1L	114.0133	-0.26071	-0.9209	0.357105	0.997402
PBXIP1	107.962	-0.41078	-1.45099	0.146783	0.997402
CPSF3	110.1258	-0.19253	-0.67992	0.496552	0.997402
PRRC2B	108.8825	-0.02983	-0.10535	0.916098	0.997402
HK3	127.2951	0.801804	2.830403	0.004649	0.117881
DROSHA	106.3464	-0.13861	-0.48928	0.62464	0.997402
RRP7A	106.5182	-0.25276	-0.89208	0.372352	0.997402
BBS4	112.5325	-0.71777	-2.53226	0.011333	0.232577
LYN	115.1965	-0.62247	-2.19604	0.028089	0.430249
KLHDC3	107.1648	0.017191	0.060623	0.951659	0.997402
IRF1	106.6814	0.029464	0.103863	0.917278	0.997402
ASB8	118.3785	-0.07139	-0.25164	0.801317	0.997402
CUEDC2	115.4151	-0.30635	-1.07988	0.280194	0.997402
NF2	110.6868	-0.40682	-1.43363	0.151679	0.997402
TBK1	114.0937	0.134742	0.474759	0.634959	0.997402
NMI	105.8539	-0.15315	-0.53947	0.589564	0.997402
YWHAG	108.3018	-0.09399	-0.33107	0.740588	0.997402
CMPK1	106.2732	0.082347	0.289905	0.771889	0.997402
BCAP29	112.5469	0.277623	0.977295	0.328423	0.997402
LSG1	121.8314	-0.06073	-0.21371	0.830772	0.997402
CREG1	111.7282	-0.25955	-0.91321	0.361131	0.997402
HTT	105.9891	0.076082	0.267459	0.789116	0.997402
PFDN1	110.7834	-0.11634	-0.40897	0.682563	0.997402
TACC1	132.2096	-0.15729	-0.55286	0.580357	0.997402
BSDC1	108.6848	-0.225	-0.79069	0.429122	0.997402
MRPL42	106.6945	0.331773	1.165621	0.243768	0.997402
BZW2	111.2499	0.598945	2.103775	0.035398	0.493955
METTL5	107.7169	0.422754	1.484762	0.137607	0.992942
PRKAG1	106.6695	0.27714	0.97331	0.330399	0.997402
HLA-DRB5	115.5294	0.976833	3.430418	0.000603	0.02275
MKLN1	108.9263	-0.09529	-0.33441	0.738074	0.997402
LIMK1	111.1818	0.450121	1.579484	0.114225	0.929942
MRPL49	109.4232	-0.29766	-1.04449	0.29626	0.997402
ARL1	107.8035	-0.14802	-0.51938	0.603496	0.997402
DDX41	109.831	-0.23948	-0.84011	0.400848	0.997402
RALA	113.4036	-0.14091	-0.4943	0.621096	0.997402
WTAP	112.0918	-0.1219	-0.42759	0.668952	0.997402
GNB1	109.7462	-0.01133	-0.03973	0.96831	0.997402
NSFL1C	105.3194	-0.18131	-0.6356	0.525034	0.997402
MADD	121.1976	0.139457	0.488761	0.625011	0.997402
VPS26A	120.7901	0.159725	0.559742	0.575655	0.997402
RAB14	111.3813	0.497087	1.741207	0.081647	0.785533
VPS53	122.2343	-0.18799	-0.65825	0.510379	0.997402
YIPF5	105.1052	-0.09242	-0.32356	0.746268	0.997402
FPGS	146.7616	-0.26517	-0.92803	0.35339	0.997402
SUMO3	112.0938	0.382179	1.337021	0.181216	0.997402
SRSF2	184.5412	-0.53157	-1.85945	0.062963	0.698537
NDUFA12	128.7552	-0.11174	-0.39082	0.695929	0.997402

SNRPB2	113.5898	0.522125	1.825815	0.067878	0.725567
UFD1	108.7921	0.320577	1.120964	0.262303	0.997402
LGALS9	114.7552	1.116321	3.902912	9.50E-05	0.004575
UTP6	107.554	0.114246	0.399304	0.689669	0.997402
LSP1	111.8063	0.417215	1.458044	0.144828	0.997402
IGBP1	115.747	0.302605	1.057307	0.290371	0.997402
ANAPC16	110.2161	-0.09107	-0.31818	0.75035	0.997402
CAVIN2-AS	115.0947	-0.24585	-0.8586	0.390563	0.997402
TRERF1	108.1573	0.383311	1.338417	0.180761	0.997402
HMOX2	107.9873	0.455334	1.5899	0.111857	0.922675
GNAI2	107.3769	0.009806	0.034233	0.972691	0.997402
MSMO1	109.5432	-0.68086	-2.37666	0.01747	0.314445
SMARCC2	112.2346	0.066153	0.23088	0.817408	0.997402
CCNH	105.2799	0.192485	0.67175	0.501743	0.997402
SIGLEC14	104.956	-0.36674	-1.27927	0.200802	0.997402
FUNDC2	106.4976	-0.0045	-0.01568	0.987488	0.997464
DYNLT3	113.9658	-0.22214	-0.77473	0.438497	0.997402
TCTA	104.6383	-0.0387	-0.13495	0.892649	0.997402
DDX27	116.1608	0.015962	0.055647	0.955623	0.997402
KLHL6	115.1703	-0.05615	-0.19572	0.844826	0.997402
LYST	115.2659	0.055093	0.192025	0.847722	0.997402
DHTKD1	109.654	1.149367	4.005787	6.18E-05	0.003144
POLR2L	114.8268	-0.08602	-0.29978	0.764345	0.997402
TRIM25	105.426	-0.47642	-1.66031	0.096852	0.855428
DNTTIP2	108.6067	0.366688	1.277715	0.20135	0.997402
TSR1	113.1229	0.147853	0.51508	0.606497	0.997402
COX8A	105.9641	0.062433	0.217462	0.827848	0.997402
ALS2	120.4502	-0.37519	-1.30662	0.19134	0.997402
CHMP1A	133.8365	-0.04304	-0.14984	0.880887	0.997402
UCHL5	104.1309	0.312144	1.086592	0.277217	0.997402
SENP5	121.1608	0.111244	0.387216	0.698596	0.997402
PA2G4	104.7141	0.406149	1.41322	0.157591	0.997402
OLA1	112.3438	0.129281	0.449771	0.652875	0.997402
SOAT1	105.9351	0.767579	2.67012	0.007582	0.172925
MRPS10	103.7181	0.000803	0.002792	0.997772	0.999604
RPRD2	103.4141	-0.075	-0.26089	0.794181	0.997402
NUDT5	105.9359	0.076759	0.266989	0.789478	0.997402
ST3GAL4	140.0153	2.507013	8.719805	2.79E-18	8.33E-16
POLR2K	106.6807	0.236143	0.820745	0.411791	0.997402
LST1	104.1121	-0.51775	-1.79883	0.072045	0.746201
CARD8	115.0022	-0.63119	-2.19183	0.028392	0.433248
KIAA1191	104.0356	0.201776	0.700613	0.483544	0.997402
P4HA1	137.0846	-0.08662	-0.30073	0.763619	0.997402
GATA1	123.2066	0.336947	1.169452	0.242221	0.997402
AC243829.	118.9182	0.786411	2.729212	0.006349	0.152038
TAF1D	105.2032	0.280219	0.97244	0.330831	0.997402
MON1B	103.9996	0.034828	0.120839	0.903819	0.997402
SMU1	118.5384	-0.06299	-0.21855	0.827004	0.997402
RUNX1	122.3648	1.065163	3.692685	0.000222	0.009588
THEMIS2	103.0136	0.477005	1.653419	0.098246	0.860398

ABCA7	107.2836	-0.0552	-0.19131	0.848282	0.997402
UBAC2	116.7697	0.366663	1.270761	0.203814	0.997402
ECM1	114.0703	-0.84596	-2.93137	0.003375	0.092036
LMBRD1	105.9605	0.284695	0.98631	0.323981	0.997402
BANF1	102.277	0.056055	0.194171	0.846042	0.997402
ZNF451	108.2334	0.043765	0.151598	0.879504	0.997402
API5	140.7469	0.020351	0.070484	0.943808	0.997402
PPP6R3	106.4757	-0.16988	-0.5883	0.556329	0.997402
FGD5-AS1	104.1401	-0.41834	-1.4487	0.147423	0.997402
WDFY2	105.4678	-0.36456	-1.26243	0.206794	0.997402
STARD3NL	111.1336	-0.15367	-0.53203	0.594707	0.997402
KCTD20	137.5521	-0.7205	-2.49439	0.012617	0.251385
PI4KB	112.2718	-0.56366	-1.95133	0.051017	0.621075
CD200R1	113.5936	1.417371	4.906722	9.26E-07	7.86E-05
WAC	114.6297	0.108463	0.375422	0.707347	0.997402
EMC1	111.0472	0.192693	0.666928	0.504818	0.997402
THUMPD1	105.0454	0.035393	0.122482	0.902518	0.997402
TFRC	172.0863	0.495137	1.712299	0.086842	0.809449
GRPEL1	103.4552	0.320088	1.106761	0.268397	0.997402
SDF2	102.9209	0.236764	0.818551	0.413043	0.997402
DCTD	114.0278	-0.2232	-0.77162	0.440337	0.997402
GPBP1	106.8885	-0.01539	-0.05318	0.957588	0.997402
DPP8	103.2106	-0.01192	-0.04116	0.967166	0.997402
MYO19	105.037	0.007997	0.027622	0.977964	0.997402
ACOT13	105.0966	0.536741	1.853872	0.063758	0.704081
ZMPSTE24	106.6183	0.142813	0.493093	0.621947	0.997402
EXOC7	114.2871	-0.31547	-1.08908	0.276117	0.997402
TMEM223	105.6071	-0.13423	-0.46326	0.643182	0.997402
C9orf16	108.5566	-0.14253	-0.49188	0.622802	0.997402
DUSP22	104.2727	-0.59433	-2.05105	0.040262	0.537264
IPO9	110.2675	-0.04053	-0.13988	0.888756	0.997402
NOL8	103.3695	-0.13063	-0.4507	0.652209	0.997402
RNF181	101.8772	0.282115	0.97323	0.330439	0.997402
GNPAT	106.2126	0.44449	1.533348	0.12519	0.968115
DNAJC9	116.6594	-0.27191	-0.93772	0.348387	0.997402
NFAT5	104.4915	-0.45385	-1.56316	0.118014	0.943655
GNAI1	106.7795	-0.70662	-2.43318	0.014967	0.280876
IMP4	121.9329	0.478138	1.646272	0.099708	0.868432
ISCU	103.6561	0.029933	0.103019	0.917948	0.997402
IQGAP2	102.3774	0.22503	0.774245	0.438786	0.997402
GNPTG	103.8645	0.164919	0.56738	0.570456	0.997402
ASRGL1	102.781	-0.70724	-2.43289	0.014979	0.280876
PLOD3	102.9094	0.761228	2.618102	0.008842	0.195137
IL18BP	105.7395	0.836241	2.875519	0.004034	0.105396
ZKSCAN1	102.631	-0.09524	-0.32748	0.743306	0.997402
PPIG	114.6146	0.108315	0.37243	0.709573	0.997402
CYC1	120.1885	-0.26377	-0.90676	0.364536	0.997402
PBDC1	101.0274	-0.3404	-1.16963	0.242151	0.997402
PACSIN2	104.0501	-0.10675	-0.36671	0.713837	0.997402
PI4KA	102.2715	-0.22897	-0.78642	0.431622	0.997402

MED15	100.7912	0.055974	0.192242	0.847553	0.997402
RIPK2	101.539	0.120967	0.415374	0.677868	0.997402
NOC2L	110.37	0.169214	0.580969	0.561261	0.997402
TAPBPL	109.4821	0.421959	1.448489	0.14748	0.997402
UTP18	102.5126	0.106694	0.366254	0.714176	0.997402
FAM136A	101.9074	0.311046	1.06765	0.285679	0.997402
NOLC1	112.6448	0.633761	2.175333	0.029605	0.441921
ASCC2	101.0298	0.426798	1.464919	0.142943	0.997402
BST1	101.0835	-0.25437	-0.87297	0.382677	0.997402
GTF2H1	111.4995	-0.08221	-0.28212	0.777851	0.997402
DGLUCY	110.8589	-0.30591	-1.04946	0.293966	0.997402
VPS37A	103.7927	0.1126	0.386043	0.699465	0.997402
KAT5	100.5212	-0.35723	-1.22471	0.220683	0.997402
RRAGA	125.2884	-0.58734	-2.01351	0.044061	0.565348
ATG12	115.5726	0.427962	1.46662	0.142479	0.997402
CCNL1	104.1444	0.214875	0.736333	0.461528	0.997402
TLDC2	103.4004	-0.98162	-3.36303	0.000771	0.027442
NNT	110.3779	-0.17076	-0.58489	0.558625	0.997402
PYCARD-A!	100.6024	-0.29988	-1.02711	0.304371	0.997402
ABCE1	116.2362	0.093556	0.320335	0.748714	0.997402
RHOT2	113.5636	-0.1102	-0.37727	0.705975	0.997402
CXXC1	105.2588	0.139754	0.478332	0.632414	0.997402
NARF	104.5398	-0.03489	-0.1194	0.904955	0.997402
PLSCR1	110.093	-0.28588	-0.9784	0.327877	0.997402
NIT2	99.54429	0.034797	0.119079	0.905213	0.997402
SLU7	102.5083	0.062572	0.214064	0.830497	0.997402
SLC66A3	103.2429	-0.65906	-2.25456	0.024161	0.392515
CCDC84	99.93256	0.2568	0.878269	0.379798	0.997402
CCL4L2	114.6353	1.350333	4.617614	3.88E-06	0.00028
ITFG1	107.0705	0.248934	0.85122	0.394647	0.997402
HMOX1	104.0033	0.842494	2.880385	0.003972	0.104353
SLC30A9	104.8962	0.038362	0.131103	0.895694	0.997402
PCNP	104.6774	0.183988	0.628769	0.5295	0.997402
DDX56	110.1492	0.267874	0.915189	0.360092	0.997402
MSRB1	107.355	-0.12526	-0.4279	0.668725	0.997402
ADAM17	104.4608	0.246216	0.841063	0.400313	0.997402
AVL9	101.3714	-0.1338	-0.45698	0.647685	0.997402
ADSL	104.728	0.266312	0.909004	0.363348	0.997402
FAM13B	127.9832	-0.50254	-1.71483	0.086376	0.808693
SPG11	102.4791	-0.1327	-0.45274	0.650732	0.997402
DDX3X	147.075	0.242422	0.826906	0.40829	0.997402
GRHPR	106.4481	0.198569	0.677211	0.498272	0.997402
DNAJA3	100.5544	0.193043	0.658287	0.510354	0.997402
CARS1	101.8181	-0.01537	-0.05234	0.958255	0.997402
NKTR	109.8012	0.039999	0.136253	0.891622	0.997402
TIMM17A	100.5826	0.500781	1.705261	0.088146	0.815775
CD22	107	-0.5134	-1.74758	0.080537	0.783898
H3-3A	102.2455	-0.31312	-1.0656	0.286605	0.997402
RNF40	98.27503	-0.01612	-0.05484	0.956266	0.997402
ABCF2	98.80666	0.115434	0.392665	0.694567	0.997402

TARDBP	98.19924	-0.11787	-0.40081	0.688562	0.997402
MAX	100.1238	-0.28698	-0.97567	0.329229	0.997402
DOK2	103.3457	0.960149	3.263357	0.001101	0.036356
AC135048	109.7305	-0.30537	-1.03789	0.299319	0.997402
HECTD1	98.2525	-0.07445	-0.25301	0.800261	0.997402
KLHL20	104.9413	-0.46414	-1.57602	0.115021	0.932432
AC067930	98.81289	0.114442	0.388525	0.697628	0.997402
COPS4	114.4107	0.021601	0.073317	0.941554	0.997402
NISCH	100.3885	0.34366	1.166194	0.243536	0.997402
ALG5	101.5715	-0.25433	-0.86291	0.388184	0.997402
SMS	100.256	0.305854	1.037655	0.299431	0.997402
NAA25	106.7906	-0.00085	-0.00289	0.997692	0.999604
SCYL1	104.4673	-0.08978	-0.30451	0.760737	0.997402
TMF1	97.60506	0.137644	0.466703	0.640713	0.997402
SPATA20	101.8924	-0.52435	-1.77761	0.075469	0.757442
STARD4	100.2055	-0.4518	-1.53152	0.12564	0.968243
NUP62	101.3824	0.125049	0.423738	0.671757	0.997402
WDR13	109.8721	-0.59156	-2.00406	0.045064	0.573598
PICALM	106.5019	1.224865	4.149491	3.33E-05	0.001811
NDUFAF3	107.0947	-0.38891	-1.31731	0.187734	0.997402
EXOC6	102.1013	0.120012	0.406483	0.684388	0.997402
ZNF330	100.3217	0.126924	0.429701	0.667413	0.997402
SF1	98.74482	0.458909	1.553542	0.120294	0.951775
SELL	122.4598	0.364682	1.234379	0.217062	0.997402
SLC25A5-A	97.75691	0.41796	1.414668	0.157166	0.997402
NAA50	108.3171	0.46255	1.565546	0.117455	0.941805
PPHLN1	101.1846	0.044617	0.15097	0.879999	0.997402
BMP2K	109.9774	-0.97745	-3.3072	0.000942	0.031957
MED12	100.4145	-0.2519	-0.85209	0.394162	0.997402
SLC38A2	106.403	-1.07495	-3.63557	0.000277	0.011702
GNL2	102.1421	0.402313	1.360549	0.173656	0.997402
DRG1	108.953	-0.19073	-0.64488	0.519008	0.997402
SPG21	102.1397	-0.01277	-0.04319	0.965552	0.997402
CHD7	105.979	-0.35158	-1.18861	0.234594	0.997402
RPL10P9	101.2	-0.07174	-0.24248	0.808409	0.997402
RIN3	109.9331	-0.52679	-1.77962	0.075138	0.756394
MLEC	100.5302	-0.38744	-1.30882	0.190594	0.997402
TPGS2	99.54136	-0.24333	-0.82194	0.411109	0.997402
IL10RB	103.195	-0.25388	-0.85753	0.391154	0.997402
PIM2	108.1902	1.693048	5.718556	1.07E-08	1.21E-06
MICU1	108.5932	0.191662	0.647329	0.517419	0.997402
TMEM138	103.3745	0.051819	0.174983	0.861093	0.997402
IARS2	100.1829	0.030067	0.101528	0.919132	0.997402
TANGO2	102.9219	0.193054	0.651774	0.514547	0.997402
NUDT19	104.7015	0.003626	0.012242	0.990232	0.99849
PRELID3B	105.137	0.667295	2.252405	0.024297	0.39351
BLMH	102.4552	-0.10602	-0.35776	0.72052	0.997402
TRAF7	99.98407	0.006003	0.020259	0.983837	0.997402
TP53BP1	97.30398	-0.0318	-0.10729	0.91456	0.997402
CLNK	99.81106	0.915696	3.088949	0.002009	0.059602

DPM2	96.53577	-0.02454	-0.08277	0.934038	0.997402
CCDC90B	101.6099	-0.02549	-0.08595	0.931507	0.997402
EIF2B4	100.4675	0.107963	0.363949	0.715896	0.997402
INTS14	101.3492	-0.3204	-1.08008	0.280104	0.997402
INTS11	98.45814	-0.22814	-0.76901	0.441885	0.997402
BTF3L4	102.2497	0.069447	0.234025	0.814965	0.997402
NIPBL	96.71351	-0.15064	-0.50755	0.611766	0.997402
B4GALT3	100.4384	-0.13467	-0.45369	0.650055	0.997402
ARGLU1	98.37625	0.055412	0.186666	0.851923	0.997402
TRIM21	99.85352	0.302602	1.019105	0.308153	0.997402
RTCA	99.24614	-0.17571	-0.59176	0.554012	0.997402
NUP188	96.47647	0.294614	0.992161	0.321119	0.997402
MRPS18B	101.8783	-0.09104	-0.3066	0.759148	0.997402
SPTLC1	104.9973	-0.11475	-0.38642	0.699187	0.997402
AC098848	97.22283	-0.52909	-1.78165	0.074806	0.755887
CCAR2	103.7474	0.072134	0.242895	0.808087	0.997402
PRKCSH	102.8629	0.364098	1.225962	0.220213	0.997402
ARF5	103.1197	-0.56835	-1.91358	0.055674	0.654182
GAK	97.4699	0.232286	0.781816	0.434323	0.997402
ADSS2	101.8985	0.311996	1.049557	0.293922	0.997402
DENND1B	104.8882	1.021361	3.435504	0.000591	0.022386
GDI1	103.6456	0.254845	0.856805	0.391553	0.997402
SZRD1	101.1821	0.086947	0.292319	0.770043	0.997402
ST8SIA1	105.9369	-1.41043	-4.7409	2.13E-06	0.000166
PCNA	98.68626	1.74E-05	5.84E-05	0.999953	0.999953
TBRG4	99.48726	0.243076	0.816748	0.414072	0.997402
ELOVL6	104.5081	-0.64182	-2.15649	0.031046	0.456299
MKKS	97.14622	0.593883	1.995329	0.046007	0.582498
C2orf68	97.35822	-0.36455	-1.2248	0.220652	0.997402
LIMK2	104.8882	-0.34953	-1.17423	0.240304	0.997402
TNPO3	96.12799	0.138243	0.464374	0.64238	0.997402
MCRS1	101.7588	-0.17034	-0.57191	0.567383	0.997402
POLR3H	101.0366	-0.02349	-0.07887	0.937136	0.997402
OPTN	96.34401	0.087167	0.292533	0.769879	0.997402
PPP1R7	102.9289	0.493748	1.656797	0.097561	0.857572
RNF34	102.9562	-0.29081	-0.97562	0.329251	0.997402
CPQ	96.7472	-0.5479	-1.83764	0.066115	0.715791
CLEC4O	102.9988	-1.2347	-4.14096	3.46E-05	0.001844
ATG4B	101.2699	0.35946	1.205543	0.227994	0.997402
TTC1	101.4472	0.223538	0.749658	0.453461	0.997402
GPR34	97.20585	-0.0155	-0.05199	0.958533	0.997402
PHACTR1	97.22576	-0.42239	-1.41636	0.156669	0.997402
ZNF655	98.91958	0.112584	0.377506	0.705797	0.997402
PON2	97.03439	0.079214	0.265601	0.790547	0.997402
DELE1	109.654	-0.02001	-0.0671	0.946506	0.997402
DDIT3	98.48635	-0.214	-0.71697	0.473395	0.997402
TM2D2	96.22963	-0.13709	-0.45921	0.646085	0.997402
IER3	114.6483	2.060453	6.900135	5.20E-12	9.20E-10
MRPS34	98.826	0.218141	0.730348	0.465177	0.997402
RPAIN	95.85795	0.070909	0.237304	0.812421	0.997402

AC022400	96.69584	-0.14914	-0.49891	0.617842	0.997402
PDCD10	105.4277	0.136365	0.456153	0.64828	0.997402
CCDC47	98.83234	-0.06104	-0.20407	0.838297	0.997402
CUL2	95.11724	0.064295	0.21495	0.829806	0.997402
ACADM	94.95145	0.268254	0.896559	0.369954	0.997402
PIK3R5	103.3076	0.917015	3.063605	0.002187	0.063504
AAGAB	95.77302	0.318337	1.063433	0.287585	0.997402
NOL11	97.61017	0.049434	0.165133	0.86884	0.997402
VPS13C	105.822	-0.05373	-0.17939	0.857631	0.997402
MFSD11	120.2647	-0.22892	-0.76433	0.444673	0.997402
NDUFB6	94.69287	-0.21525	-0.71866	0.472351	0.997402
ZNF622	95.88843	0.055902	0.186616	0.851962	0.997402
NDUFB1	98.71098	-0.53454	-1.78386	0.074447	0.755855
PLLP	102.2515	-0.21205	-0.7073	0.479379	0.997402
SMIM29	101.2049	-0.68682	-2.29003	0.022019	0.367716
TOLLIP	106.9217	0.10639	0.354678	0.722831	0.997402
PNPLA6	107.0755	0.840188	2.799462	0.005119	0.128372
DOCK11	108.9905	-0.91506	-3.04891	0.002297	0.06656
NUDT21	96.15555	-0.23989	-0.79883	0.424388	0.997402
HNRNPU	116.4697	-0.14997	-0.49922	0.617623	0.997402
PGS1	96.95576	-0.26273	-0.87453	0.38183	0.997402
COMMD7	98.21378	-0.35793	-1.19125	0.233557	0.997402
SHC1	94.21393	0.21617	0.719339	0.471932	0.997402
TSEN15	98.32851	-0.09071	-0.30168	0.762893	0.997402
MAD1L1	95.72038	-0.19854	-0.66015	0.50916	0.997402
GAB3	94.7233	-0.19973	-0.66387	0.506771	0.997402
IFRD2	94.07033	0.050337	0.167294	0.867139	0.997402
RBM10	95.5247	0.343022	1.139992	0.25429	0.997402
VPS11	97.51782	-0.18668	-0.62022	0.535113	0.997402
LSM8	94.0597	0.018919	0.062841	0.949893	0.997402
GTF3C2	93.91259	0.194972	0.647466	0.51733	0.997402
DOCK10	122.1234	-0.69527	-2.30798	0.021	0.356306
SEH1L	105.5593	0.256156	0.850266	0.395177	0.997402
DHPS	94.09568	0.470618	1.561895	0.118313	0.944987
SLC35B1	93.98004	0.317451	1.053503	0.292111	0.997402
CRLF3	96.8644	0.027137	0.09005	0.928247	0.997402
UBR2	107.1298	0.102412	0.339841	0.733976	0.997402
DERL2	94.91438	0.269814	0.895264	0.370646	0.997402
IKBKE	93.94075	0.163479	0.542333	0.587589	0.997402
BTN2A2	106.1083	-1.27758	-4.23712	2.26E-05	0.001299
CASC3	93.34993	0.026609	0.088236	0.929689	0.997402
ACTN1	104.293	-0.01555	-0.05156	0.958877	0.997402
GCC1	95.10703	-0.32024	-1.06146	0.288482	0.997402
AGO2	95.80603	0.50316	1.667617	0.095392	0.846255
USP47	101.0602	-0.06432	-0.21312	0.831234	0.997402
SYT11	96.47761	-0.66746	-2.21111	0.027028	0.422709
FTSJ1	98.28026	-0.26832	-0.88882	0.3741	0.997402
TMED5	109.4938	-0.1625	-0.53807	0.590526	0.997402
BABAM2	100.3179	-0.08973	-0.29711	0.766381	0.997402
APOOL	99.79156	1.061606	3.515089	0.00044	0.017277

FADS2	137.7591	-1.04172	-3.44887	0.000563	0.021534
MIEF1	100.501	0.196448	0.65028	0.515511	0.997402
SRA1	97.57787	-0.02608	-0.08627	0.93125	0.997402
DENND4C	113.853	-0.42196	-1.39597	0.162724	0.997402
YIPF4	94.38898	0.2088	0.690652	0.489785	0.997402
TMEM39A	93.55045	-0.04287	-0.14178	0.887256	0.997402
HIGD2A	109.4854	0.338417	1.118962	0.263156	0.997402
KDM4A	94.9063	-0.35933	-1.18774	0.234937	0.997402
ALG3	95.63879	0.137858	0.455668	0.648629	0.997402
MRPL15	96.67577	0.348701	1.152569	0.249087	0.997402
SLC35F6	94.63141	-0.76846	-2.53965	0.011096	0.229032
DUSP3	92.731	-0.18945	-0.62607	0.531269	0.997402
ARHGAP9	93.463	-0.10257	-0.33897	0.734629	0.997402
PIP4K2C	94.34326	-0.04964	-0.16406	0.869685	0.997402
COG2	93.98969	-0.54177	-1.78995	0.073461	0.753793
AL391988.	100.1811	-1.19291	-3.94029	8.14E-05	0.004026
NUDT22	95.59276	0.21613	0.71358	0.475487	0.997402
TFIP11	92.68341	-0.20452	-0.67525	0.499519	0.997402
OSGEP	96.81777	-0.10803	-0.35663	0.72137	0.997402
TRAPPC4	92.33441	-0.02065	-0.06817	0.945654	0.997402
PARL	92.38505	0.119044	0.392822	0.694451	0.997402
TMEM208	95.04215	0.064549	0.212979	0.831343	0.997402
ERO1A	97.10371	-0.03974	-0.13109	0.895705	0.997402
PTPN18	101.0107	-0.65013	-2.1445	0.031993	0.464775
AL121748.	107.749	-0.23996	-0.79123	0.428808	0.997402
RAE1	94.24831	0.338122	1.114908	0.26489	0.997402
EHD4	95.74878	-0.07849	-0.25874	0.795834	0.997402
NDST1	92.75399	-0.175	-0.57681	0.564071	0.997402
UROD	97.05941	-0.15917	-0.52453	0.59991	0.997402
UBE2V2	96.81162	0.314217	1.035418	0.300474	0.997402
ZDHHC6	97.68759	0.387924	1.278091	0.201217	0.997402
MYL5	92.46132	-0.24537	-0.80837	0.418875	0.997402
ANKRD52	97.27137	-0.07743	-0.25501	0.798712	0.997402
TCF12	97.26391	0.225174	0.741476	0.458405	0.997402
HIGD1A	98.3853	0.547487	1.802725	0.071431	0.744142
NMD3	93.99502	0.362392	1.192731	0.232975	0.997402
RPL7L1	99.71326	0.159455	0.524766	0.599746	0.997402
WIPF1	92.16586	-0.28478	-0.93696	0.34878	0.997402
AIMP2	92.2864	0.408155	1.341782	0.179667	0.997402
NAA60	91.45328	0.182211	0.598895	0.549243	0.997402
GRWD1	93.27897	0.326961	1.074554	0.282574	0.997402
UTP4	95.05607	0.140499	0.461718	0.644284	0.997402
PCSK7	91.45338	0.197902	0.650341	0.515472	0.997402
MRPS22	96.16516	0.151611	0.498031	0.618462	0.997402
ZNF146	114.3304	0.195071	0.640667	0.521739	0.997402
DPP9	111.278	0.407488	1.338112	0.18086	0.997402
U2SURP	94.39648	0.026163	0.085912	0.931536	0.997402
NDUFB7	91.33242	0.026473	0.086928	0.930729	0.997402
VPS4B	105.638	0.158549	0.520586	0.602655	0.997402
MAN2B1	152.5767	0.101147	0.331977	0.739906	0.997402

CSNK1D	95.13066	0.064991	0.213304	0.83109	0.997402
DCAF5	92.34596	-0.0057	-0.01872	0.985062	0.997402
RAB34	104.8703	-0.71183	-2.33572	0.019506	0.340821
DNMT1	114.1616	0.238294	0.781872	0.43429	0.997402
CBFB	92.00729	0.243866	0.799764	0.423848	0.997402
ACAA2	93.37808	0.227426	0.745797	0.45579	0.997402
GMFB	97.81797	0.327781	1.074814	0.282458	0.997402
NAPA	92.37127	0.02504	0.08208	0.934583	0.997402
PTPN2	93.20093	0.731201	2.396437	0.016555	0.303887
PADI4	105.7423	-0.04304	-0.14103	0.887847	0.997402
RNF6	102.2949	0.100614	0.329684	0.741639	0.997402
DCTPP1	93.08579	0.411552	1.348456	0.177512	0.997402
NOB1	95.3902	0.74008	2.423693	0.015364	0.286594
SCAMP3	97.47866	0.127474	0.417421	0.67637	0.997402
MAGED1	92.31468	-0.38452	-1.25894	0.208053	0.997402
FOSL2	91.52398	-0.20168	-0.66025	0.509093	0.997402
DENND4B	92.82613	0.533697	1.747051	0.080628	0.783898
DDX23	104.4703	-0.01689	-0.0553	0.955902	0.997402
GTF2F1	97.32063	0.265625	0.869379	0.38464	0.997402
PYGB	93.98392	-0.4307	-1.40925	0.158762	0.997402
DMTF1	94.40746	0.072251	0.236341	0.813168	0.997402
GALNT2	93.32858	-0.4765	-1.5585	0.119115	0.947592
FRY	108.8848	-0.43065	-1.40841	0.159011	0.997402
PTPN1	94.05344	-0.36806	-1.2034	0.228823	0.997402
NR3C1	95.84066	-0.74749	-2.44291	0.01457	0.276821
ANKRD12	105.0224	0.073886	0.241421	0.809229	0.997402
NPRL2	93.25195	-0.2828	-0.92401	0.355482	0.997402
VAC14	101.1687	-1.14679	-3.74623	0.00018	0.007924
TRAM1	96.72568	-0.45661	-1.49122	0.135903	0.990539
RUVBL2	91.92091	0.228727	0.746869	0.455143	0.997402
ROMO1	97.7994	0.264376	0.862999	0.388138	0.997402
FAT1	95.86036	-0.78017	-2.54646	0.010882	0.225884
NPC1	112.1464	0.34679	1.131887	0.257682	0.997402
STXBP2	103.633	-0.23594	-0.76999	0.441305	0.997402
TRIM32	95.96215	-0.84955	-2.77232	0.005566	0.136715
GET3	90.86818	0.120894	0.394424	0.693268	0.997402
ENOSF1	93.32827	-0.28688	-0.93595	0.349301	0.997402
PLOD1	90.60943	0.065282	0.212947	0.831369	0.997402
ABCC1	103.1091	-0.65306	-2.13018	0.033157	0.47659
CIAPIN1	93.70701	0.09422	0.30731	0.758607	0.997402
MRPL33	95.36981	0.012193	0.039749	0.968293	0.997402
MAN2A2	90.24632	-0.13203	-0.4303	0.666981	0.997402
WDR46	95.46723	0.219767	0.716225	0.473852	0.997402
PIK3CD	110.7283	0.296095	0.964979	0.334556	0.997402
ZNF333	91.80305	-0.43563	-1.41958	0.155731	0.997402
SYF2	90.43164	0.072534	0.236341	0.813168	0.997402
BLOC1S2	105.1111	0.168388	0.548655	0.583242	0.997402
P2RX4	92.04803	-0.47472	-1.54622	0.122052	0.955174
NCOR2	95.98173	-0.31341	-1.02081	0.307344	0.997402
RPL39	105.6914	0.139855	0.455442	0.648791	0.997402

RSAD2	90.18383	0.096899	0.315474	0.752402	0.997402
TMEM43	98.44467	0.094499	0.307568	0.758411	0.997402
S100B	100.0231	1.277827	4.158365	3.21E-05	0.001755
ADPRH	91.93703	-0.24507	-0.79745	0.425187	0.997402
CIB1	92.88818	-0.30384	-0.98856	0.322878	0.997402
SLC25A44	90.16757	0.080242	0.260991	0.7941	0.997402
XIAP	91.56648	-0.60822	-1.97816	0.047911	0.597639
GRIPAP1	89.49744	0.265638	0.863829	0.387682	0.997402
MED29	91.5776	-0.13967	-0.45416	0.649713	0.997402
CNRIP1	93.76726	0.448763	1.458338	0.144747	0.997402
NUDT3	106.6626	-0.18454	-0.59938	0.548918	0.997402
ZNF292	89.21034	0.210278	0.682927	0.494653	0.997402
WDR83OS	89.37911	-0.00658	-0.02136	0.982956	0.997402
PLAC4	96.38582	0.391313	1.270472	0.203917	0.997402
PPP1R18	96.87458	0.278551	0.904336	0.365817	0.997402
KIF22	92.46739	-0.152	-0.49347	0.621683	0.997402
STARD3	89.98735	0.296566	0.962649	0.335724	0.997402
PPIL2	91.62448	0.160609	0.521292	0.602163	0.997402
SNHG16	89.93888	0.631216	2.047761	0.040583	0.538632
RPL26	90.49326	0.344393	1.117204	0.263907	0.997402
BET1L	91.02916	-0.1083	-0.3513	0.725364	0.997402
FAM120A	91.14297	0.599803	1.945217	0.051749	0.626445
GRSF1	103.8687	-0.53876	-1.74688	0.080658	0.783898
TTC31	91.23667	-0.05044	-0.16351	0.870118	0.997402
HS6ST1	105.5292	-0.20287	-0.65766	0.510755	0.997402
ABCF1	89.3539	0.058437	0.189437	0.84975	0.997402
UTS2	96.88901	1.575479	5.106675	3.28E-07	3.01E-05
ACAT1	90.25193	0.20679	0.670231	0.50271	0.997402
LSM3	90.48776	-0.21861	-0.70846	0.478661	0.997402
PRF1	95.29658	0.568695	1.842734	0.065368	0.711459
TRAPPC3	91.19976	-0.30365	-0.98391	0.325162	0.997402
TOB1	90.04668	-0.48535	-1.57207	0.115935	0.936653
CHMP3	92.68047	-0.07928	-0.25676	0.797362	0.997402
COPS8	93.57531	-0.19742	-0.63911	0.522754	0.997402
HMG20A	101.988	0.23939	0.774898	0.4384	0.997402
WASHC5	102.8462	0.127734	0.413444	0.679281	0.997402
MYC	94.54526	1.338754	4.33303	1.47E-05	0.000934
STX7	101.7405	0.059514	0.192582	0.847286	0.997402
AASDHPPT	96.9285	0.356233	1.15264	0.249058	0.997402
HTATIP2	93.00511	-0.50077	-1.62019	0.105191	0.895172
RHOBTB3	93.26775	-0.14119	-0.45679	0.647825	0.997402
ITGB1BP1	106.0861	-0.68073	-2.20226	0.027647	0.426251
ALG2	89.20208	0.242723	0.784867	0.432532	0.997402
MGRN1	89.85199	0.53274	1.72257	0.084966	0.802351
ABCB9	92.10685	0.174262	0.5633	0.573231	0.997402
NORAD	89.99604	-0.71936	-2.32397	0.020127	0.345534
ROCK1	127.1802	-0.38849	-1.25501	0.209475	0.997402
RAD54L2	89.0471	-0.0455	-0.14696	0.883162	0.997402
TMOD3	90.69566	0.256611	0.828755	0.407243	0.997402
GPAT3	101.363	1.646372	5.315885	1.06E-07	1.07E-05

BDP1	89.35648	-0.08673	-0.28001	0.779473	0.997402
SCPEP1	95.31745	0.254571	0.821646	0.411278	0.997402
MGA	91.82967	-0.035	-0.11295	0.910069	0.997402
STIM1	92.65902	-0.85523	-2.76	0.00578	0.140774
TMC8	95.95746	-0.19119	-0.61687	0.537322	0.997402
NIPSNAP2	97.54157	-0.17414	-0.56181	0.574243	0.997402
ANAPC13	92.03637	0.081808	0.263923	0.791839	0.997402
PHF20	91.97696	0.086047	0.277547	0.78136	0.997402
CD58	98.28448	0.507932	1.638205	0.101379	0.877129
COMMD5	88.40801	0.090718	0.292462	0.769934	0.997402
PTGS2	93.85435	-1.00269	-3.23242	0.001227	0.039304
CRLF2	97.20959	-1.27344	-4.10473	4.05E-05	0.002134
HDAC3	90.81811	-0.13344	-0.42999	0.667205	0.997402
IREB2	96.68209	0.472059	1.520978	0.128265	0.972853
NAXE	89.50663	-0.14239	-0.45875	0.646413	0.997402
SLFN5	154.9346	-1.12564	-3.62631	0.000288	0.012095
MPC2	88.67531	0.163616	0.526998	0.598195	0.997402
DPP3	91.21702	-0.05009	-0.16124	0.871906	0.997402
TMED8	93.56137	-0.96114	-3.09375	0.001976	0.058944
ETHE1	92.26753	-0.25577	-0.82299	0.410512	0.997402
INTS10	88.10109	0.417493	1.343291	0.179178	0.997402
PLXNA2	91.11932	-0.99257	-3.19323	0.001407	0.04397
TRIP12	99.62986	0.078999	0.254072	0.79944	0.997402
PLAA	97.32235	0.11827	0.380351	0.703685	0.997402
MCM3AP	88.02023	0.285253	0.91727	0.359001	0.997402
MAN1B1	87.89025	0.058505	0.188126	0.850778	0.997402
ADCYAP1	92.17738	-1.1445	-3.67995	0.000233	0.010049
KMT2D	89.75687	-0.05297	-0.17029	0.864779	0.997402
RETREG3	111.4572	0.052148	0.167606	0.866893	0.997402
STAB1	94.10928	-0.38406	-1.23398	0.217212	0.997402
DENND5A	88.38907	-0.0408	-0.13108	0.895714	0.997402
C1QC	88.77925	0.784339	2.519813	0.011742	0.238576
PTBP3	88.01427	-0.24309	-0.78094	0.434835	0.997402
PGLS	91.94111	0.195898	0.629265	0.529176	0.997402
PNPLA8	90.85066	-0.13221	-0.42462	0.671113	0.997402
UBE4A	106.2939	-0.06696	-0.21501	0.829763	0.997402
SAP30BP	88.97068	0.105474	0.338635	0.734885	0.997402
PRPF38B	97.58415	0.165371	0.530869	0.59551	0.997402
RNF168	87.58363	0.436407	1.400638	0.161322	0.997402
ST3GAL3	88.79687	-0.58495	-1.877	0.060518	0.686306
SUCLA2	97.93566	0.439035	1.408763	0.158905	0.997402
VEZT	89.90482	0.026064	0.083629	0.933351	0.997402
RPS19BP1	94.05924	0.219549	0.704343	0.481219	0.997402
HP1BP3	91.49743	0.166725	0.534713	0.592848	0.997402
CUL1	87.28313	-0.15526	-0.49792	0.618539	0.997402
PBRM1	87.93506	0.107131	0.34349	0.73123	0.997402
PSMD10	89.50678	-0.15882	-0.50917	0.61063	0.997402
MYB	87.87337	0.392532	1.258387	0.208252	0.997402
DCUN1D1	92.2857	-0.20647	-0.66188	0.508051	0.997402
GLE1	92.97531	0.055452	0.177713	0.858948	0.997402

HSPH1	87.85152	-0.13906	-0.44553	0.655936	0.997402
ARFGAP3	89.87257	-0.00777	-0.0249	0.980137	0.997402
WWP2	91.41069	0.295043	0.94512	0.344597	0.997402
NAT9	92.22751	0.062227	0.199327	0.842007	0.997402
VPS13D	97.04794	-0.97208	-3.11223	0.001857	0.055957
CYB561D2	94.08564	-0.19997	-0.64021	0.522034	0.997402
CREB1	87.95506	0.040462	0.129532	0.896937	0.997402
GFM2	87.38981	-0.07434	-0.23799	0.81189	0.997402
PPP1CC	88.52445	-0.73349	-2.34755	0.018897	0.332612
NDUFC1	87.20692	-0.25312	-0.81003	0.417922	0.997402
RNF14	99.76238	-0.14365	-0.45936	0.645977	0.997402
PNKD	91.74379	0.027665	0.088463	0.929509	0.997402
ITFG2	87.57756	0.420468	1.344023	0.178941	0.997402
LRRC25	89.20269	0.176675	0.564641	0.572318	0.997402
KIDINS220	88.46995	-0.27318	-0.87301	0.382656	0.997402
FNDC3B	95.63324	0.916323	2.928129	0.00341	0.092823
ENTPD5	89.68909	-0.64348	-2.05593	0.039789	0.533754
COA4	86.73138	0.197639	0.631431	0.527758	0.997402
NSA2	113.9047	0.418679	1.337143	0.181176	0.997402
IRAK1	92.12313	-0.67257	-2.14777	0.031732	0.462602
MFAP1	88.08753	0.067368	0.215078	0.829706	0.997402
ARF6	98.90485	-0.0762	-0.24326	0.807807	0.997402
DTD1	85.89538	-0.02568	-0.08196	0.934678	0.997402
LILRB1	101.5512	0.485599	1.549909	0.121163	0.952899
DDX46	88.26971	0.212001	0.676652	0.498627	0.997402
TATDN1	93.32881	0.102294	0.326374	0.744141	0.997402
IGSF6	93.57445	1.529359	4.877171	1.08E-06	8.98E-05
GUCD1	85.98562	-0.2834	-0.90373	0.36614	0.997402
MROH1	88.52093	-0.02918	-0.09303	0.925883	0.997402
MVD	88.14755	-0.41591	-1.32607	0.184817	0.997402
DNAJB11	95.14725	-0.76567	-2.44088	0.014651	0.278007
CTBS	88.17432	-0.0158	-0.05034	0.95985	0.997402
TMEM150	89.82448	-0.71457	-2.27643	0.02282	0.374121
SMIM15	91.60216	-0.18648	-0.59401	0.552503	0.997402
TSNAX	90.64008	-0.56607	-1.80297	0.071394	0.744142
RBM12	86.55683	0.288782	0.919696	0.357732	0.997402
ZCCHC17	88.50917	0.121303	0.386262	0.699302	0.997402
AL031714.	88.44493	-0.17457	-0.55588	0.57829	0.997402
ITGAL	120.5896	1.358718	4.326193	1.52E-05	0.000959
CROT	103.3984	-0.01448	-0.04606	0.96326	0.997402
SNX3	93.99879	-0.05814	-0.185	0.853232	0.997402
MED10	86.37911	0.05911	0.188025	0.850857	0.997402
AFF1	92.59433	0.094684	0.300843	0.763534	0.997402
PIAS3	98.91293	-0.69609	-2.21146	0.027004	0.422709
MSH6	94.67772	-0.01458	-0.04631	0.963062	0.997402
PLCG2	85.72981	-0.3102	-0.98491	0.32467	0.997402
MAOB	130.2283	-0.8587	-2.72559	0.006419	0.153206
RNASE2	111.1381	-0.47366	-1.50322	0.132783	0.983401
MCTP1	103.8633	-0.77523	-2.4602	0.013886	0.270179
MRTFA	84.98361	0.141613	0.449301	0.653214	0.997402

LRMP	90.99536	-0.88271	-2.80031	0.005105	0.128261
OIP5-AS1	85.37463	0.025397	0.080557	0.935794	0.997402
ARRDC1	85.09293	0.37924	1.202856	0.229032	0.997402
CLEC16A	87.70416	0.049459	0.156848	0.875365	0.997402
ZNF3	87.06604	0.018435	0.058441	0.953397	0.997402
SNX11	94.34133	0.302086	0.957223	0.338455	0.997402
FBXW2	85.31818	-0.14235	-0.45095	0.652029	0.997402
DMXL2	98.60307	-0.50343	-1.59419	0.110893	0.919407
SFPQ	91.9396	-0.03765	-0.11921	0.905111	0.997402
STAT2	99.72608	-0.28917	-0.91559	0.35988	0.997402
OGA	97.27513	-0.21071	-0.66715	0.504676	0.997402
MRPL13	87.8151	0.292666	0.926542	0.354164	0.997402
RAB31	85.47904	0.225629	0.714182	0.475115	0.997402
TSR2	87.62609	-0.31613	-1.00058	0.31703	0.997402
REX1BD	87.78949	0.010611	0.03358	0.973212	0.997402
SFXN1	85.05895	0.311651	0.985959	0.324153	0.997402
RBM18	84.85949	0.353684	1.118894	0.263185	0.997402
C19orf53	87.31442	-0.04055	-0.12827	0.897937	0.997402
TRAP1	84.69934	0.014878	0.04705	0.962473	0.997402
TRPC4AP	91.61526	-0.89984	-2.84454	0.004448	0.114247
WDR48	86.74444	0.265963	0.840746	0.40049	0.997402
ULK3	87.02969	-0.3648	-1.15319	0.248832	0.997402
ABHD14B	90.10084	-0.01573	-0.04971	0.960354	0.997402
SPPL2A	94.89365	-0.51728	-1.63506	0.102037	0.880173
PHKG1	84.19829	0.032163	0.101643	0.91904	0.997402
RPUSD3	86.63831	0.150078	0.47414	0.6354	0.997402
PRPF31	84.31632	0.270214	0.853667	0.393289	0.997402
OGG1	87.10718	0.934227	2.951009	0.003167	0.087545
RCSD1	96.35391	-0.4475	-1.4132	0.157596	0.997402
PAN3	88.12752	0.54965	1.735595	0.082636	0.789745
PPP1R2	88.15947	0.534877	1.68853	0.09131	0.830663
ITPA	88.25302	-0.11806	-0.37255	0.709484	0.997402
C7orf50	95.9237	-0.40569	-1.27983	0.200605	0.997402
HMGXB3	88.95042	0.17192	0.542228	0.587661	0.997402
TSTD1	85.8924	-0.48639	-1.53404	0.125021	0.967846
ZNF267	84.39785	0.008102	0.025551	0.979616	0.997402
SUOX	91.71802	0.044694	0.140915	0.887937	0.997402
MAP3K1	93.37379	1.122223	3.538209	0.000403	0.016053
RIOK1	85.21477	0.115626	0.364431	0.715536	0.997402
SETD3	85.01105	0.159144	0.501578	0.615964	0.997402
NECAP1	84.51061	0.10773	0.339505	0.73423	0.997402
MXD3	94.88554	-0.34621	-1.091	0.275275	0.997402
DCP2	108.5134	0.078457	0.247178	0.804771	0.997402
CAMK2B	94.23987	1.295459	4.080376	4.50E-05	0.002337
DNASE2	89.39118	-0.18843	-0.59347	0.552867	0.997402
TIPRL	93.90819	0.395104	1.244361	0.213367	0.997402
OXCT1	87.20909	-0.01718	-0.0541	0.956852	0.997402
UXS1	83.956	0.109884	0.346024	0.729325	0.997402
IWS1	85.83041	0.009843	0.030994	0.975274	0.997402
HLA-DPB1	90.56246	0.727315	2.28833	0.022118	0.36851

STX12	83.86332	0.427444	1.344623	0.178747	0.997402
SLC39A9	87.73809	-0.1352	-0.42523	0.670672	0.997402
COMMD4	85.29912	0.249689	0.784957	0.432479	0.997402
ABCA1	96.96895	-1.03772	-3.26191	0.001107	0.03641
RNF41	84.27724	-0.1612	-0.50671	0.612359	0.997402
RNF10	84.4999	-0.41111	-1.29193	0.19638	0.997402
SNRPF	84.62374	0.015998	0.050274	0.959904	0.997402
TKFC	86.67813	-0.22104	-0.6943	0.487492	0.997402
GALE	89.3471	0.537646	1.688743	0.091269	0.830663
MRPL20	83.79017	-0.2663	-0.83626	0.403008	0.997402
UBR7	84.07733	-0.06808	-0.21376	0.830733	0.997402
MICAL1	84.49377	-0.21969	-0.68927	0.490651	0.997402
ATP10A	89.70065	-1.29667	-4.068	4.74E-05	0.002456
KIAA1217	102.5211	-1.67819	-5.26374	1.41E-07	1.41E-05
PRPF3	83.98163	-0.27297	-0.85555	0.392246	0.997402
PHYKPL	83.61056	-0.1913	-0.59935	0.548941	0.997402
MCL1	85.14678	-0.36122	-1.13168	0.257771	0.997402
DMAC2	89.14907	0.275908	0.864034	0.387569	0.997402
PPIE	85.45693	-0.23413	-0.73298	0.463573	0.997402
VPS52	84.2412	-0.42136	-1.31896	0.187182	0.997402
TOP2A	85.1711	0.569669	1.78314	0.074564	0.755855
FURIN	88.05037	1.367972	4.280337	1.87E-05	0.00112
DAB2	85.78778	-0.17382	-0.54378	0.586591	0.997402
SLC35F5	88.31326	0.208759	0.653052	0.513723	0.997402
UBE2B	86.89357	0.126031	0.394251	0.693396	0.997402
HTRA2	86.22559	-0.292	-0.91312	0.361179	0.997402
SUMF1	83.12931	-0.01897	-0.05933	0.95269	0.997402
HNRNPD	91.46671	-0.00912	-0.02851	0.977252	0.997402
SLC6A6	83.45282	-0.02901	-0.09069	0.927738	0.997402
JAML	84.59601	-0.61004	-1.90704	0.056515	0.660725
SDSL	89.73513	-1.03368	-3.23106	0.001233	0.039403
AP5Z1	82.91203	0.042062	0.13144	0.895428	0.997402
SLC12A6	84.60317	0.243097	0.759569	0.447512	0.997402
NREP	88.40666	-0.59223	-1.84881	0.064485	0.707897
MAML1	84.90142	0.335831	1.048343	0.294481	0.997402
MMS19	89.03906	-0.3417	-1.06655	0.286173	0.997402
SNHG8	86.09001	0.288866	0.901578	0.367281	0.997402
AL354740.	91.14016	-0.20575	-0.64203	0.520855	0.997402
VPS13B	97.49675	-0.16933	-0.52837	0.597241	0.997402
PRELID1	125.7902	-0.39549	-1.23359	0.217354	0.997402
SACM1L	84.75776	0.569918	1.777359	0.075509	0.757442
SAMM50	84.57898	-0.06876	-0.21442	0.830217	0.997402
PEX2	86.50533	0.375971	1.172261	0.241092	0.997402
NQO1	83.46421	0.178304	0.555923	0.578264	0.997402
SLC10A7	92.91171	1.61523	5.035793	4.76E-07	4.19E-05
TMSB4XP8	83.92754	0.00785	0.02447	0.980477	0.997402
BAK1	84.82774	0.286961	0.894439	0.371087	0.997402
LINC00963	86.42262	-0.53767	-1.6754	0.093856	0.840425
TSTA3	82.67884	0.102156	0.318173	0.750353	0.997402
ATXN1	83.54295	0.159102	0.495467	0.62027	0.997402

MUL1	91.82908	-0.29331	-0.91295	0.36127	0.997402
NDFIP1	93.3518	-0.14235	-0.443	0.657766	0.997402
EIF5B	85.81425	0.213943	0.665602	0.505665	0.997402
HARS2	84.72379	0.203556	0.633231	0.526583	0.997402
EGFL7	85.72169	0.164988	0.513225	0.607794	0.997402
FAM149B1	100.3906	-0.26811	-0.83396	0.404303	0.997402
NAE1	86.30051	0.09671	0.300789	0.763575	0.997402
CYREN	87.86038	-0.23911	-0.7436	0.457119	0.997402
MAPK1	93.99563	0.195259	0.607222	0.543704	0.997402
SMIM7	82.69596	-0.03839	-0.11938	0.904976	0.997402
PNMA1	132.584	-0.60723	-1.88825	0.058992	0.67932
CES2	84.14249	0.101326	0.315077	0.752703	0.997402
CREB3	89.21962	-0.02065	-0.06419	0.948821	0.997402
PRPF4	81.51329	0.128578	0.399734	0.689353	0.997402
SELENOS	82.59434	-0.28119	-0.87414	0.382041	0.997402
IFNAR1	91.07902	0.148188	0.460679	0.645029	0.997402
APEX2	81.48245	0.129142	0.401463	0.688079	0.997402
MPHOSPH	82.01181	0.233335	0.725131	0.468372	0.997402
WDR83	87.91783	0.342248	1.06344	0.287582	0.997402
ST3GAL1	86.90365	-0.10972	-0.34092	0.733164	0.997402
NSF	82.35817	-0.04513	-0.14022	0.888483	0.997402
USP33	111.6624	0.126192	0.39199	0.695066	0.997402
EVL	93.91897	1.494367	4.641422	3.46E-06	0.000255
LAMA5	85.1617	0.014437	0.044827	0.964246	0.997402
BRD3	97.36573	-0.34998	-1.08666	0.277188	0.997402
SIRPA	82.47537	0.540609	1.678407	0.093268	0.839679
COASY	87.89967	0.254395	0.789756	0.42967	0.997402
TIMMDC1	91.59613	-0.23334	-0.72435	0.46885	0.997402
VPS39	98.59662	0.066207	0.205462	0.837211	0.997402
CYP20A1	82.15253	0.0506	0.157012	0.875235	0.997402
DHX8	102.8461	-0.02674	-0.08297	0.933878	0.997402
YTHDF3	86.15908	0.323172	1.002435	0.316133	0.997402
GBP2	96.31724	-1.94051	-6.01899	1.76E-09	2.33E-07
ACOX1	99.61375	0.488516	1.514862	0.129807	0.976074
GOT2	81.47167	0.022188	0.0688	0.945149	0.997402
TRPT1	84.01321	0.279537	0.866673	0.386121	0.997402
CHORDC1	86.44305	0.192805	0.597745	0.55001	0.997402
SEC61G	87.29786	-0.02417	-0.0749	0.940291	0.997402
MRPL57	84.33612	0.166214	0.515039	0.606526	0.997402
SUPT7L	81.89687	-0.0822	-0.25465	0.798996	0.997402
RSF1	83.67383	0.526052	1.629286	0.103252	0.885591
TM9SF4	99.48934	0.227288	0.703574	0.481698	0.997402
FNIP1	83.6019	-0.3127	-0.96785	0.333121	0.997402
EIF1AY	81.54227	-0.24889	-0.77029	0.441129	0.997402
AL355312.	81.36483	-0.22432	-0.69414	0.487595	0.997402
